# The past and future human impact on mammalian diversity

**DOI:** 10.1126/sciadv.abb2313

**Published:** 2020-09-04

**Authors:** Tobias Andermann, Søren Faurby, Samuel T. Turvey, Alexandre Antonelli, Daniele Silvestro

**Affiliations:** 1 Department of Biological and Environmental Sciences, University of Gothenburg, Göteborg, Sweden.; 2 Gothenburg Global Biodiversity Centre, Göteborg, Sweden.; 3 Institute of Zoology, Zoological Society of London, London, UK.; 4 Royal Botanic Gardens, Kew, Richmond, UK.; 5 Department of Biology, University of Fribourg, Fribourg, Switzerland.

## Abstract

To understand the current biodiversity crisis, it is crucial to determine how humans have affected biodiversity in the past. However, the extent of human involvement in species extinctions from the Late Pleistocene onward remains contentious. Here, we apply Bayesian models to the fossil record to estimate how mammalian extinction rates have changed over the past 126,000 years, inferring specific times of rate increases. We specifically test the hypothesis of human-caused extinctions by using posterior predictive methods. We find that human population size is able to predict past extinctions with 96% accuracy. Predictors based on past climate, in contrast, perform no better than expected by chance, suggesting that climate had a negligible impact on global mammal extinctions. Based on current trends, we predict for the near future a rate escalation of unprecedented magnitude. Our results provide a comprehensive assessment of the human impact on past and predicted future extinctions of mammals.

## Teaser

Human driven extinctions of recent past pale in comparison to future predictions, as a major global extinction wave is unfolding.

## INTRODUCTION

The current diversity of mammals consists of approximately 5700 extant species (*1*). In addition, at least 351 mammal species have gone extinct since the beginning of the Late Pleistocene 126 thousand years (ka) ago, 80 of which are known from historical reports (*1*) since the year 1500 CE (Common Era), while all others are only known from fossil or zooarcheological records (*2*). These rapidly increasing trends of mammalian species extinctions in the relatively recent past are matched by similar trends in other animal groups, such as birds, reptiles, amphibians, and ray-finned fishes, which lead scientists to declare the current biodiversity crisis (*3*).

To gauge the true severity of current rates of extinction, it is imperative to contrast these rates with natural, prehuman extinction rates. Several studies have addressed the question of the extent to which current extinction rates are elevated above background levels (*3*–*7*). However, these studies have assessed extinction rates with a taxonomic resolution of genera or families at a scale of extinctions per million years (Ma). Although the resulting rates are commonly used as proxies for species extinction rates, there is the potential for large discrepancies between rates calculated at different taxonomic levels and different time scales, because they are bound to underestimate the true rate on a species level. Furthermore, these previous studies have integrated rates over predefined time bins, which can inadvertently bias the resulting rate estimates (*3*, *8*). In a previous study, a mean species extinction rate of 0.249 extinctions per species per Ma (*9*) has been estimated for North American mammals across the entire Cenozoic period (past 66 Ma), yet temporally and taxonomically more fine-scale estimates of extinction rates are currently lacking.

For decades, scientists have debated to what extent humans have been driving species extinctions already in prehistoric times. Several studies have identified humans as the main driver of species extinctions since the beginning of the Late Pleistocene (*10*–*12*), mostly based on temporal associations between human arrival and extinctions of megafaunal species. Some authors have argued for strong human hunting pressure, particularly on megafauna mammals (*13*–*15*). Other studies have instead argued that there is insufficient archeological evidence for hunting of extinct mammal species to indicate human-caused extinctions, particularly at continental scales (*16*), and that the apparent temporal congruence could be due to other external factors. Continental extinctions during the past 126 ka are therefore sometimes attributed to major climatic and environmental fluctuations associated with glacial-interglacial cycles during the late Quaternary (*17*–*19*). However, other studies have concluded that the combined effect of humans and climate best explains past extinctions (*5*, *6*). These previous studies have focused exclusively on megafaunal extinctions, mostly restricted to the Pleistocene.

Regardless of the drivers of Pleistocene extinctions, the human impact in the most recent extinctions (since 1500 CE) is undeniable, and a central question concerns the effect that anthropogenically elevated extinction rates will have on future mammalian diversity. When generating future projections, it is important to consider that past extinction events only constitute a fraction of the true human impact on biodiversity (*20*). In addition to driving species to global extinction, human activity has led to decreases in population sizes and species ranges for a much larger fraction of mammalian species (*21*). Consideration of these trends is included in the International Union for the Conservation of Nature (IUCN) Red List criteria for evaluating the extinction risk of individual species (IUCN Red List Categories and Criteria, version 3.1). Previous studies have implemented different approaches to evaluate expected future biodiversity loss. Some scenarios predict substantial species losses in the near future, possibly reaching the levels of the five previous mass extinctions in Earth’s history within decades or centuries (*3*). However, several of these predictions are based on simplifying assumptions, for example, considering that all currently threatened species will inevitably become extinct within a few decades (*3*, *12*).

Here, we compile from the scientific literature the most recent fossil occurrences for all 351 mammal species that are known to have become globally extinct since the beginning of the Late Pleistocene, following the taxonomy from (*2*). To deal with the uneven and incomplete sampling in the fossil record (*22*), we estimated the times of extinction, which likely took place after the last occurrence, using estimated preservation rates. For any extinct species with confirmed occurrences since the year 1500 CE, we used these last confirmed observations instead of the fossil-based last occurrence dates. Our studied time period encompasses strong climatic fluctuations, including the Last Glacial Maximum and the preceding interglacial period that was similar to the Holocene climate of today. The time period also encompasses the expansion of *Homo sapiens* out of Africa and the subsequent colonization of all major landmasses. We apply our species-level dataset to evaluate statistically whether, and to what extent, species extinctions during the past 126 ka can be attributed to anthropogenic or to climatic factors, using a recently developed Bayesian algorithm for rate estimation from last occurrence data (*23*). We then apply the estimated rates to simulate future biodiversity loss and contrast these extinction rate–based predictions with predictions based on extinction probabilities associated with the IUCN conservation status of all current species.

## RESULTS

### Past extinction rate increases

**Fig. 1:**
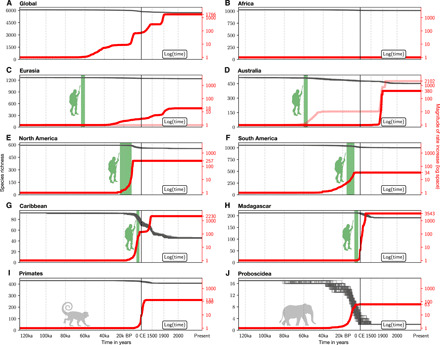
Different time periods of diversity decline and extinction rate increases between areas and orders. The plots show the declining diversity (black lines, 100 modeled extinction dates for each species) and the magnitude of extinction rate increases relative to the starting rate (red lines, mean values) through time, for all spatial (**A** to **H**) and two examples of taxonomic subsets (**I** to **J**) analyzed in this study. Extinction rates were estimated with a Bayesian rate-shift model, inferring the timing, number, and magnitude of shifts in extinction rates from the extinction dates of each subset. We calculated the mean marginal rates (harmonic mean) separately for all shift number models, which were supported by more than 10% posterior probability (table S3). The rate-shift model that was best supported by the data is shown in solid red, while the transparent red lines show the second-best model, if present. All rate estimates are transformed and plotted as the magnitude of extinction rate increase relative to the base value 126 ka ago. Note that the extinction rate axis (right, in red) is plotted in logarithmic space for better visibility. The time axis to the left of the solid vertical black line (0 CE) is plotted in units of ka before present (BP), while the time axis to the right of 0 CE is plotted in years CE in logarithmic space for better visibility of recent rate changes. Vertical columns shaded in green mark the times of first human arrival (if applicable).

On a global level, we find that current extinction rates are around 1700 times [1200 to 2300 times, 95% highest posterior density (HPD) interval] higher than those at the beginning of the Late Pleistocene (Fig. 1). A simple simulation shows how severe these current rates are: The 351 global mammal species extinctions that have occurred since the beginning of the Late Pleistocene would occur within only 810 years [95% confidence interval (CI), 500 to 1100 years] under the highly elevated current extinction rates, while they would take 1.75 Ma (CI, 0.95 to 2.99 Ma) if the extinction rates had remained unchanged since the beginning of the Late Pleistocene (fig. S1). The rate-shift model that was most supported by the global data in our analyses suggests four events of significant increases in extinction rates. The inferred times for these extinction rate increases are 63,800 to 32,200 years ago, 16,000 to 9500 years ago, 2300 to 600 years ago, and 180 and 120 years ago (HPD; Table 1).

**Table 1:** Timing of inferred shifts in extinction rates. The table shows the timing of the inferred extinction rate shifts (95% HPD range) for all data subsets analyzed in this study. For several subsets, there were multiple supported shift models, which differ in their inferred number of rate increases. All shift models that were supported by more than 5% posterior probability are shown here for each subset (see table S3 for overview of posterior probabilities for different models).

**Subset**	**First shift**	**Second**	**Third**	**Fourth**
Global (1)	32,150–63,792	9514–16,003	600–2292	117–182
Global (2)	26,476–56,650	5030–14,474	143–732	–
Africa (1)	–	–	–	–
Africa (2)	5501-36,342	–	–	–
Eurasia (1)	31–33,733	–	–	–
Eurasia (2)	–	–	–	–
Australia (1)	122–357	–	–	–
Australia (2)	43,514–65,035	124–216	–	–
North America	10,594–20,916	–	–	–
South America	7797–34,810	–	–	–
Caribbean	5347–15,428	568–1190	–	–
Madagascar	1396–4715	–	–	–
Carnivora	9599–28,849	–	–	–
Cetartiodactyla (1)	8689–19,826	–	–	–
Cetartiodactyla (2)	11,308–38,441	22–10,400	–	–
Chiroptera	133–15,450	–	–	–
Cingulata	9050–35,787	–	–	–
Diprotodontia (1)	70–8090	–	–	–
Diprotodontia (2)	41,621–62,624	74–685	–	–
Eulipotyphla	647–13,208	–	–	–
Peramelemorphia (1)	–	–	–	–
Peramelemorphia (2)	62–7214	–	–	–
Perissodactyla (1)	–	–	–	–
Perissodactyla (2)	11,668–33,558	–	–	–
Pilosa	6484–20,041	–	–	–
Primates	1278–7432	–	–	–
Proboscidea	6973–25,039	–	–	–
Rodentia	4550–16,996	505–1022	–	–

The timing of the first two identified rate shifts overlaps with times of colonization of new continents by *H. sapiens* (table S1), mainly Australia [human arrival: 65 to 44 ka ago; (*24*)] and the Americas [24 to 12 ka ago; (*25*)]. This is confirmed when analyzing the extinction record of these two continental regions separately (including only species endemic to each region), which shows evidence for extinction rate increases overlapping with the time of human arrival on each of these continents (Fig. 1). We estimated the earliest rate shift in Australia to have occurred between 65 and 44 ka ago (HPD), in North America between 21 and 11 ka ago, and in South America between 35 and 8 ka ago. The large uncertainty interval surrounding the South American rate increase may be a result of a generally sparse mammal fossil record of this continent, particularly in comparison to North America (fig. S3C), which affects the precision of the modeled extinction dates (fig. S2), thus increasing the uncertainty in the rate estimates.

Similar patterns have been found in previous studies (*6*, *11*, *13*), which argue that humans have had a large impact on the faunas of particularly Australia and the Americas, since mammals on these continents were behaviorally naïve to the presence of hominins and thus vulnerable to the appearance of *H. sapiens* as an efficient new predator. Similarly, these studies argue that mammals in Africa and Eurasia were ecologically adapted to predation by hominins through co-evolution, possibly dating back as far as several million years ago (*26*), and were thus more resilient to human hunting pressure in the late Quaternary, leading to fewer extinctions, consistent with our findings (Fig. 1).

Islands show similar overall patterns compared to the continents in terms of the effect of human arrival. Here, we analyzed two large biogeographically coherent island systems: Madagascar and the Caribbean. Estimated human arrival times for both of these island systems are placed around the same time, from 10 to 4 ka ago for Madagascar (*5*, *27*) and 7 to 4 ka ago for the Caribbean (*28*), although dates for both systems remain debated (*27*). We find strong support for an extinction rate increase on Madagascar following first human arrival (Fig. 1). Similarly, we find evidence for a rate increase in the Caribbean island system that largely overlaps with the proposed time frame of human arrival. However, this pattern is less obvious since the early boundary of the credible interval surrounding the proposed shift slightly predates the earliest proposed time of human arrival (Fig. 1).

Previous studies have found a strong size selectivity of mammal extinctions since the Late Pleistocene, with highest extinction rates for larger species (*5*–*7*, *11*, *12*). This bias likely reflects extinctions associated with humans, as archeological evidence demonstrates that humans hunted large-bodied mammals (*29*). While most of this pattern may be driven by body size, other traits such as population growth rate may also have been important (*10*). Most such traits cannot be reconstructed directly for extinct taxa, but they are all likely to have a strong phylogenetic signal (*30*). We therefore also conducted separate analyses for all mammalian orders. We find that the timing, number, and magnitude of extinctions vary strongly among mammalian orders (Tables 1 and 2, and data S1). For example, comparison between primates and proboscideans (elephants), two formerly widespread orders that differ greatly in average body size, shows significant differences in extinction rate dynamics, with proboscideans having experienced increased rates of extinctions since 25 ka ago, while primates experienced low levels of extinction until far more recently (7 ka ago; Fig. 1). In the past few thousand years, even the extinction rates for orders that mainly comprise smaller-bodied species, such as primates and rodents, began to rise (Table 1), probably in response to widespread habitat destruction from human land-use change rather than from direct hunting (*31*).

**Table 2:** Diversity, extinction rates, and magnitude of rate increase at different time points. The displayed extinction rate estimates are based on the shift model [reversible jump Markov Chain Monte Carlo (RJMCMC)], averaging across the complete posterior distribution (excluding 10% burn-in), and scaled in extinctions per species year (E/SY). The last two columns show the magnitude of extinction rate increase relative to the base value at 126 ka ago. Future diversity and rates are estimated from our “IUCN continuing trends” simulations.

**Subset**	**Diversity** **126 ka ago**	**Diversity** **present**	**Diversity** **2100 CE**	**Rate** **126 ka ago**	**Rate** **present**	**Rate** **2100 CE**	**Rate increase** **present**	**Rate increase** **2100 CE**
Global	6065	5714	5156	3.894 × 10^−8^	6.195 × 10^−5^	1.178 × 10^−3^	1591	30,260
Africa	1027	1012	929	1.249 × 10^−7^	1.249 × 10^−7^	9.690 × 10^−4^	1	7758
Eurasia	1268	1237	1134	1.194 × 10^−7^	1.468 × 10^−5^	1.005 × 10^−3^	123	8416
Australia	513	451	411	6.597 × 10^−7^	2.678 × 10^−4^	1.114 × 10^−3^	406	1688
North America	595	555	504	5.097 × 10^−8^	4.688 × 10^−6^	1.104 × 10^−3^	92	21,657
South America	1055	1001	908	1.086 × 10^−7^	2.386 × 10^−6^	1.116 × 10^−3^	22	10,270
Caribbean	92	45	37	3.873 × 10^−7^	5.298 × 10^−4^	2.568 × 10^−3^	1368	6631
Madagascar	212	191	154	7.842 × 10^−8^	4.627 × 10^−5^	2.697 × 10^−3^	590	34,398
Carnivora	277	255	235	9.495 × 10^−8^	5.112 × 10^−6^	9.230 × 10^−4^	54	9725
Cetartiodactyla	302	232	204	5.607 × 10^−7^	1.601 × 10^−5^	1.611 × 10^−3^	29	2874
Chiroptera	1153	1140	1056	2.661 × 10^−8^	8.216 × 10^−6^	8.680 × 10^−4^	309	32,611
Cingulata	39	20	19	1.319 × 10^−6^	3.264 × 10^−5^	1.473 × 10^−3^	25	1117
Diprotodontia	183	139	118	1.673 × 10^−6^	2.999 × 10^−4^	2.054 × 10^−3^	179	1227
Eulipotyphla	461	451	404	3.582 × 10^−8^	5.267 × 10^−6^	1.285 × 10^−3^	147	35,865
Peramelemorphia	24	19	17	1.981 × 10^−6^	1.981 × 10^−6^	2.356 × 10^−3^	1	1189
Perissodactyla	29	16	13	4.205 × 10^−6^	4.205 × 10^−6^	3.663 × 10^−3^	1	871
Pilosa	34	10	9	1.095 × 10^−6^	9.465 × 10^−5^	2.282 × 10^−3^	86	2084
Primates	430	407	337	1.238 × 10^−7^	1.499 × 10^−5^	2.366 × 10^−3^	121	19,101
Proboscidea	17	2	2	2.566 × 10^−6^	1.253 × 10^−4^	1.017 × 10^−2^	49	3961
Rodentia	2271	2197	1998	1.838 × 10^−8^	2.944 × 10^−5^	1.101 × 10^−3^	1602	59,905

### Causes of extinctions

The timing of the extinction rate shifts identified in this study coincides with human colonization patterns (Fig. 1). We further investigated this possible correlation in more detail to address the ongoing debate whether human or climatic causes, or the combination thereof, have been causing past extinctions. For this purpose, we applied a Bayesian correlation model, in which extinction rates are expressed as a linear or exponential function of a time continuous predictor. The tested predictors included human population size, human land occupation (i.e., the total area occupied by humans, including all major landmasses and islands), global temperature, and the magnitude of temperature change. We then applied the estimated extinction rates through time based on these predictors to simulate the diversity decline of mammals throughout the study time frame, starting with the value of 6065 species (mammal diversity 126 ka). Last, we compared the resulting diversity through time estimates with the empirical extinction data and calculated the mean absolute percentage error (MAPE) for all models. The accuracy scores reported in the following were computed by subtracting the MAPE values from 100% and constitute a measure of how well the extinction model predicts the past mammal extinctions (model adequacy).

**Fig. 2:**
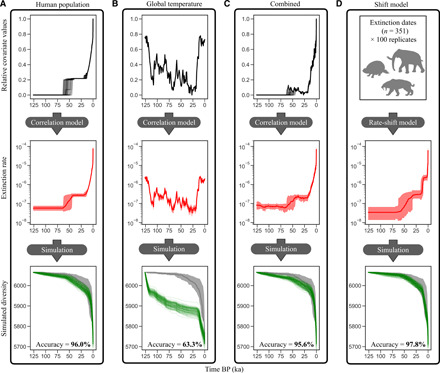
Higher model adequacy for human correlation model compared to climate model. The displayed models can be grouped into correlation models (**A** to **C**) in which extinction rates are estimated as a function of time continuous predictors and a rate-shift model (**D**) with a distinct and limited number of rate changes, estimated solely from the extinction dates dataset. The applied correlation variables were global human population density (A) and global mean temperature (B), as well as the interaction of the two in a mixed model (C). Shown for each model are the time-continuous predictor trajectories (black, top; only for correlation models), the estimated rates through time (red, middle; mean values and 95% HPD) and the simulated mammal species diversity based on the estimated rates (green, bottom). The accuracy scores in the bottom of the lower panels reflect how accurately the respective model predicted past extinctions and was calculated from the MAPE scores of each model (see. fig. S4). The input for the mixed model (C) included the product of human population density and global temperature, as well as each of these variables individually. See fig. S5 for further correlation models. The time axis is scaled in ka before present (BP).

Human population density as a single predictor explains the mammalian extinction patterns with 96.0% accuracy (94.6 to 98.0% CI; Fig. 2 and fig. S4). Similarly, human land occupation performs as a good predictor of past extinctions with 97.1% accuracy (94.6 to 98.9%), although it produces more biased diversity predictions toward the present, as the maximum value of this predictor has remained nearly constant for the past 10 ka (figs. S4 and S5). Climate predictors, on the other hand, lead to very low accuracy values, such as global temperature with 63.6% accuracy (57.0 to 68.2%) and the rate of temperature change with 60.2% accuracy (54.4 to 65.7%). These accuracy values only increase slightly when allowing for a temporal lag in the response of extinction rates to fluctuations in climate (figs. S4 and S5). The accuracy scores estimated for all climate models are not significantly different from our null model with 59.4% accuracy (53.1 to 64.5%; fig. S5), which is a constant model with no change of the predictor variable through time. This constant model represents an unlikely scenario where we assume that the extinction rate was constant throughout the entire time frame of this study in contrast with the multiple shifts in extinction rates inferred from the data (Fig. 1). The accuracy improves markedly to 97.8% when applying our rate-shift model (96.4 to 99.0%; Fig. 2 and fig. S4).

A mixed model including both human and climate predictors as well as their interaction (human population density and global temperature) performs similarly to the human correlation models with 95.6% accuracy (93.6 to 97.2%; Fig. 2). The correlation factors estimated for this model (fig. S6), which indicate the directionality (positive or negative correlation) and the strength of the effect of the predictor on past extinctions, are not significantly different from 0 (no correlation based on 95% HPD) for climate alone and for the interaction term between climate and humans, while the correlation factor for human population size is significantly higher than 0 (positive correlation based on 95% HPD). This indicates that, even in the mixed model, human population density is the overpowering predictor.

In addition, we tested predictor variables reflecting the effect of the entire genus *Homo* instead of that of *H. sapiens* alone. This resulted in slightly lower accuracy scores compared to the human predictors with 90.1% accuracy for hominin population density (88.1 to 92.4% CI; figs. S4 and S5) and 95.4% accuracy for hominin land occupation (91.9 to 97.7%).

To complement the MAPE accuracy scores with an additional statistic of model adequacy, we calculated the coefficient of determination (*R*^2^) as a measure of how much of the empirical extinctions is predicted by the model. These values show the same patterns as the accuracy scores, with significantly higher *R*^2^ values for human correlation models, while those of the climate correlation models were not significantly higher than that of the null model (fig. S7).

We emphasize that the accuracy scores of our correlation models reflect how much of the past extinction dynamics can be explained with only a single variable (e.g., human population density). In reality, the causes of extinctions are more complex and are not expected to be fully dependent on a single variable. Yet, our results show that human population growth and associated processes had a strong effect on mammal extinctions, while global climatic patterns, such as the last glacial maximum, leave no statistically detectable trace in the extinction record.

### Future predictions

To provide a basis to compare the historical anthropogenic effects with the ongoing biodiversity crisis, we predicted future diversity losses under two different types of scenarios for all geographic and taxonomic subsets, either based on extinction rates informed by past extinctions (paleo scenarios, for which we defined two scenarios) or based on the current threat status of species (current threat scenario). The current threat scenario is based on IUCN threat status information and models future extinctions while also accounting for expected future threat status changes. The two paleo scenarios, on the other hand, are only informed by the past extinction record and do not contain current threat status information. In one of these paleo scenarios, we simulate future species losses based on the current extinction rate as estimated from past extinctions (see rates in Fig. 1 and Table 2). In the other paleo scenario, we apply the estimated human correlation factor (fig. S6) together with future human population predictions of different landmasses to predict future species losses based on the expected increase of human populations.

**Fig. 3:**
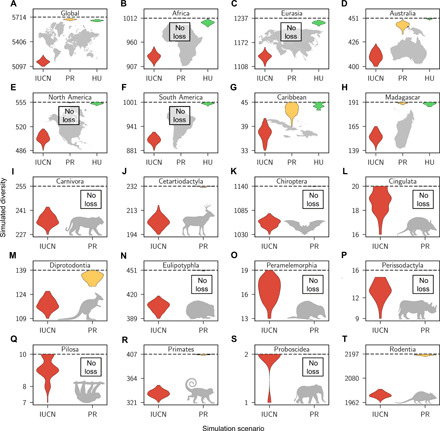
Substantial species losses predicted by year 2100 CE. The subplots show the estimates of mammalian species diversity globally (**A**) and for all spatial (**B** to **H**) and taxonomic subsets (**I** to **T**) analyzed in this study. The colored violin plots represent density plots of the 95% CI of diversity predictions based on the simulation scenarios “IUCN threat status prediction” (IUCN, red), “present extinction rate prediction” (PR, yellow), and “human population model prediction” (HU, green). In the first scenario (IUCN), we simulated extinctions based on the current threat statuses of species, applying extinction probabilities associated with these statuses. In the second scenario (PR), we applied current extinction rates as estimated from past extinction data. In the third scenario [only for spatial data subsets (A to H)], we simulated future extinction based on the correlation factor estimated for human population density combined with future human population predictions for different areas. The horizontal dashed lines show the current species diversity of each group. Note that the *y* axes only show a subsection of possible diversity values and do not include 0.

Particularly for the IUCN-based scenario, we predict substantial diversity losses across all orders and landmasses by the year 2100, the final year of our simulated time frame (Fig. 3). On the basis of the IUCN-based scenario, we predict 558 (CI, 502 to 610) mammal species extinctions globally by the year 2100. On average, we find that the IUCN-based future predictions lead to 5- to 35-fold more simulated extinctions than what would be expected based on current extinction rates estimated from past extinctions (Fig. 3).

**Fig. 4:**
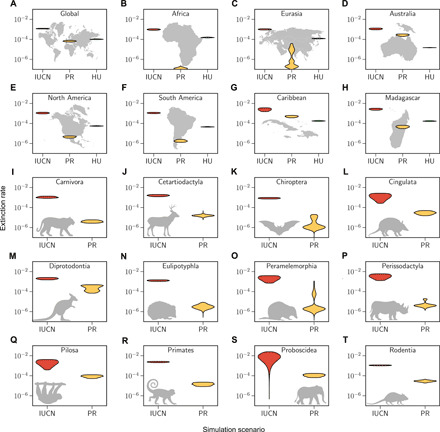
Expected increases in extinction rates for most orders and areas. In structure equivalent to Fig. 3, the violin plots (**A** to **T**) show the 95% HPD interval density of estimated extinction rates in the year 2100 based on the different diversity prediction scenarios. The IUCN rates (red) were estimated from simulated future extinctions based on the IUCN threat status of species in each subset. These extinction rate predictions are consistently higher than the present rates (PR) estimated from recent extinctions (yellow). For several spatial subsets (B,C,E,F, and H) we predict rate increases based solely on human population size increases (HU, green). Rates were estimated applying a shift model as implemented in PyRate. The multimodality of some rate distributions reflects the model uncertainty of the applied shift model.

Similar to the estimates of standing diversity, predicted extinction rates for the year 2100 are significantly increased in the IUCN-based scenario compared to the other two paleo scenarios across all taxa and regions (Fig 4). For most subsets, this predicted increase in extinction rates has a scale of several orders of magnitude; however, in particular, for Australia and the Caribbean, the current rates are already elevated to levels similar to the IUCN scenarios (Fig. 4). This suggests that, although the IUCN-based predictions appear severe, they are realistic, as some areas have already reached these elevated levels of extinction at present.

In particular, Africa and Eurasia have had comparably few recent species extinctions and therefore have low estimates of extinction rates at present, yet many of the currently extant species are severely threatened. This leads to large discrepancies between the current extinction rate and the rate predicted for the next 80 years under the IUCN scenario (Fig. 4). In particular, for Africa, we can see that the predicted future human population growth alone leads to significantly higher extinction rates compared to the extinction rates at present, without even considering the currently high threat status of so many species (Fig. 4). This indicates that human population growth will pose a serious threat for the current biodiversity in these regions.

## DISCUSSION

### Humans are the main driver of mammal extinctions since the Late Pleistocene

In this study, we estimate specific times of shifts in extinction rates from mammal extinctions since the beginning of the Late Pleistocene. These estimates fully incorporate uncertainties surrounding the dating of fossil occurrences, as well as the uncertainty involved when modeling extinction dates from fossil occurrences [e.g., the Signor-Lipps effect (*22*)]. By incorporating these uncertainties, we expect the differences in the temporal sampling density of extinction chronologies from different regions (i.e., some regions have a sparse fossil record, while others are more densely sampled) not to bias the resulting extinction rate estimates but instead to be captured by variation in uncertainty intervals surrounding these estimates. These extended uncertainty intervals surrounding the estimated time of rate shift can be seen for areas with a sparse fossil record, such as South America and the Caribbean. For other areas, however, particularly Australia, North America, and Madagascar, we find strong evidence for a distinct peak in extinction rates following human arrival, which complements the findings of previous studies (*5*, *6*).

Several lines of evidence support the hypothesis that the observed overlap of human arrival times and extinction rate increases in Australia and the Americas most likely represents a true causality rather than a result of external factors affecting both events (such as climatic variations and associated changes in sea level, enabling humans to colonize new landmasses while also causing mammal extinctions). First, our correlation models show that past climate fluctuations are a poor predictor of mammal extinctions (Fig. 2), although it is important to keep in mind that our tested predictor variable only capture global trends, which can differ from local climatic fluctuations (see discussion below). Second, there is no evidence of Late Pleistocene extinction rate increases in Australia before human arrival (Fig. 1), despite large fluctuations in climate, and there is compelling evidence that no major climate or habitat shifts occurred in Australia during the inferred time of extinction rate increase (*32*, *33*). Similarly, we find no evidence of rate increases before human arrival in the Americas (Fig. 1). Last, we do not find any evidence of extinction rate increases on other continents coinciding with the timing of the shifts in Australia and the Americas, which would be expected if large-scale changes in climate had caused the rate changes on these continents.

In this study, we tested different correlation models, including a mixed model that accounts for human and climate factors and the interaction between the two (Fig. 2). We find no detectable effect of climate on mammal extinctions throughout the studied time frame. These results stand in contrast to previous support of the hypothesis that the interaction of humans and climate has been driving mammalian extinctions (albeit limited to megafaunal genera) during this same time frame (*6*). Human population density increases, on the other hand, have had a large detectable effect on mammal extinctions, explaining past extinctions with ~97% accuracy.

Explaining a complex biological process such as extinctions with a single predictor, such as human population density, is arguably a great oversimplification and therefore is not expected to explain all of the past extinction dynamics. However, our results show that human population density has substantial predictive power over the process, probably because it is correlated with other anthropogenic factors such as more intensive hunting pressure, land use, ecosystem modifications, e.g., through the use of fire, and several cascading effects that result from human impact on the natural world.

Another necessary simplification of our correlation model analyses, due to the lack of data, is fitting a global temperature curve to the extinction, since climate change is expected to have affected species individually on regional scales. While we expect local fluctuations and differences in climate across the globe, which are not captured in our global models, we argue that the climate patterns based on combined data from Antarctic and Arctic ice cores (*34*) describe large-scale trends affecting all regions. Our correlation models are not affected by absolute temperature values, which certainly differ between regions. Instead, they assume that extinction rates around the globe follow relative trends in the data, which we expect to be shared by all regions (for example, we expect temperatures to decrease in all regions during times of global cooling). Therefore, we expect our findings of low adequacy of climatic correlation models to hold true in general, even if more regionally detailed paleoclimate data were available and applied in these models.

### The future of mammalian species diversity

Our future simulations show that we expect large increases in extinction rates by the year 2100 compared to the present, when accounting for the current threat status of species (Fig. 4). According to these models, the extinctions that have occurred in the past centuries only represent the tip of the iceberg, compared to the looming extinctions of the next decades. Our human impact has led to several species extinctions in the past but additionally has severely decimated the population sizes and habitats of many more. This impact on extant species, which is not incorporated when quantifying our human impact based on past extinctions alone, is sometimes referred to as extinction debt (*35*).

The extinction debt effect is expected to be substantial according to our future simulations. For example, on a global level, we would expect 30 (CI, 16 to 42) mammal species extinctions by the year 2100 based on current extinction rates; however, when accounting for the current threat level of species, we predict 558 extinctions (502 to 610; Fig. 3). This pattern is reflected in all analyzed subsets of the data and is particularly large for Africa, the Americas, and Eurasia, since current extinction rates for these continents are still at a comparatively moderate level, yet many species are severely endangered (Fig. 4). For all these continents, we also expect large biodiversity losses based on expected human population size increases, leading to significantly higher rates compared to the present (Fig. 4). Therefore, human population size increases will undoubtedly pose a serious challenge for the future conservation of biodiversity in these areas.

### Conclusions

Our analysis of the extinction record and previous studies (*5*, *6*, *11*) provide compelling evidence that humans have caused a substantial wave of extinction upon arrival on new landmasses for mammalian communities that were not adapted to large primates as efficient predators. Since then, we have increased our impact on the natural world, which, in the past centuries, has reached unprecedented scales to satisfy our increasing energy and resource usage in all parts of the world (*36*). We are losing biodiversity every year, and with every extinct species and population, we lose unique evolutionary history.

By the year 2100, we predict all areas of the world to have entered a second wave of extinctions. Our simulation results indicate that this additional wave of anthropogenic extinctions may be much greater than the currently increased rates, by several orders of magnitude (Fig. 4 and Table 2). We find that Australia and the Caribbean in particular have already today entered the second extinction wave (Fig. 4) based on the extinctions that have occurred during the past decades. This shows that, although our predicted future rates and associated biodiversity losses are shockingly high, they are within a realistic range, since we can already see these future scenarios being manifested in parts of the world.

The Intergovernmental Platform on Biodiversity and Ecosystem Services recently outlined the primary drivers of biodiversity loss by order of global importance, which included land and sea use change, direct exploitation of organisms, climate change, and pollution (*37*).

Despite the high level of current threat and grim future scenarios, there is still a window of opportunity to prevent many species extinctions by improving conservation efforts. Even maintaining species in their current IUCN threat categories and not increasing their future threats would prevent hundreds of predicted mammal species extinctions by the year 2100 (fig. S8). Recent years have shown many conservation successes, with some species moving toward less threatened IUCN categories (*1*). We hope that our alarming predictions will foster increased realization on the urgency and scale of the conservation efforts needed to safeguard the future of mammalian diversity.

## MATERIALS AND METHODS

### Mammal extinction dates

We downloaded a list of 351 mammal species that have gone extinct since the beginning of the Late Pleistocene (126 ka ago) from the PHYLACINE 1.2 database (*2*). We then carried out an extensive literature review for each of the 351 recently extinct mammal species, searching the peer-reviewed literature for the youngest available fossil occurrence or, when available, the last recorded sighting of the species (data S2) (*1*). All last occurrence dates were converted into years-before-2020 to ensure temporal consistency with the IUCN observation data (*1*). In the following, we refer to the year 2020 as *t*_0_, counting backward in time (e.g., *t*_520_= year 1500 CE), so that the following holds for any given last occurrence date (*t*_LO_):

$$t_{0}\leq t_{\text{LO}}\leq t_{126,000}$$

The last fossil occurrence of a taxon will likely precede its actual time of extinction, since incomplete preservation makes sampling the very last individual of a species highly unlikely [Signor Lipps effect; (*22*)]. This effect is expected to differ among species, since some species are common in the fossil record due to a high preservation potential, which makes it more likely to sample a last occurrence date closer to the actual extinction date, compared to species which are rare in the fossil record. We refer to this bias as the preservation bias, which we approximate for each individual species based on the sampling frequency of the species in the fossil record.

For this purpose, we compiled all mammal fossil records from the major public databases, namely, the Paleobiology Database (https://paleobiodb.org/), the New and Old Worlds database of fossil mammals (www.helsinki.fi/science/now/database.html), the Neotoma database (*38*), and the Sahul database (*39*). We merged all downloaded fossil occurrences into one shared database and removed all entries that were not identified to the species level. We corrected misspellings in species names, using the algorithm described in (*23*). Further, we removed potential duplicate records of the same fossil occurrence by only selecting unique occurrences after rounding the age of all fossils to thousands of years and the coordinates to full degrees. We used this merged fossil database to determine the sampling frequency of each species in the fossil record.

More specifically, for each extinct species, we searched our merged database for fossil occurrences from the Middle Pleistocene (0.781 Ma ago) or younger that were dated with at least 10-ka precision. For those species for which we found at least two such database records, we calculated the preservation rate (*q*) using the following formula

$$q=\frac{N-1}{t_{\text{FO}}-t_{\text{LO}}}$$

where *N* is the number of fossil occurrences of a given species divided by the time span between the first (*t*_FO_) and last occurrence (*t*_LO_) of this species found in the merged database (*40*, *41*). The rate *q* represents the inverse of the average waiting time between two fossils of a taxon (1/*q*). When determining these parameters, we did not include our manually compiled last occurrence dates for each species. Instead, we determined *t*_FO_ and *t*_LO_ based on the oldest and youngest fossil in our compiled fossil database and *N* as the total number of fossils in the database for a given species. However, the above formula only allows calculation of *q* for taxa with two or more fossil occurrences. To determine the preservation rate for those species, which did not have at least two fossil occurrences, we modeled the average waiting times for these species by determining a regression function between the log-transformed number of occurrences and the log-transformed average waiting times (fig. S3). The regression was performed on the inverse rates (i.e., the average waiting times) instead of the actual rates, because the harmonic mean (i.e., the inverse of the arithmetic mean of the inverse values) provides a better representation of the average rate. The resulting preservation rates for all extinct species reflect the frequency at which each species occurs in the fossil record. We then used the calculated preservation rates to model extinction dates from the last occurrence data for each species. We chose one of the two following approaches, depending whether the last occurrence date represented a fossil occurrence or if it represented a last sighting.

For all extinct species, which are only known from the fossil record [no confirmed sighting since 1500 according to (*1*)], we sampled the extinction time from an exponential distribution with rate *q* (preservation rate) truncated at the year 1500 CE

$$t_{e}\sim t_{\text{LO}}-T\text{Exp}(q,t_{520})$$

We truncated the distribution under the assumption that all species that have not been seen since the year 1500 were already extinct at that time.

For all extinct species with a sighting since the year 1500, we know that extinction did not occur before the recorded last sighting. However, because of the Lazarus effect (rediscovery of species thought to be extinct), we cannot assume with absolute certainty that the taxon is truly extinct (*42*). We know of at least four examples of mammalian species (*Burramys parvus*, *Solenodon cubanus*, *Phyllomys unicolor*, and *Cuscomys oblativa*), which have been sighted since the year 1500, were then believed to be extinct and were finally rediscovered during recent decades (*42*). Since the rediscovery of *C. oblativa* was relatively recent (2009), it is listed as extinct in this study to stay consistent with the PHYLACINE database. Given the 351 species that went extinct during the time frame of this study (the past 126 ka), the fraction of rediscovered species is approximately 1%. We therefore set the extinction time for species sighted since 1500 CE as

$$t_{e}=t_{\text{LO}}-z$$

where *z*~Exp(*r*) is sampled from an exponential distribution with rate parameter *r* such that $\int_{t_{\text{LO}}}^{t_{0}}\text{Exp}(x;r)\mathrm{dx}=0.99$, thereby allowing a 1% probability for each of these species of still being extant. We considered a species as extant when *t*_e_ < 0. To account for the dating uncertainty of the fossil occurrences and the stochasticity of our approach, we drew 100 independent extinction dates for each of the 351 extinct species (fig. S2). These 100 replicates of extinction dates for each species were used in downstream analyses to estimate extinction rates, using the software PyRate (*43*).

Additional to extinct species, PyRate takes into account information about extant taxa to estimate the magnitude of the inferred extinction rates relative to the number of species in the group. To ensure equal sampling of extinct and extant species, PyRate only allows inclusion of extant species if these species have at least one recorded fossil occurrence. Since our study is focused on a relatively short and recent time frame (in macroevolutionary terms), and since mammals are among the groups with the best paleontological record, we assume sufficiently complete sampling of extinct taxa and therefore also included all extant species into our extinction rate estimation. The input data for all PyRate analyses were 100 replicates of origination and extinction dates (“0” for extant species) for each species. We assume all species in our dataset to have originated before the beginning of the studied time frame and therefore did not model the speciation process during this short evolutionary time frame. The truncation of all lineages at 126 ka does not affect our extinction rate estimates, if we assume that extinctions are independent of lineage age. Although recent studies have pointed out age-dependent extinction effects (*41*, *44*), this process is described to mainly take place on time scales of millions of years and is thus not expected to play a role within the studied time frame.

### Spatial subsets

To generate geographic subsets of extant and extinct mammal species, we defined the following geographic regions, which are expected to constitute meaningful geographic entities for mammalian endemism with limited inter-region dispersal: Africa, Eurasia, Australia, North America, South America, Caribbean, and Madagascar (fig. S9). Additional to the mainland part of each of these landmasses, the defined regions also include islands that were most likely connected by a land bridge with the respective region during the last glacial maximum (separated by less than 110-m water depth at present), following the definition of land-bridge islands in (*45*).

For each of these regions, we extracted all endemic species based on the historic ranges of these species as modeled in (*21*). We downloaded these range data for all extant and extinct mammal species from the PHYLACINE database (*2*). The historic ranges available from the PHYLACINE database are based on the IUCN v2016-3 taxonomy. Therefore, any species that have been added by IUCN since v2016-3 are not included in the spatial subsets analyzed in this study, due to a lack of available historic range data.

Because of the limited resolution of the range data (~100 km × 100 km grid), several species were found to be present in cells that could not be unambiguously assigned exactly to one of our defined regions (fig. S9). This case occurred, for example, for many species present in the border region between Eurasia and Africa, as well as for species with a coastal range, including cells shared by the mainland and nearby (non–land bridge) islands. To resolve these ambiguous cases, we only counted a species as being present in a specific region if it occurred in nonambiguous cells of this region. To deal with species occurring on small islands, which sometimes are only made up off a single ambiguous cell, we assigned all species coded as “occurs only on isolated islands” in the PHYLACINE database as being endemic to islands. The number of endemic species (extant and extinct combined) for each landmass can be found in table S2. An overview of all cells not assigned to any of our areas of endemism is shown in fig. S9.

### Taxonomic subsets

We generated taxonomic subsets of the global mammal data, consisting of species divided into the 29 mammalian orders present between the late Pleistocene and today, with the taxonomy following (*2*). To ensure sufficient data for meaningful extinction rate estimates, we only analyzed orders with at least three extinct species and more than 10 species in total in all downstream analyses. We excluded the exclusively marine order Sirenia (manatees) and removed all cetaceans from the order Cetartiodactyla and all pinnipeds from the order Carnivora. This resulted in seven spatial and 12 taxonomic data subsets. Similar to the spatial subsets, we relied on taxonomic information that was only available for the IUCN v2016-3 taxonomy. Therefore, the taxonomic subsets do not include extant species that were recently added by IUCN.

### Human population size

We compiled the changes in human population size throughout the past 126 ka to be used as a predictor variable to explain mammal extinctions. We downloaded human population size data from the HYDE database (*46*) for all continents except Antarctica (Africa, Europe, North America, South America, Asia, and Australia), ranging from the year 10,000 BCE until today. To model the missing human population size data from the beginning of our time frame (126 ka ago) until 10,000 BCE, we modeled the increases of human population size after the arrival on a new continent. We made the following assumptions: (i) at the year 10,000 BCE, all continents had reached a temporal carrying capacity regarding human population size (given the technological status), and (ii) human population size increases logistically after human arrival in a new region with a rate as estimated in (*47*) for New Zealand’s Maori population. Given these assumptions, we modeled the human population size for each continent to follow a logistic population growth starting at the time of arrival, using a growth rate as estimated in (*47*) and a carrying capacity of the population size of the given continent at 10,000 BCE.

To estimate times of human arrival for each continent, we used the “Global early” arrival scenario from (*5*), which summarizes the ranges of published evidence for human arrival times on all major landmasses. From these ranges, we drew an arrival date for each continent from a uniform distribution *t*_A_ ~ *U*(*t*_min_, *t*_max_), where *t*_min_ and *t*_max_ are the minimum and maximum arrival dates according to the ranges compiled in (*5*). To generate global estimates of human population size, for each point in time, we summed the modeled population sizes of all continents. We repeated the steps above for 100 independent replicates to account for the uncertainty surrounding the human arrival dates on all continents (table S1). The final modeled human population size data were log-transformed before applying them in our correlation model (fig. S10).

In addition to the human (*H. sapiens*) population size data, we modeled another predictor dataset for the general global hominin population (genus *Homo*), since other hominin species may have exerted a similar hunting pressure on mammalian fauna (*48*). For this scenario, we assumed that the 10,000 BCE carrying capacity of a given landmass applies independently to which species or combination of species of the genus *Homo* was present. In other words, we expect a landmass to reach a carrying capacity of hominins soon after the first hominin species arrives on the respective landmass. While there may have been substantial differences in population densities and ecologies between hominin species, this is a necessary simplification in our modeling, and there is increasing evidence for different hominin species affecting other species in a similar way as *H. sapiens* (*26*, *49*). In our case, this affects the modeled population sizes of Europe and Asia, which, according to our assumptions, were already at their 10,000 BCE carrying capacity of hominins at the beginning of our studied time frame (126 ka ago), since Neanderthals and Denisovans were already present in Europe and Asia at that time. The resulting modeled hominin population size through time (100 replicates) can be found in fig. S10.

In this study, we do not model any speciation process, thereby assuming that no speciation has occurred within the past 126 ka and that all extant species have existed since the beginning of the studied time frame. However, we do know of one example of a very recent mammal speciation in case of the pygmy sloth, which has speciated on a tiny land-bridge island during the Holocene (*50*).

### Human land occupation

As an additional predictor variable, we also compiled the changes in the total area occupied by humans throughout the past 126 ka. For this purpose, we first defined major geographic regions of human occupation, similar to the regions defined in (*5*) with slight modifications as summarized below (fig. S9). For each region, we added all those major islands (exceeding 10,000 km^2^) that were most likely connected via a land-bridge connection with the respective region during the last glacial maximum (separated by less than 110-m water depth at present), following the definition of land-bridge islands in (*45*). This definition of land-bridge islands led us to define Sulawesi as its own entity [in opposition to (*5*) who assigned it to Indo-Malaya]. Further, the northern island of Japan (Hokkaido) is assigned to Siberia in our model, while the rest of Japan is assigned to its own region. Further differences in (*5*) are that New Guinea and Tasmania are joined with Australia into one landmass in our model, and several Eurasian and North American islands are merged with their connected landmasses. In addition, we added Greenland (excluding areas covered by inland ice), Iceland, and New Zealand as separate regions, since we have well-documented arrival dates for these islands (*51*–*53*). Last, we defined the state of Alaska as its own entity (Beringia), since there is evidence for early human occupation thousands of years before the colonization of the rest of the Americas (*25*, *54*). We defined and calculated the area for each of these regions (table S1) using the shape files defined for botanical regions provided by the World Geographical Scheme for Recording Plant Distributions.

Second, we compiled ranges of human arrival times for each of these regions from the Global early scenario in (*5*) and updated several of these ranges to incorporate recent evidence (*24*, *55*, *56*). We added arrival dates for the regions that were not defined in (*5*), namely, Greenland (*51*), Iceland (*52*), Sulawesi (*57*), and Beringia (*25*) (table S1). For all regions, we drew a random arrival date *t*_A_~*U*(*t*_min_, *t*_max)_ from the time interval of possible human arrival using a stepping stone model starting in Africa (adjacent areas are colonized in a chronological manner, e.g., no colonization of Siberia before Central Asia).

Third, after drawing human arrival dates for all regions, we modeled an instantaneous occupation of the complete area of the respective region, under the assumption that humans colonize the full extent of a new landmass almost immediately after colonization. While this is arguably an oversimplification in our model, this is reportedly the case for islands (*47*), and here, we assume that also for continents the expansion of humans (given no major dispersal barriers) happens very quickly, based on evidence from the Americas, which shows an almost instantaneous spread to South America after initial colonization of North America (*54*) (outside of Beringia). We repeated the random drawing of arrival dates and subsequent modeling of human land occupation 100 times to account for the uncertainty surrounding the human arrival dates for all regions (fig. S10).

In addition to the human land occupation data, we modeled another predictor dataset for the hominin land occupation through time. Land occupation was modeled in the same manner as for the human scenario, with the difference that, as well as Africa, the landmasses of Africa, Europe, Siberia, Central Asia, and Indo-Malaya were already occupied by hominins 126 ka ago. The resulting trajectory of hominin land occupation through time (100 replicates) can be found in fig. S10.

### Past climate data

Additional to the human predictor variables, we also compiled climatic data for the time frame of this study (the past 126 ka) from the Antarctic ice core chronology [AICC2012 (*34*)]. We used these data (scaled in deuterium content of water molecules) as a proxy for the global average temperature throughout this time frame. We converted these data into temperature values (in °C) relative to the present using the formula

$$T=\frac{y+440}{6.2}$$

where *y* is the ice core deuterium content data. We analyzed the temperature data in two different representations: (i) global temperature trajectory, as directly derived from the ice core data, and (ii) temperature change through time. To calculate the temperature change through time, we first ran a local regression (LOESS) model in R (v3.4.3) using the R-native LOESS implementation to evenly space the temperature data points in a 100-year frequency. We estimated an optimized smoothing factor (span) for this regression using the generalized cross-validation criterion as discussed in (*58*). Next, we calculated the temperature change at each given point in time by calculating the variance of the temperature data within a sliding window with a width of 10 ka. The resulting trajectories for global temperature and for global temperature change throughout the past 126 ka are shown in fig. S10.

### Correlation models

To statistically test to which extent humans and climate correlate with past mammal extinctions, we used the PyRateContinuous function as implemented in the Bayesian program PyRate (*43*). Under this model, extinction rates (μ) are expressed as a linear or exponential function of a time-continuous predictor. The extinction rate at time *t* under the exponential model is μ*_t_* = μ_0_ × exp (γν_t_), where μ_0_ is an estimated baseline rate, ν*_t_* is the value of the predictor, and γ is the estimated correlation parameter. The baseline rate and correlation parameter are estimated on the basis of our dataset of extinct and extant mammal species using Markov Chain Monte Carlo (MCMC).

For each predictor, we ran 100 separate analyses, using different pairings of the modeled predictor replicates and the global extinction data replicates. We then ran PyRateContinuous, estimating the correlation between extinction rates (ignoring speciation rates) and the time-continuous predictor with the exponential model (*-mSpEx -1 0*), and a normal distribution (SD = 100) as prior for the correlation parameters (*-pG 100*):

*PyRateContinuous.py -d extinction_data.txt -c predictor_data.txt -mSpEx -1 0 -use_hp 0 -pG 100*.

PyRateContinuous scales the values of each predictor array to values between 0.5 (maximum value) and −0.5 (minimum value), which avoids biases stemming from the absolute magnitude of the input values while maintaining the relative differences within the predictor array. We applied the estimated correlation factors (fig. S6) and baseline extinction rates (rate at time where transformed predictor = 0) of each replicate to calculate the extinction rates throughout the past 126 ka as estimated from the respective correlation model (Fig. 2 and figs. S5 and S6).

To assess whether additive effects of human population and global temperature and the interaction between the two improved the adequacy of the extinction model, we ran additional analyses in which both predictors, and their interaction were jointly analyzed. Under these models, the extinction rate at time *t* was (*59*)

$$\mu_{t}=\mu_{0}\times \text{exp}[\alpha \nu_{t}+\beta \nu_{t}+\gamma (\nu_{t}z_{t})]$$

Last, we modified the climate-dependent extinction models to allow for a time lag between changes in the predictor variable (global temperature or climate change) and the response in extinction rates. The model included the addition of one parameter quantifying the time lag (δ) such that μ*_t_* = μ_0_ × exp (γν_*t* − δ_). We estimated the time lag in years after assigning a uniform prior *P*(δ) ~ *U*(0,10000).

### Shift model

We used a reversible jump MCMC (RJMCMC) algorithm implemented in PyRate (*23*) to infer the number, timing, and magnitude of statistically significant extinction rate changes from our mammalian extinction data. In contrast to the correlation models described above, this model estimates the extinction rates and changes thereof exclusively from the extinction data and thus is not dependent on other variables.

We ran the main PyRate function for the global data and for each subset, for all 100 extinction data replicates, by running the RJMCMC algorithm (*-A 4*) for 10 million generations (*-n 10000000*), only sampling shifts in extinction rates while ignoring speciation (*-rj_bd_shift 1*) to reduce the number of parameters, and sampling the estimated rates (*-log_marginal_rates 0*) every 5000 generations (-s 5000):

PyRate.py -d extinction_data.txt -A 4 -n 10000000 -rj_bd_shift 1 -log_marginal_rates 0 -s 5000

Mean rate estimates and their 95% HPD intervals were calculated across the complete posterior distributions (excluding 10% burn-in) of all 100 separate analyses for each dataset.

The posterior distribution of the shift model contains MCMC logs that were generated under different numbers of rate shifts explored by the algorithm. To separate the inferences generated under each of the different number of shifts explored by the algorithm, we calculated marginal rates for all those shift-number models that were supported by at least 5% Bayesian posterior probability (table S3). From these marginal rates, we calculated the relative magnitudes of rate change for each data subset (Fig. 1) by dividing the extinction rate at a given time point (μ_t_) by the extinction rate at the beginning of the time frame (μ_126,000_). Further, we extracted the times of rate shifts for the selected shift-number models by averaging over the times of shift for all MCMC generations sampled under the respective shift-number model (Table 1).

### Model adequacy tests

To assess the adequacy of the correlation and shift models, we evaluated how well the estimated extinction rates through time can reproduce the empirical extinction data. For this purpose, we first simulated the past diversity trajectory for mammals throughout the past 126 ka using the extinction rates through time as inferred under each model. We used a starting diversity of 6065 species (5714 extant + 351 extinct mammal species) and simulated extinctions in 100-year intervals (*n* = 1260) from 126 ka ago until present, using the corresponding extinction rate for each time interval as estimated by the respective model. Next, we compared the mean values of the empirical diversity and the simulated diversity (Fig. 2, bottom) at each point in time. We calculated the error between the simulated (*D*_sim_) and empirical diversity (*D*_emp_) relative to the total number of extinctions (*N* = 351) for each time bin (fig. S4)

$$E_{t}=\frac{D_{\text{sim}}-D_{\text{emp}}}{351}$$

Next, we calculated the MAPE from the absolute errors of each simulated diversity curve

$$\text{MAPE}=100\times \frac{\sum_{t_{0}}^{t_{n}}\text{abs}(E_{t})}{n}$$

From the 100 simulation replicates of each model, we calculated the mean MAPE score and the 95% CI. To transform the MAPE values into accuracy values, we subtracted them from 100%, which are the values reported in the text and figures (Fig. 2 and fig. S5).

### Future diversity predictions

We projected future species losses based on the extinction rate at present (“Present rate”) as estimated from the past extinction data resulting from our shift model (table S3). We extracted the mean values of the shift model results of all 100 data replicates of each subset (complete posterior distribution, excluding 10% burn-in) and simulated future extinctions under these rates in 1-year intervals, assuming the rates to remain stable until the year 2100 CE.

In a second scenario, we modeled expected future extinctions based on the human correlation factor (“Human pop. model”) that we estimated in this study (fig. S6). For this purpose, we extracted the mean values of the correlation factor estimates between human population density and mammal extinction rates from our 100 separate analyses of each data subset. We then compiled yearly future predictions of human population sizes until the year 2100 for all landmasses from Our World in Data (https://ourworldindata.org/world-population-growth) and calculated the future human population density trajectories based on these population sizes and the surface areas of each of our defined landmasses (table S1). Using the extracted correlation factors and the human population density predictions, we modeled future extinction rates until the year 2100 CE. We then used these modeled future rates to simulated future species extinctions in yearly intervals, applying the respective extinction rate at each time interval.

These two described scenarios project future extinctions based on rates that were estimated from past extinctions. However, besides having driven many species to extinction, human impact has led to massive population size decreases and habitat destructions for many species, leaving a large fraction of the currently extant species at a high risk of extinction [see IUCN assessments (*1*)]. Therefore, we expect any future projections that do not include these aspects, to underestimate the number of expected extinctions. To account for the current threat status of species when simulating future diversity losses, we used the software iucn_sim (*60*), which automatically accesses any available IUCN threat status information of the provided species (*1*). This software estimates species-specific extinction probabilities based on the current IUCN status in combination with user-provided generation length (GL) data for each species, and it additionally models future changes in IUCN status based on status transition rates estimated from the IUCN record of the past decades (*60*).

To provide estimates of GL for all mammal species for iucn_sim, we first compiled all available GL data from (*61*). For all remaining species, we estimated GL values based on a phylogenetic imputation using the R package Rphylopars (*62*) under the assumption that GL has a phylogenetic correlation. For this purpose, we downloaded phylogenetic trees (1000 samples of posterior distribution) from the PHYLACINE database. These phylogenies contained 94% of the extant mammal species names listed in IUCN v2019-2. For these species, we repeated the phylogenetic imputation for 100 randomly selected trees from the downloaded tree distribution. For all remaining species that were not present in the phylogeny, we modeled the GL value to be the mean of the whole genus, calculated separately for each of the 100 GL data replicates.

Using iucn_sim, we estimated status transition rates across the whole class Mammalia and provided our GL estimates for all mammal species for calculating species-specific extinction probabilities for the threatened IUCN categories (*60*). These transition rates and extinction probabilities were then used to predict extinctions throughout the next 80 years across 10,000 simulation replicates. In addition, to simulating the iucn_sim default scenario (scenario 1), allowing for future status changes using the transition rates of the past decades (results shown in Fig. 3), we simulated other potential future scenarios implemented in iucn_sim (results shown in fig. S8): scenario 2: stable status, no future status changes; scenario 3: no additional threats, setting all transition rates to 0 that lead to worsening of threat status; scenario 4: no conservation, setting all transition rates to 0 that lead to improvement of threat status; and scenario 5: 10× conservation increase, multiplying all transition rates by factor 10 that lead to improvement of threat status.

### Future rate estimation

We compared the extinction rate estimates for the year 2100 between our three main simulation scenarios displayed in Fig. 3 (“IUCN continuous,” Present rate, and Human pop. model). For the Present rate scenario, we summarized the posterior distribution of the rate at present across all 100 shift model analyses of the past extinction data. For the Human pop. model scenario, we extracted the posterior distribution of extinction rates in the year 2100 as modeled with the correlation factor estimates and our future human population density predictions. For the IUCN continuous scenario, we analyzed the simulated times of extinction of all future extinct taxa in this scenario, using a fixed shift model (*-A 0*) implemented in PyRate (*43*) to estimate one constant rate for the 80 years between the years 2020 and 2100 (*-edgeShift 80 0*):

PyRate.py -d future_extinction_dates.txt -A 0 -edgeShift 80 0

## Supplementary Material

abb2313_Data_file_S1.pdf

abb2313_Data_file_S2.pdf

abb2313_SM.pdf

## SUPPLEMENTARY MATERIALS

Supplementary material for this article is available at http://advances.sciencemag.org/cgi/content/full/6/36/eabb2313/DC1

## Appendix

View/request a protocol for this paper from *Bio-protocol*.
