# Supplementary material for: The past and future human impact on mammalian diversity
Source: Sci Adv. 2020 Sep 4;6(36):eabb2313. doi: 10.1126/sciadv.abb2313 (PMC7473673; doi:10.1126/sciadv.abb2313)
Supplement: abb2313_Data_file_S1.pdf [file abb2313_Data_file_S1.pdf]

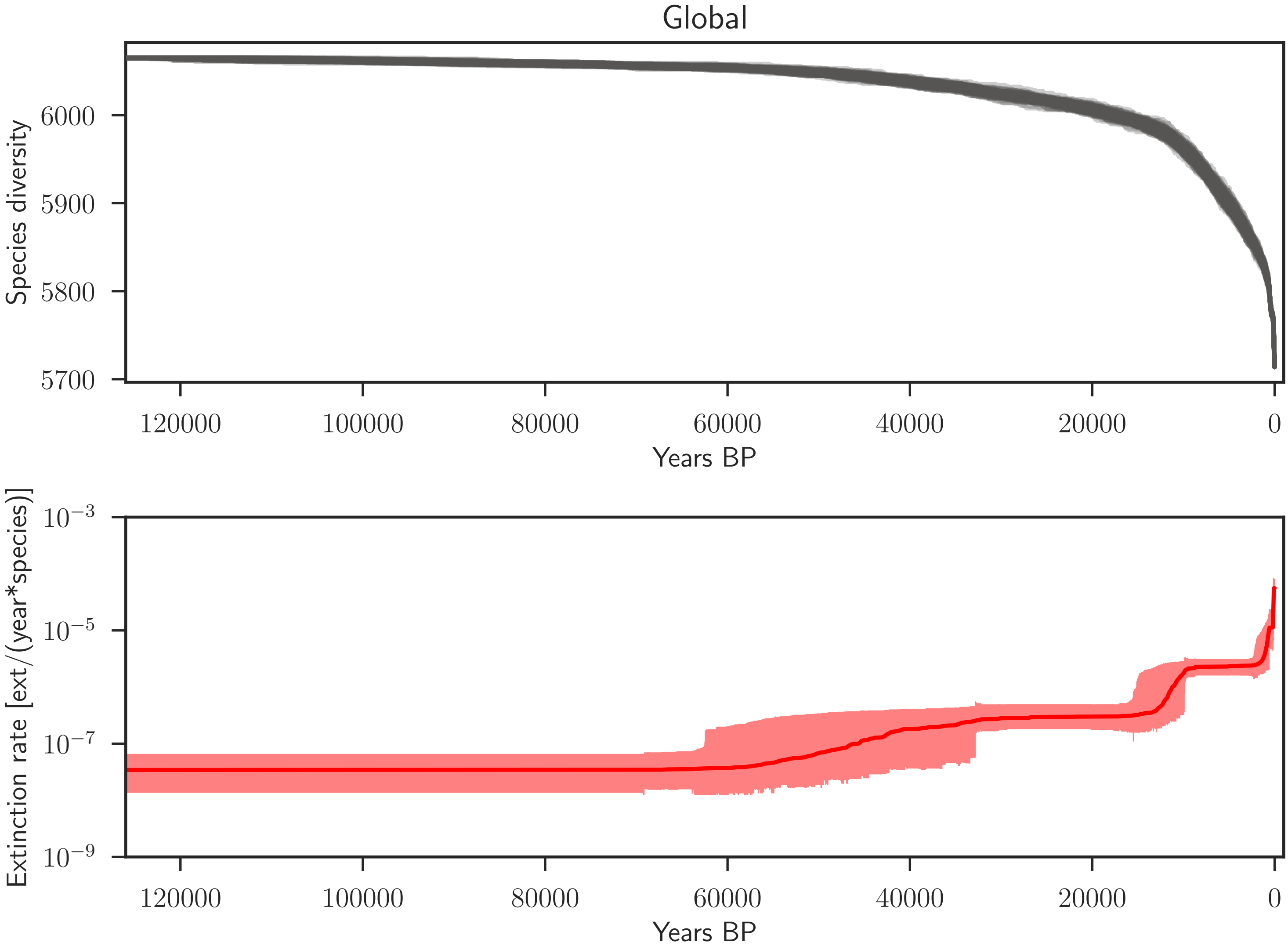

Global
Species diversity
6000
5900
5800
5700
120000
100000
80000
60000
40000
20000
0
Years BP
Extinction rate [ext/(year*species)]
10^{-3}
10^{-5}
10^{-7}
10^{-9}
120000
100000
80000
60000
40000
20000
0
Years BP

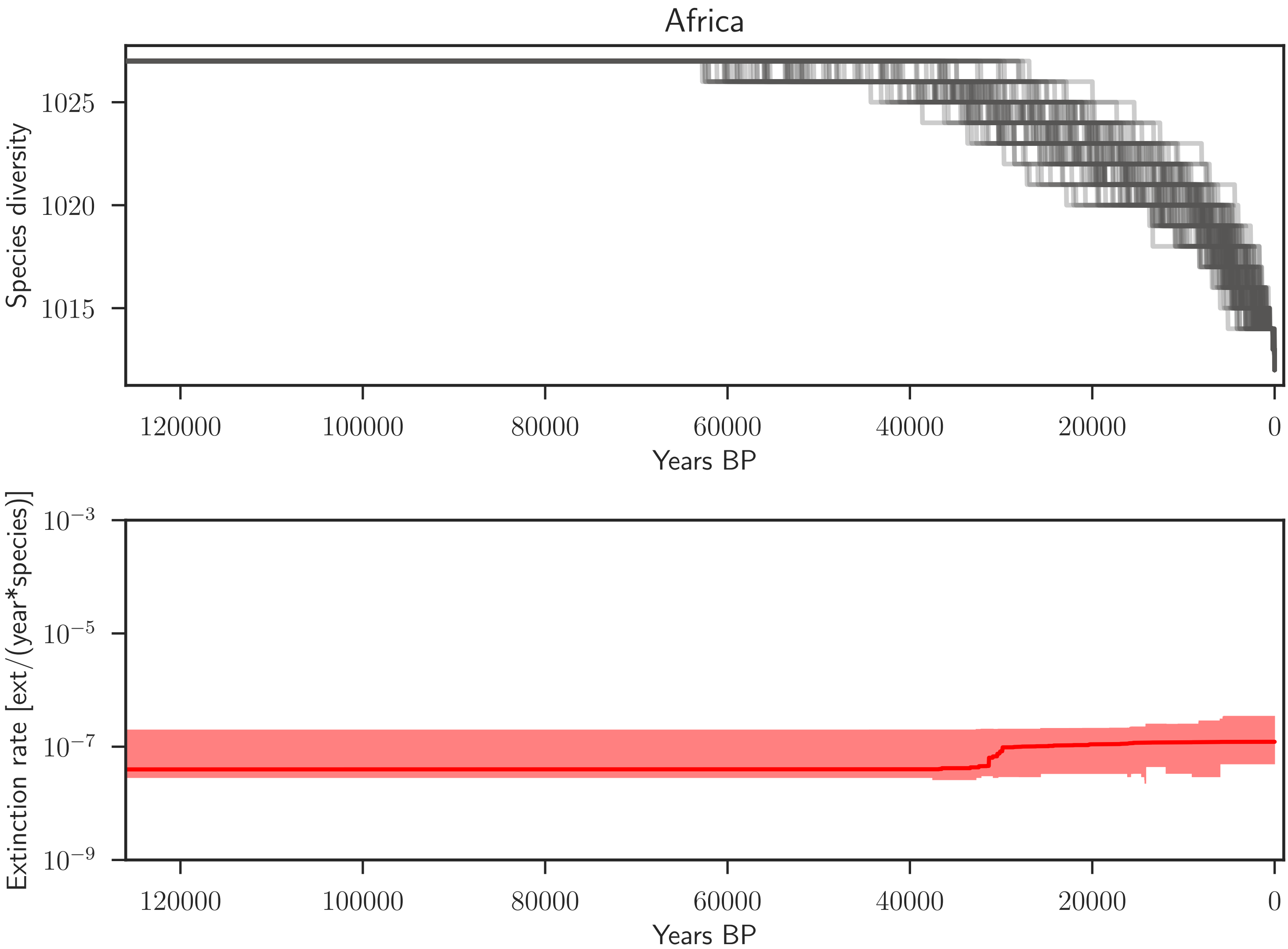

Africa
Species diversity
1025
1020
1015
120000
100000
80000
60000
40000
20000
0
Years BP
Extinction rate [ext/(year*species)]
10^{-3}
10^{-5}
10^{-7}
10^{-9}
120000
100000
80000
60000
40000
20000
0
Years BP

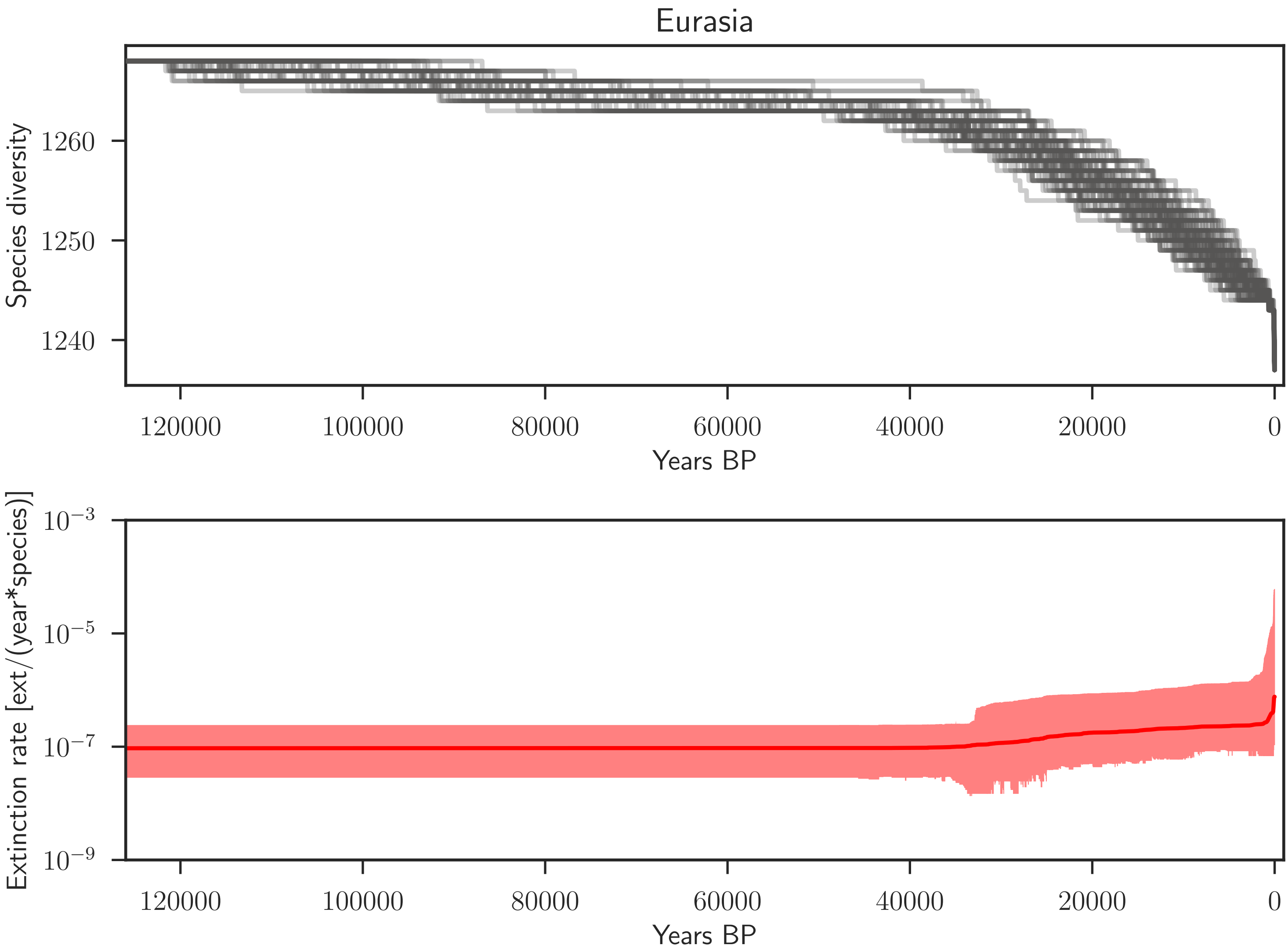

Eurasia
Species diversity
1260
1250
1240
120000
100000
80000
60000
40000
20000
0
Years BP
Extinction rate [ext/(year*species)]
10^{-3}
10^{-5}
10^{-7}
10^{-9}
120000
100000
80000
60000
40000
20000
0
Years BP

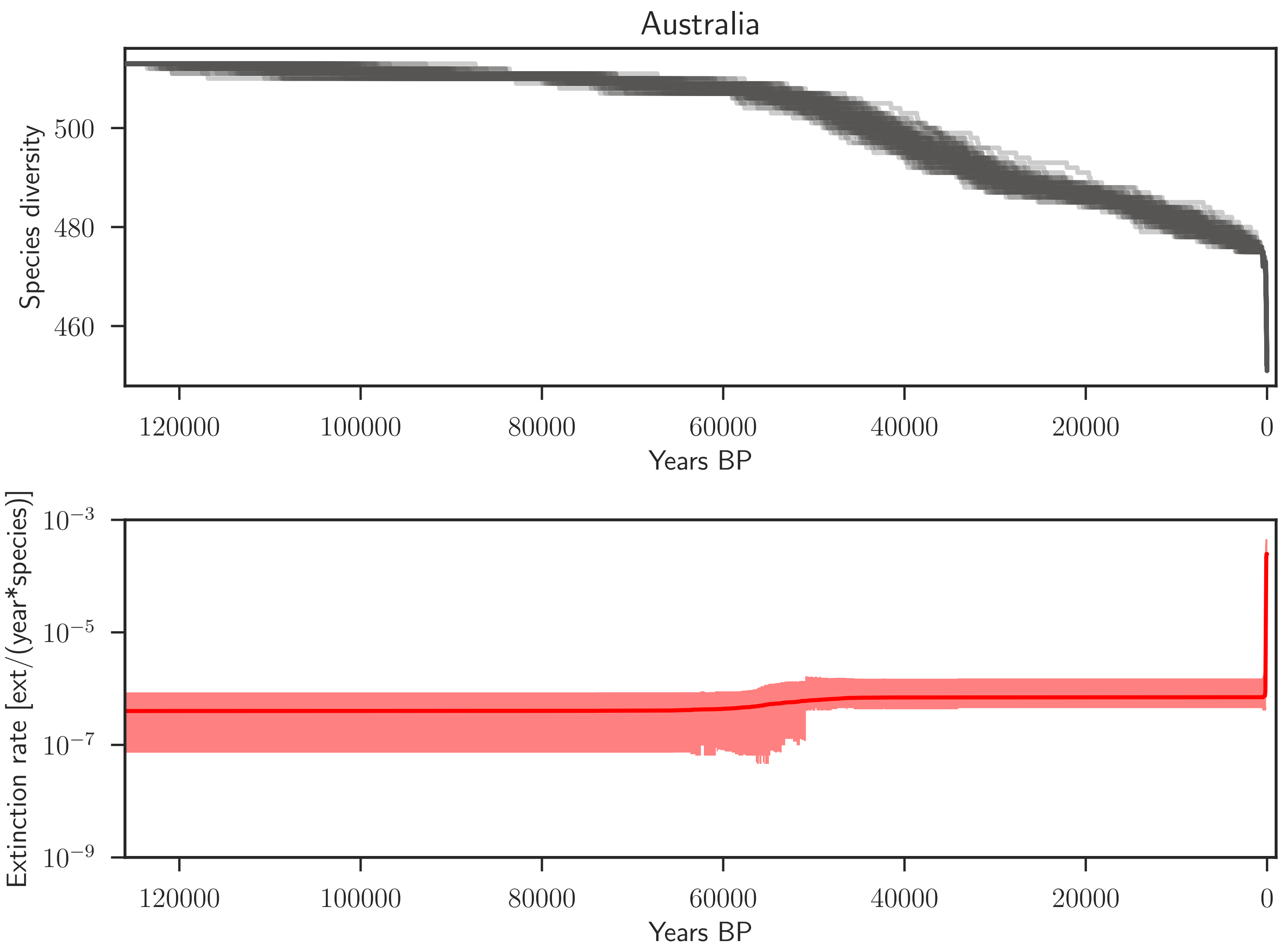

Australia
Species diversity
500
480
460
120000
100000
80000
60000
40000
20000
0
Years BP
Extinction rate [ext/(year*species)]
10^{-3}
10^{-5}
10^{-7}
10^{-9}
120000
100000
80000
60000
40000
20000
0
Years BP

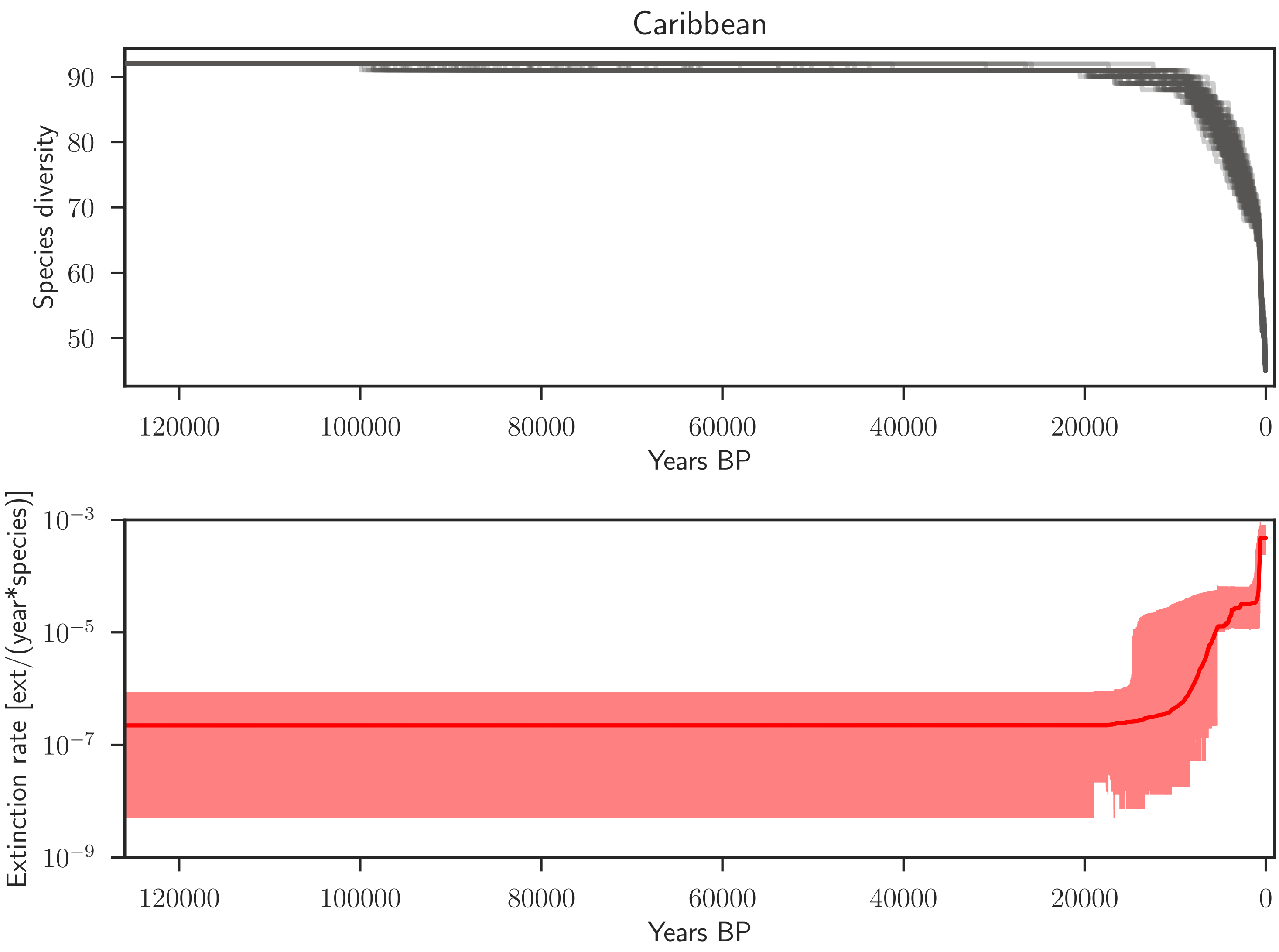
Caribbean
Species diversity
90
80
70
60
50
120000
100000
80000
60000
40000
20000
0
Years BP
Extinction rate [ext/(year*species)]
10^{-3}
10^{-5}
10^{-7}
10^{-9}
120000
100000
80000
60000
40000
20000
0
Years BP

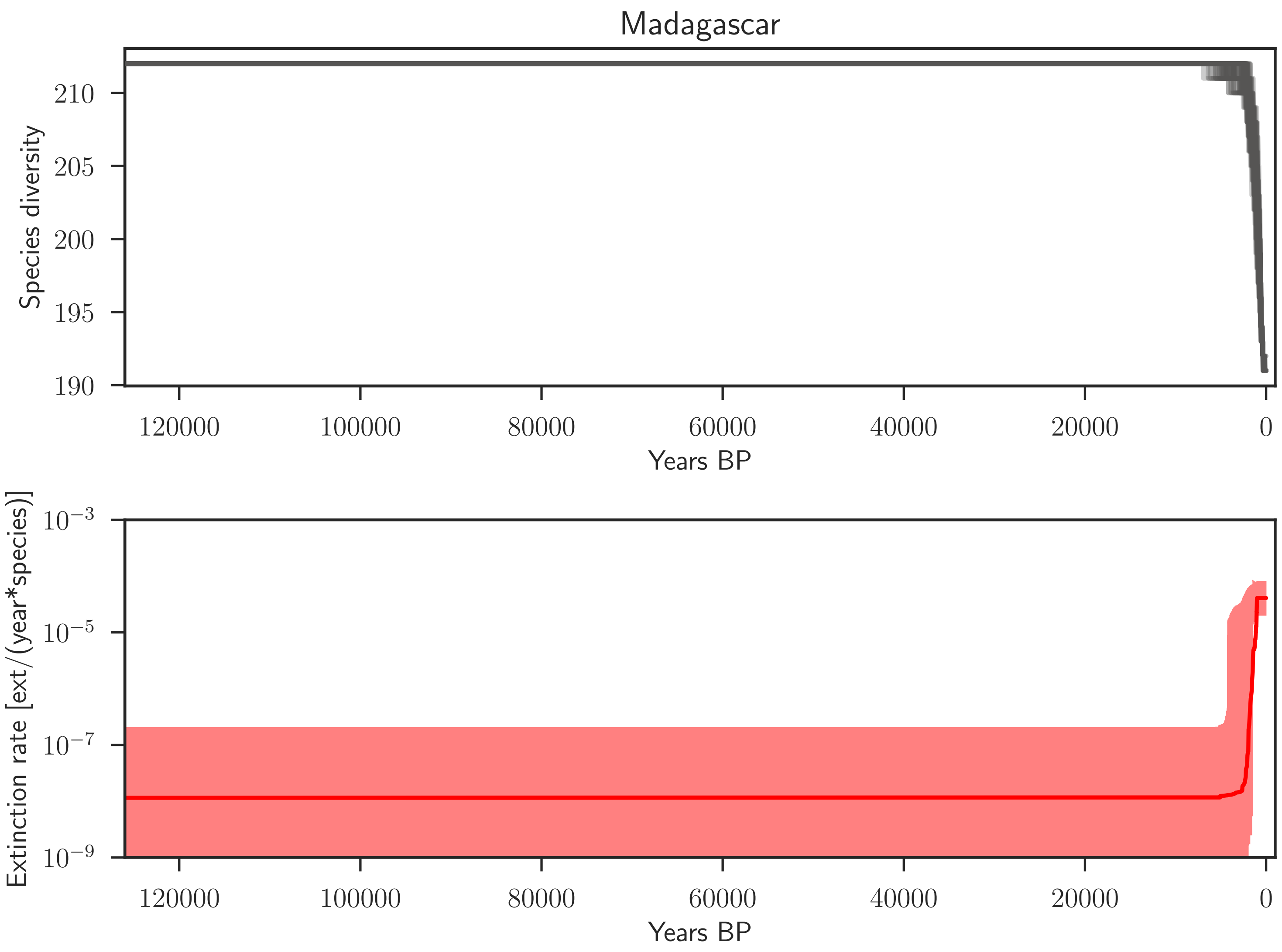

Madagascar
Species diversity
210
205
200
195
190
120000
100000
80000
60000
40000
20000
0
Years BP
Extinction rate [ext/(year*species)]
10^{-3}
10^{-5}
10^{-7}
10^{-9}
120000
100000
80000
60000
40000
20000
0
Years BP

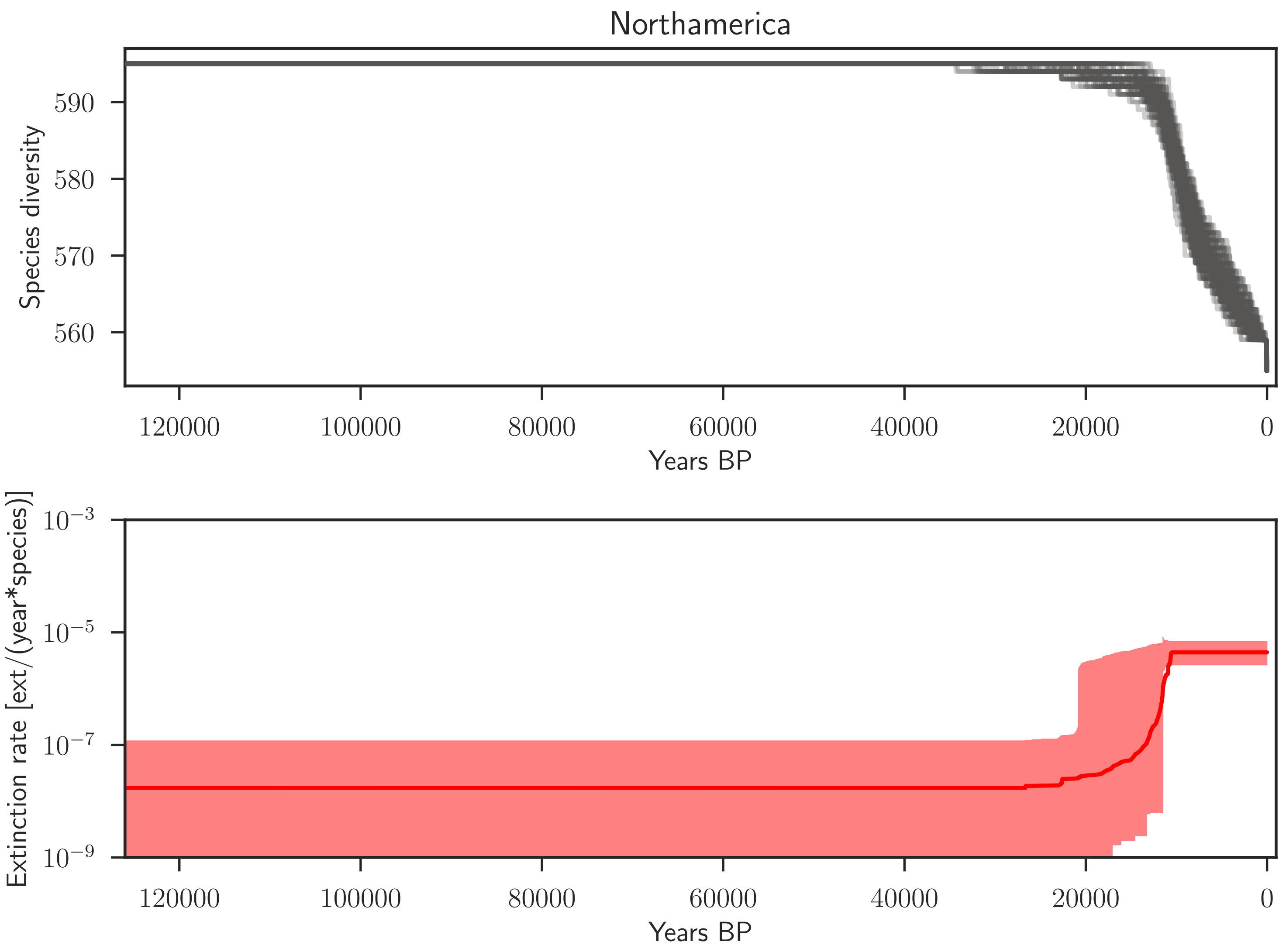

Northamerica
Species diversity
590
580
570
560
120000
100000
80000
60000
40000
20000
0
Years BP
Extinction rate [ext/(year*species)]
10^{-3}
10^{-5}
10^{-7}
10^{-9}
120000
100000
80000
60000
40000
20000
0
Years BP

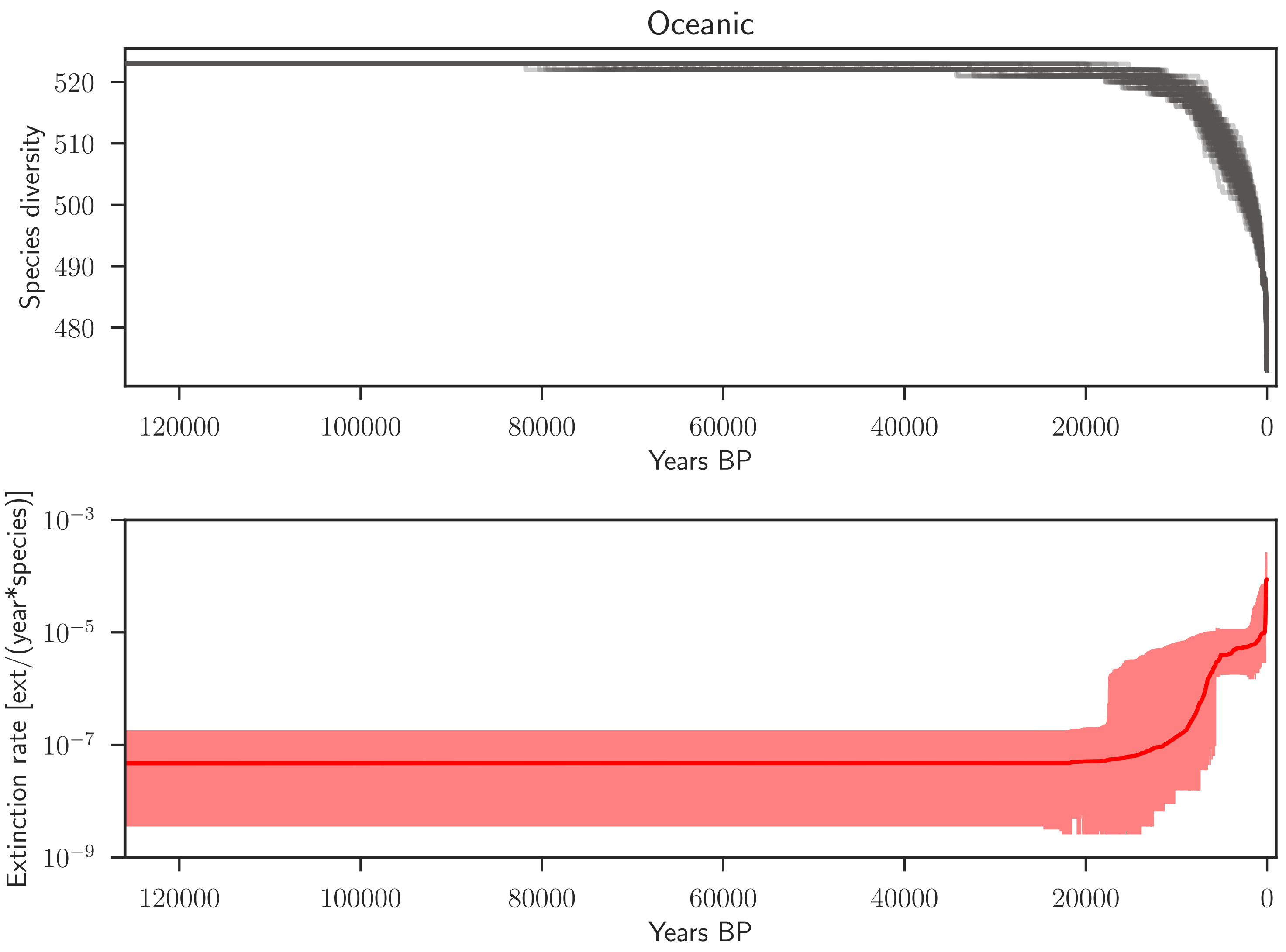

Oceanic
Species diversity
520
510
500
490
480
120000
100000
80000
60000
40000
20000
0
Years BP
Extinction rate [ext/(year*species)]
10^{-3}
10^{-5}
10^{-7}
10^{-9}
120000
100000
80000
60000
40000
20000
0
Years BP

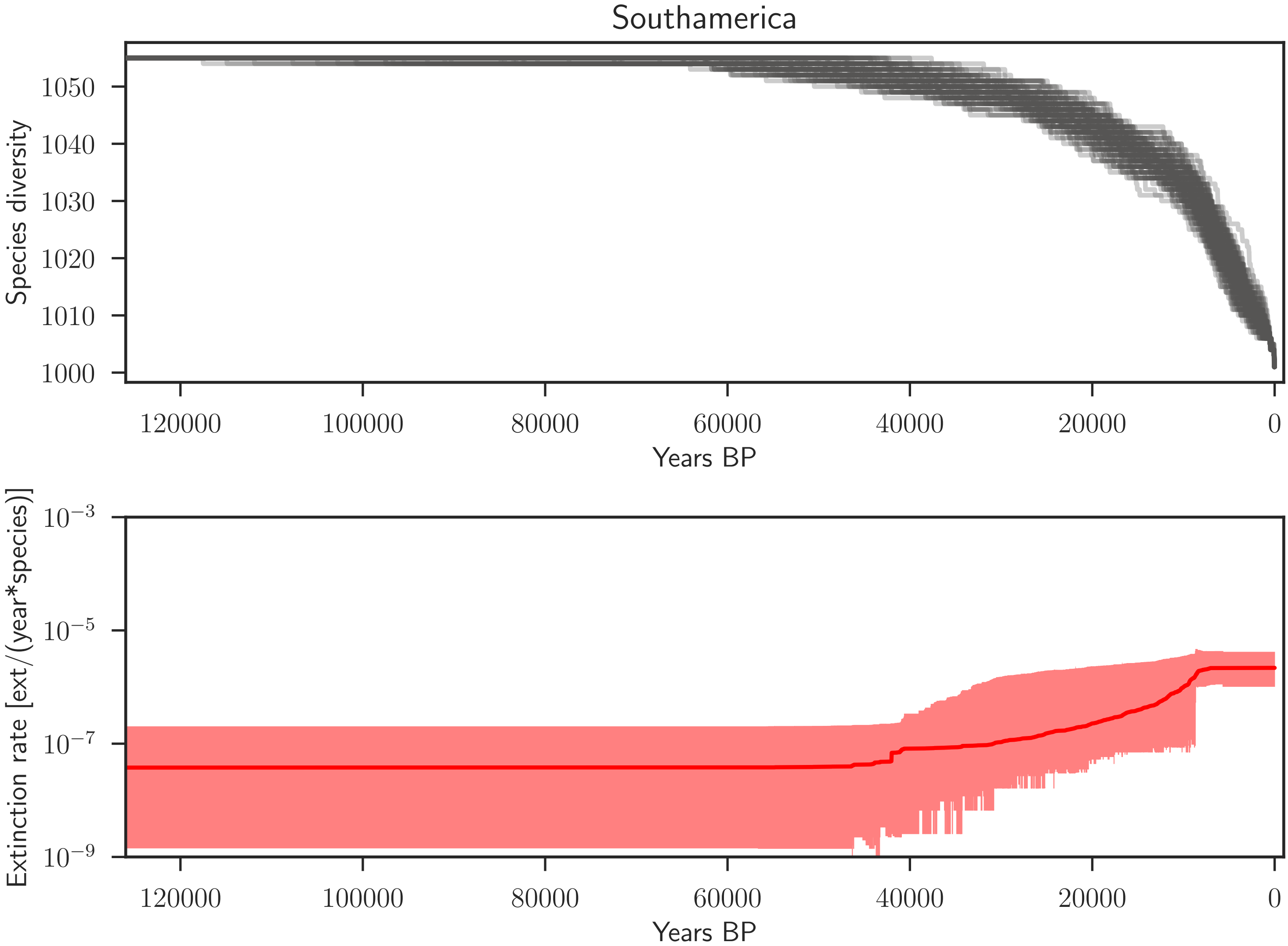

Southamerica
Species diversity
1050
1040
1030
1020
1010
1000
120000
100000
80000
60000
40000
20000
0
Years BP
Extinction rate [ext/(year*species)]
10^{-3}
10^{-5}
10^{-7}
10^{-9}
120000
100000
80000
60000
40000
20000
0
Years BP

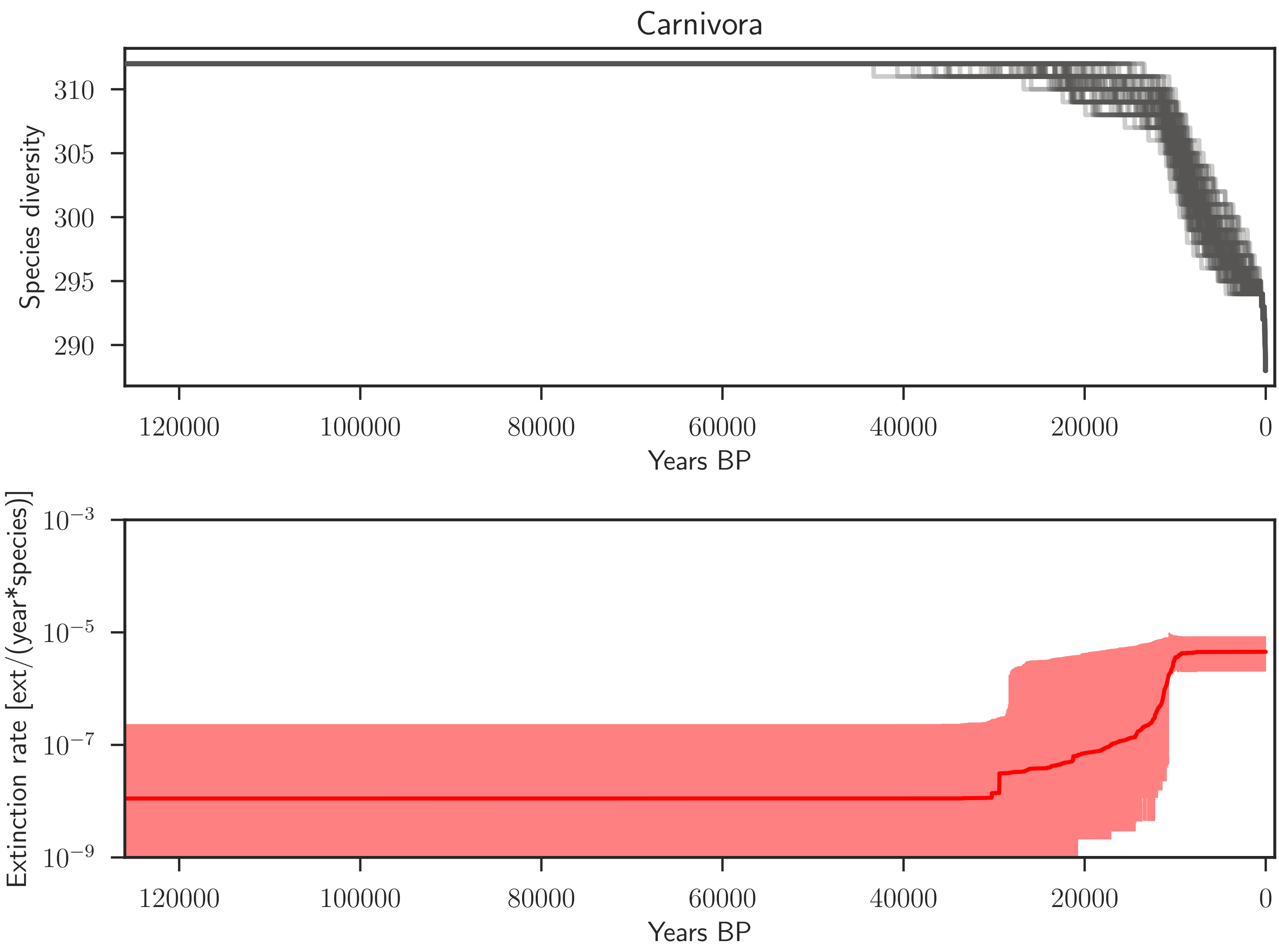

Carnivora
Species diversity
310
305
300
295
290
120000
100000
80000
60000
40000
20000
0
Years BP
Extinction rate [ext/(year*species)]
10^{-3}
10^{-5}
10^{-7}
10^{-9}
120000
100000
80000
60000
40000
20000
0
Years BP

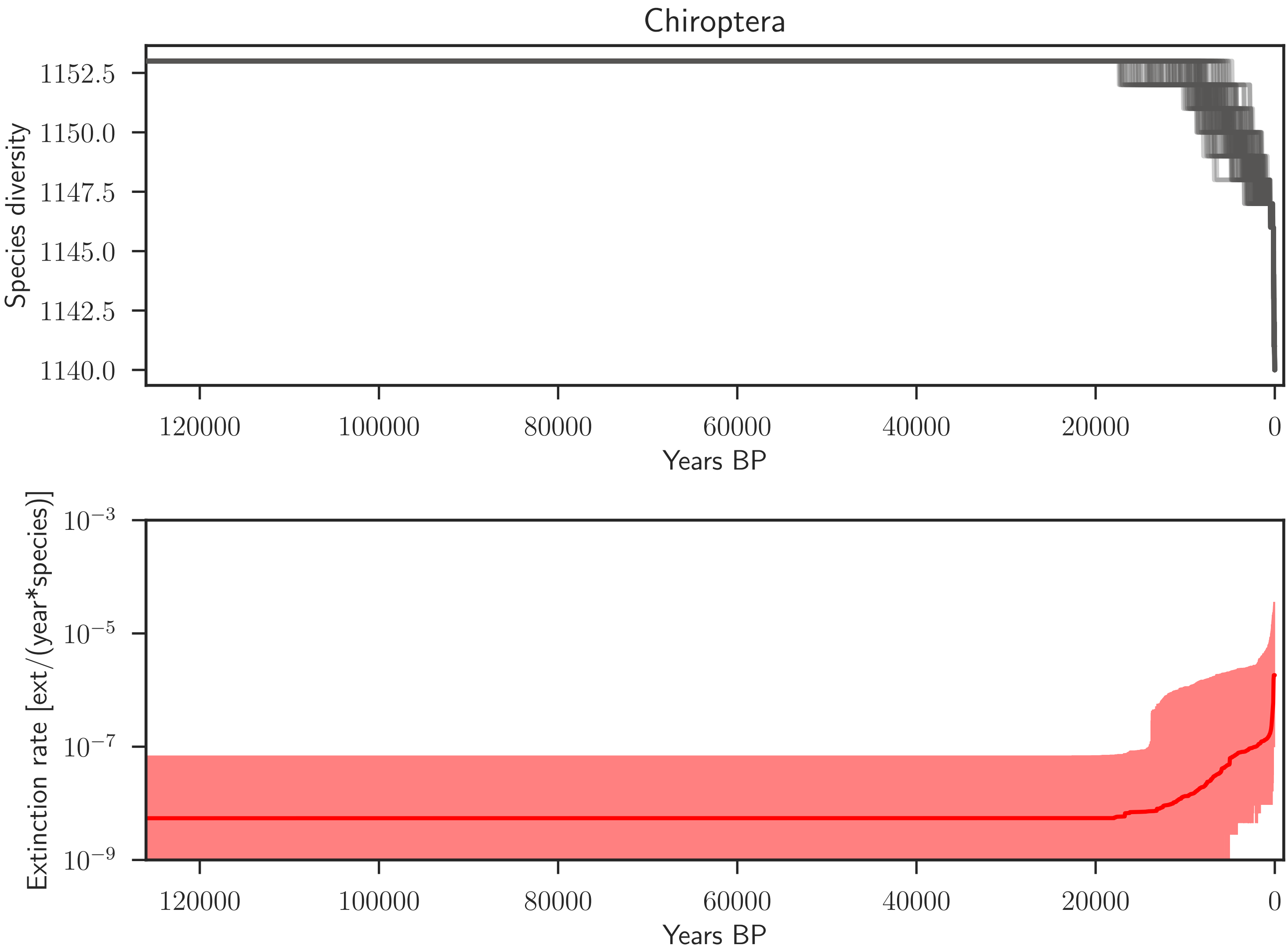
Chiroptera
Species diversity
1152.5
1150.0
1147.5
1145.0
1142.5
1140.0
120000
100000
80000
60000
40000
20000
0
Years BP
Extinction rate [ext/(year*species)]
10^{-3}
10^{-5}
10^{-7}
10^{-9}
120000
100000
80000
60000
40000
20000
0
Years BP

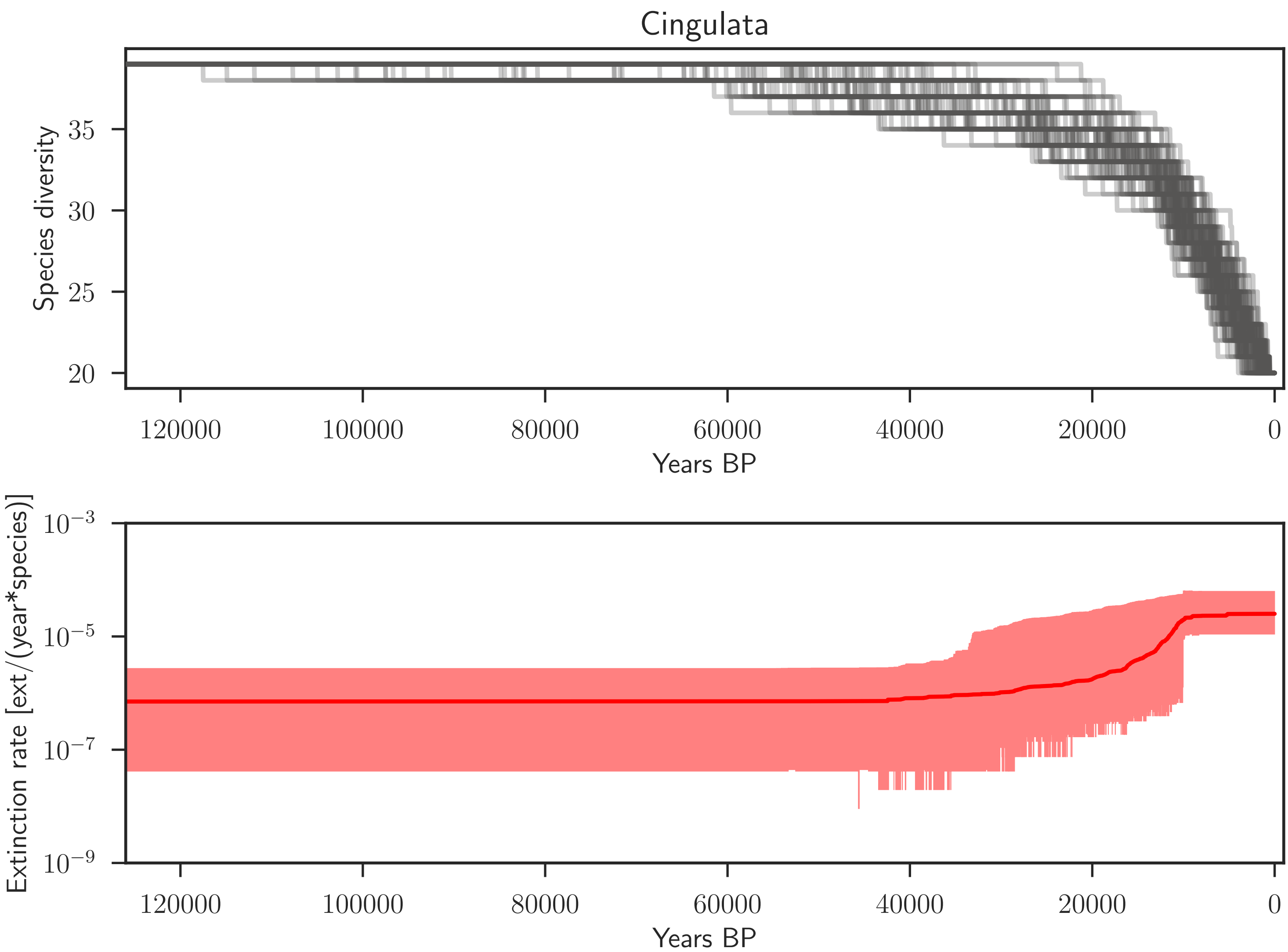
Cingulata
Species diversity
20
25
30
35
120000
100000
80000
60000
40000
20000
0
Years BP
Extinction rate [ext/(year*species)]
10^{-3}
10^{-5}
10^{-7}
10^{-9}
120000
100000
80000
60000
40000
20000
0
Years BP

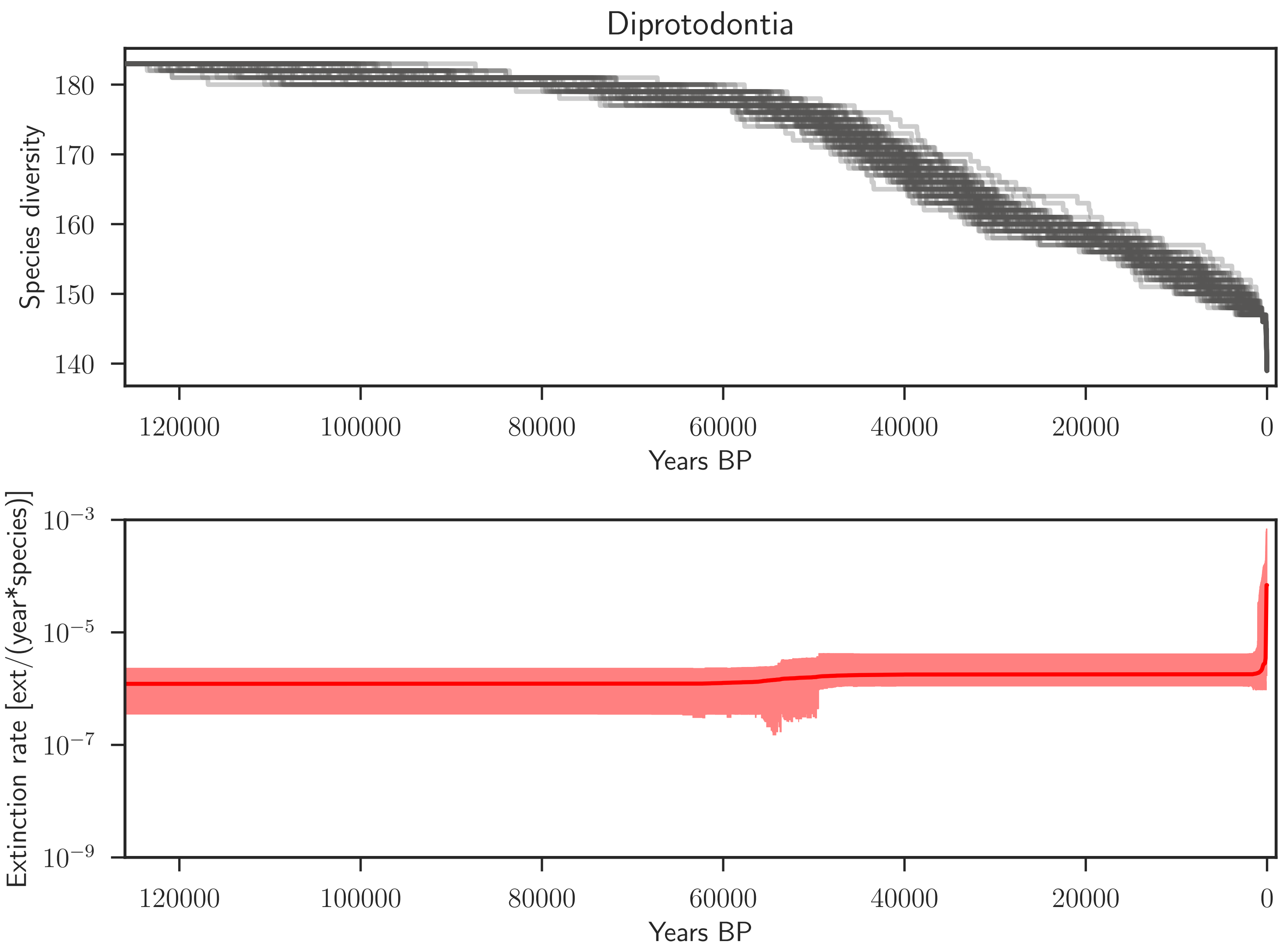

Diprotodontia
Species diversity
180
170
160
150
140
120000
100000
80000
60000
40000
20000
0
Years BP
Extinction rate [ext/(year*species)]
10^{-3}
10^{-5}
10^{-7}
10^{-9}
120000
100000
80000
60000
40000
20000
0
Years BP

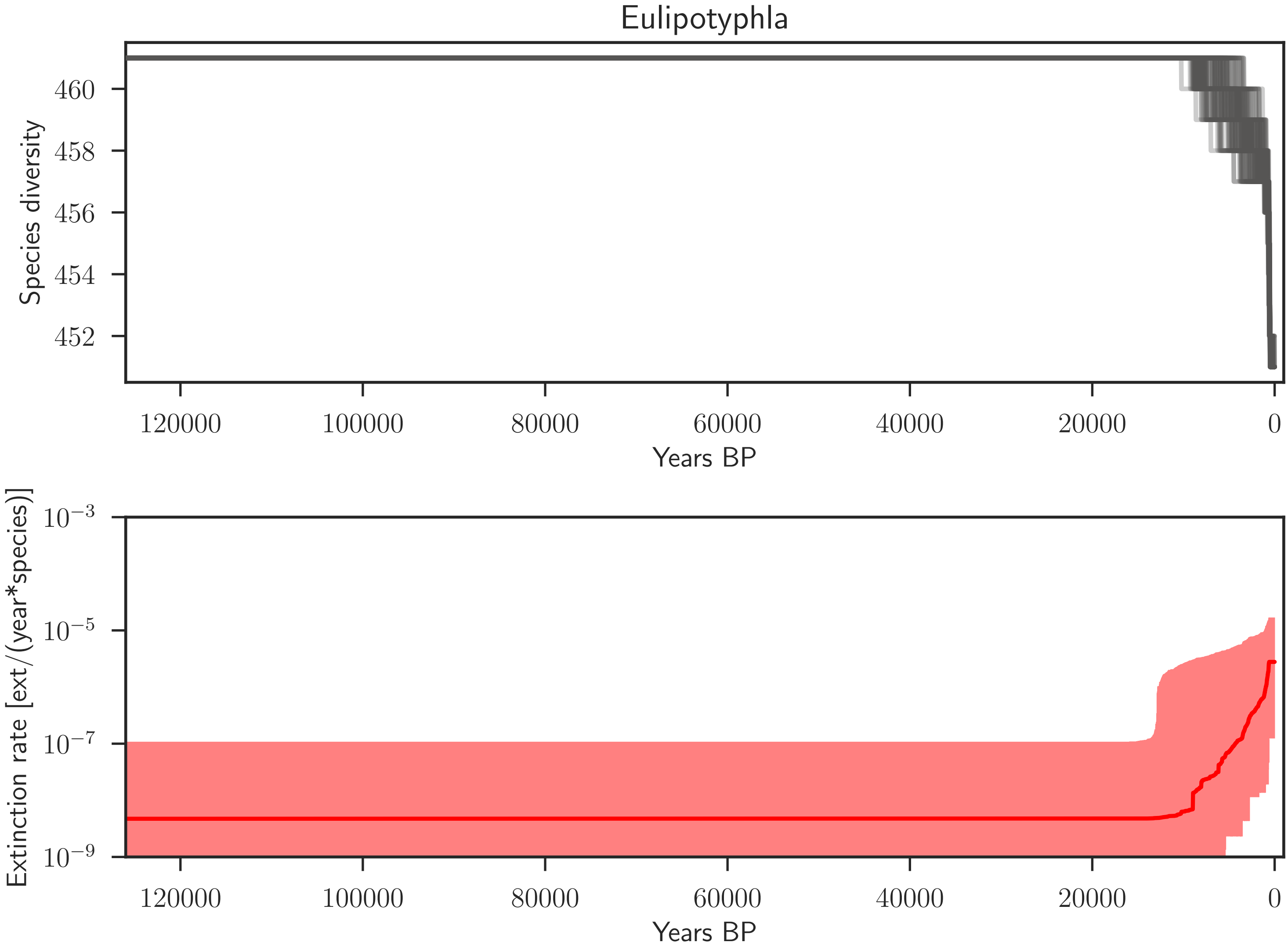

Eulipotyphla
Species diversity
460
458
456
454
452
120000
100000
80000
60000
40000
20000
0
Years BP
Extinction rate [ext/(year*species)]
10^{-3}
10^{-5}
10^{-7}
10^{-9}
120000
100000
80000
60000
40000
20000
0
Years BP

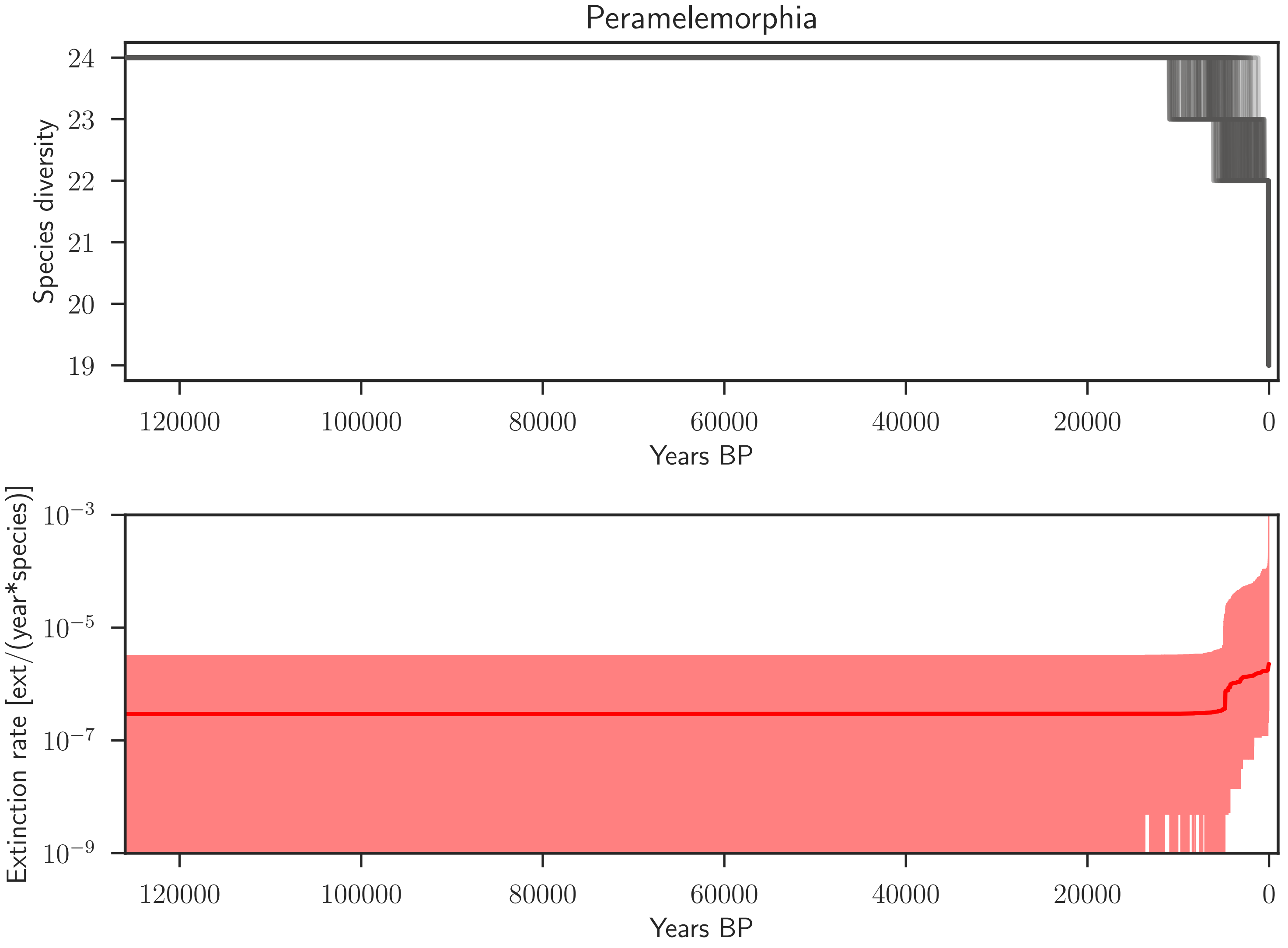

Peramelemorphia
Species diversity
24
23
22
21
20
19
120000
100000
80000
60000
40000
20000
0
Years BP
Extinction rate [ext/(year*species)]
10^{-3}
10^{-5}
10^{-7}
10^{-9}
120000
100000
80000
60000
40000
20000
0
Years BP

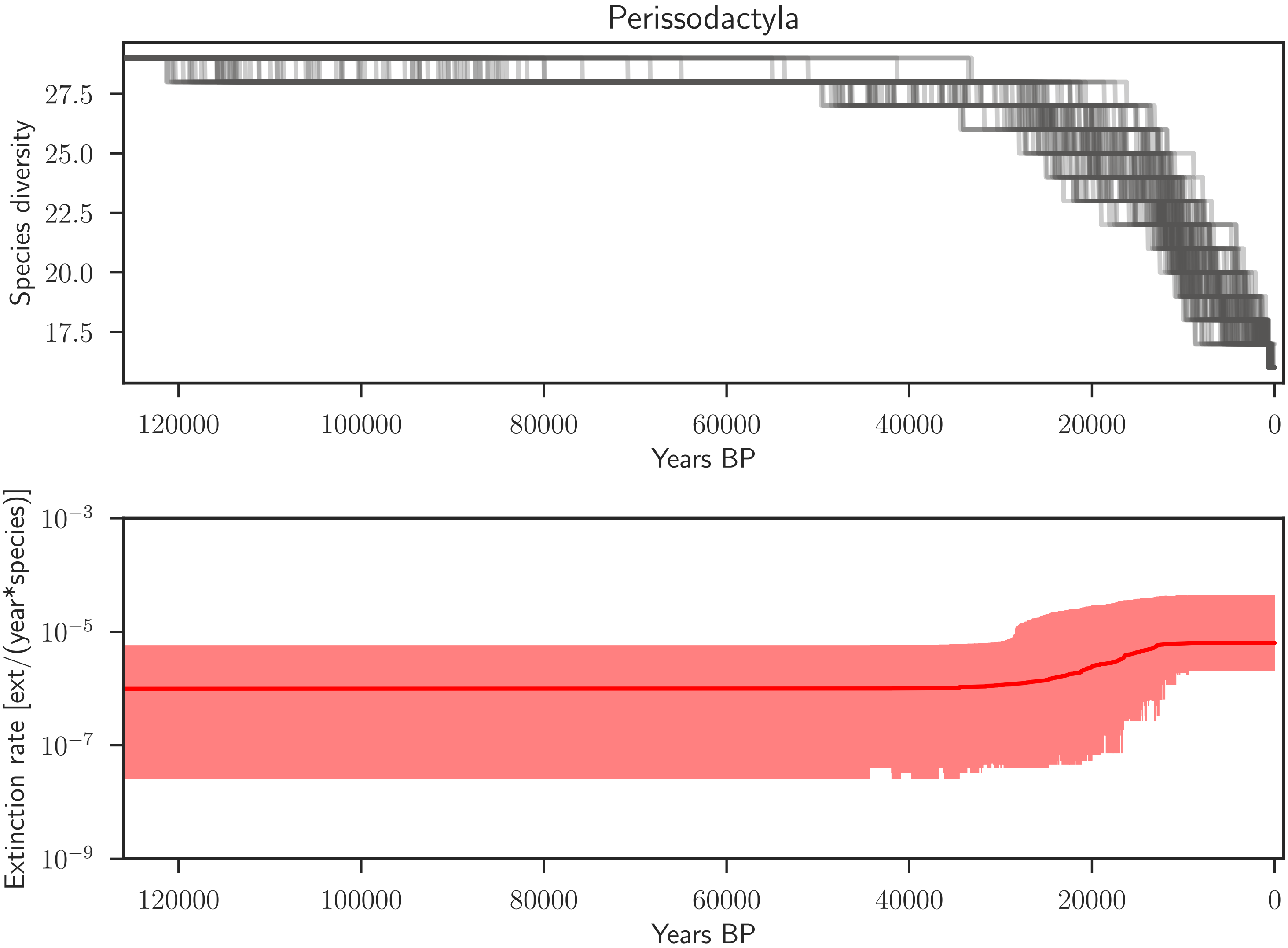

Perissodactyla
Species diversity
27.5
25.0
22.5
20.0
17.5
120000
100000
80000
60000
40000
20000
0
Years BP
Extinction rate [ext/(year*species)]
10^{-3}
10^{-5}
10^{-7}
10^{-9}
120000
100000
80000
60000
40000
20000
0
Years BP

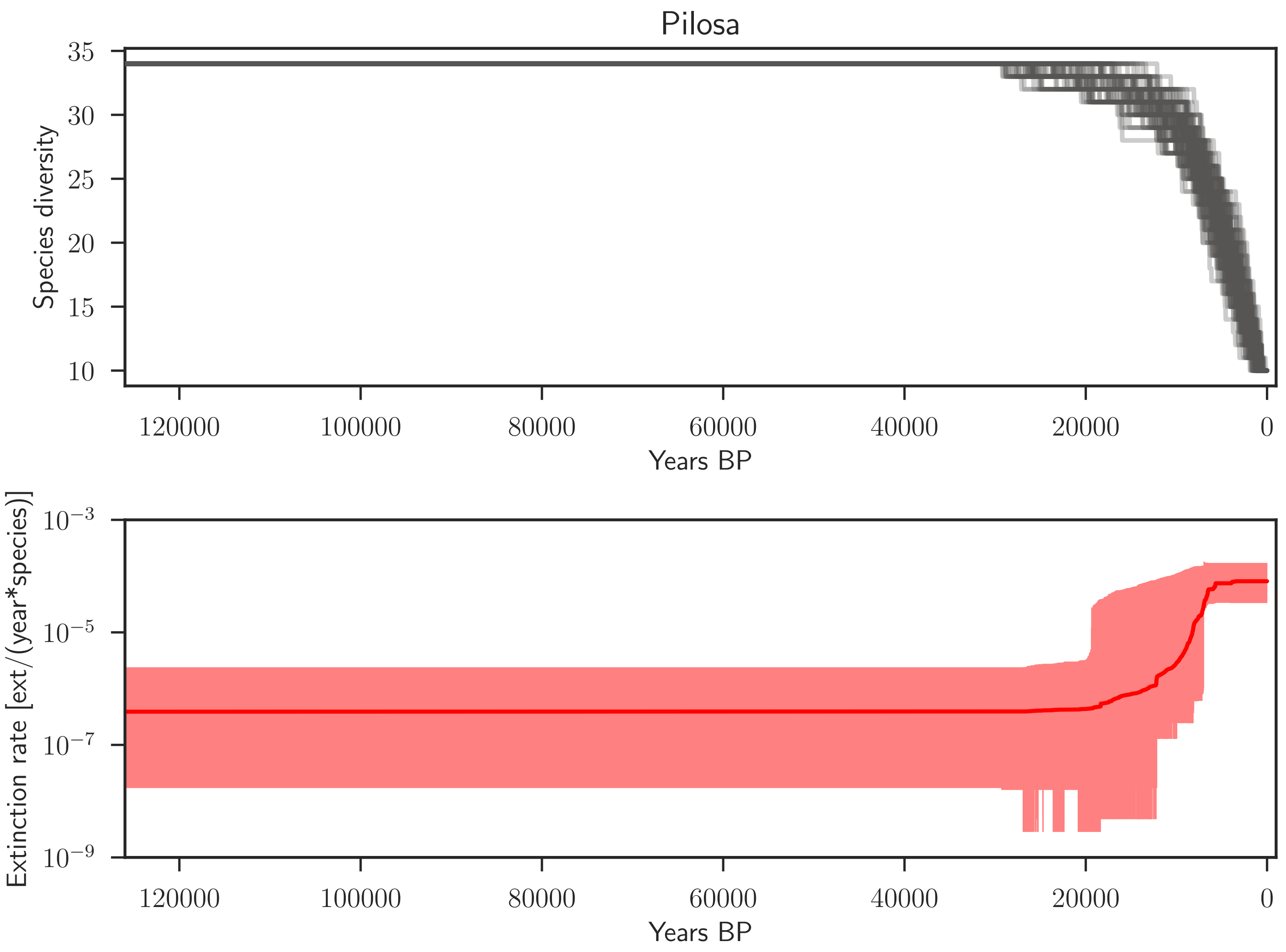

Pilosa
Species diversity
35
30
25
20
15
10
120000
100000
80000
60000
40000
20000
0
Years BP
Extinction rate [ext/(year*species)]
10^{-3}
10^{-5}
10^{-7}
10^{-9}
120000
100000
80000
60000
40000
20000
0
Years BP

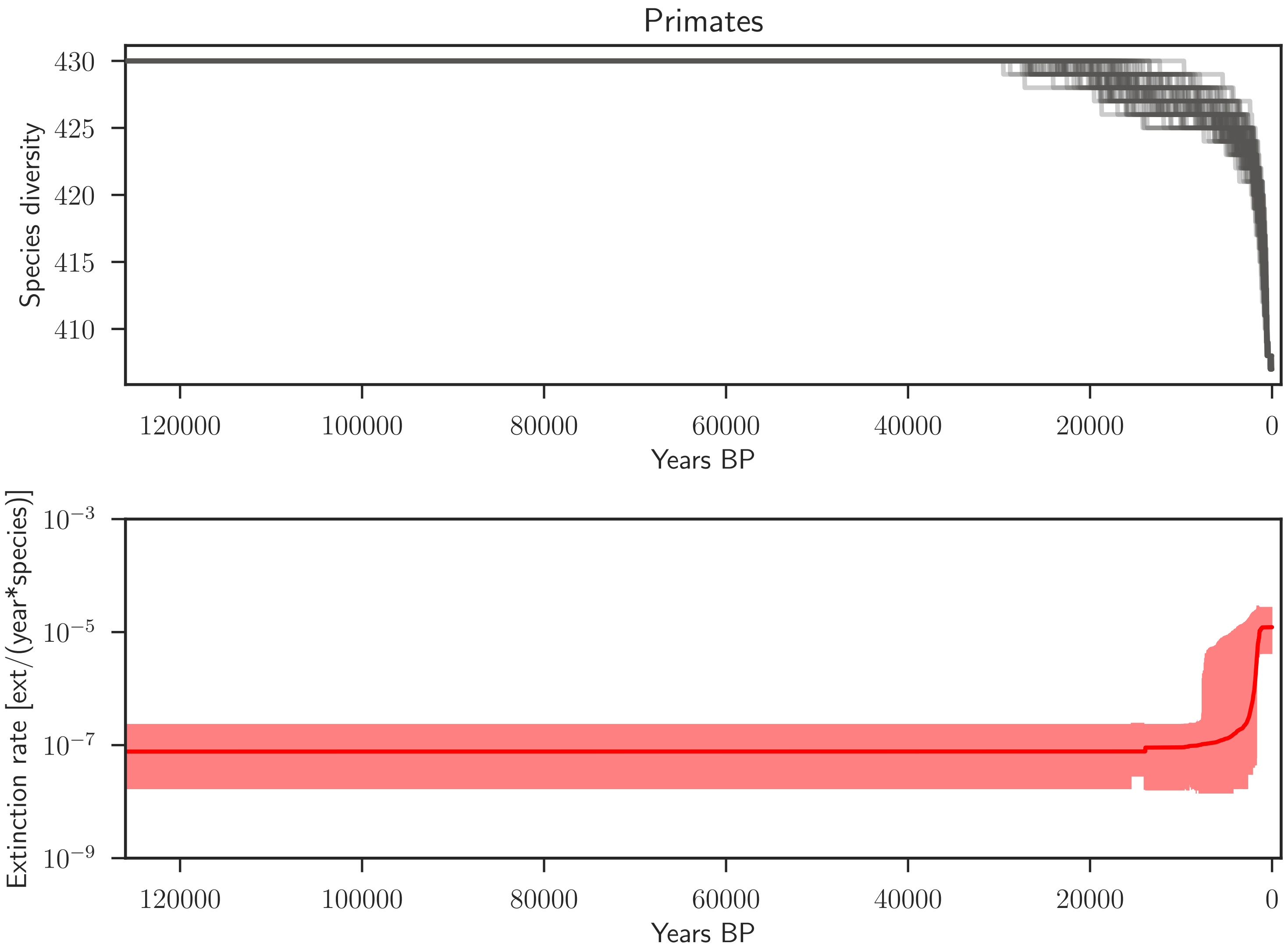

Primates
Species diversity
430
425
420
415
410
120000
100000
80000
60000
40000
20000
0
Years BP
Extinction rate [ext/(year*species)]
10^{-3}
10^{-5}
10^{-7}
10^{-9}
120000
100000
80000
60000
40000
20000
0
Years BP

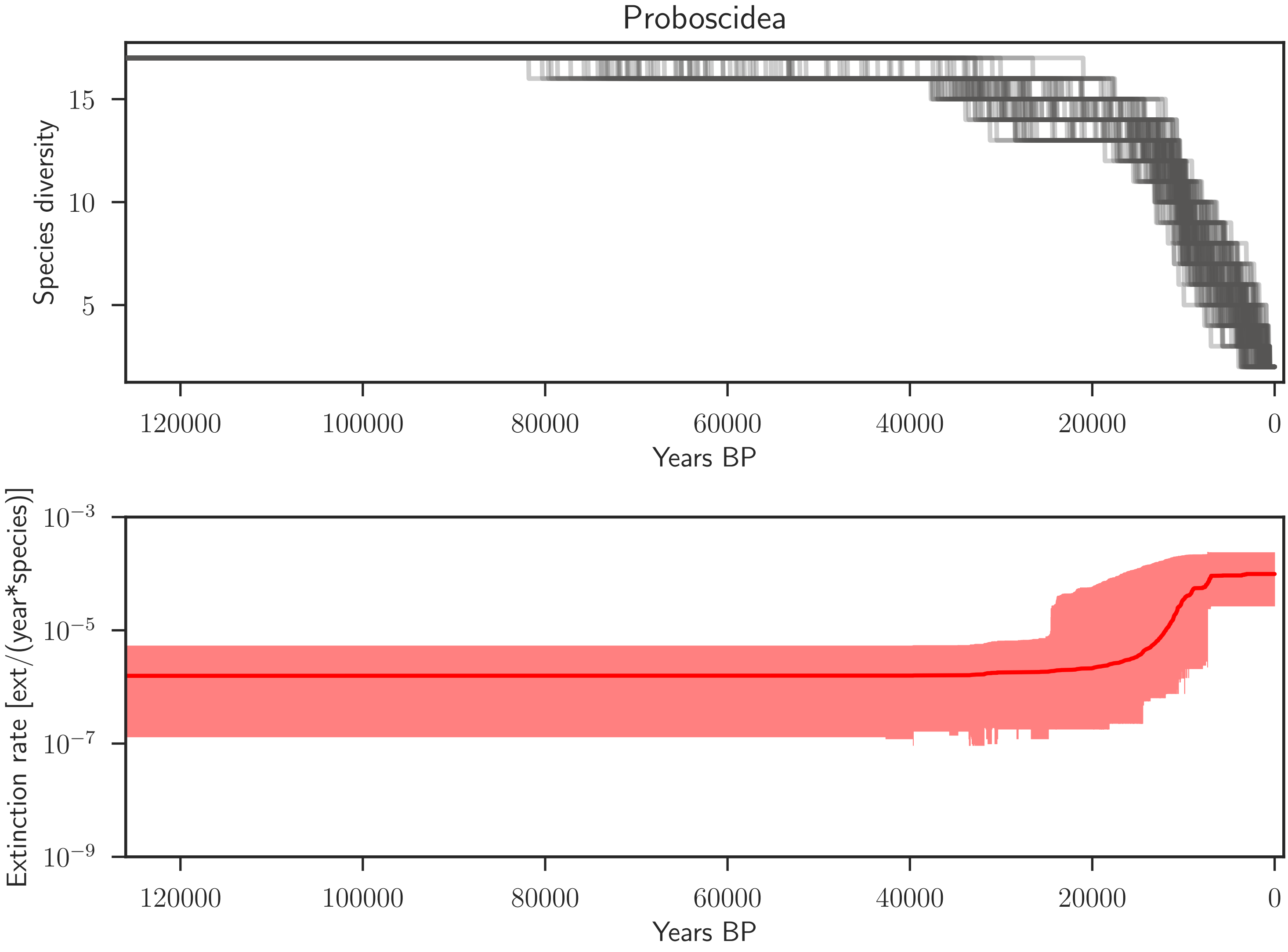
Proboscidea
Species diversity
15
10
5
120000
100000
80000
60000
40000
20000
0
Years BP
Extinction rate [ext/(year*species)]
10^{-3}
10^{-5}
10^{-7}
10^{-9}
120000
100000
80000
60000
40000
20000
0
Years BP

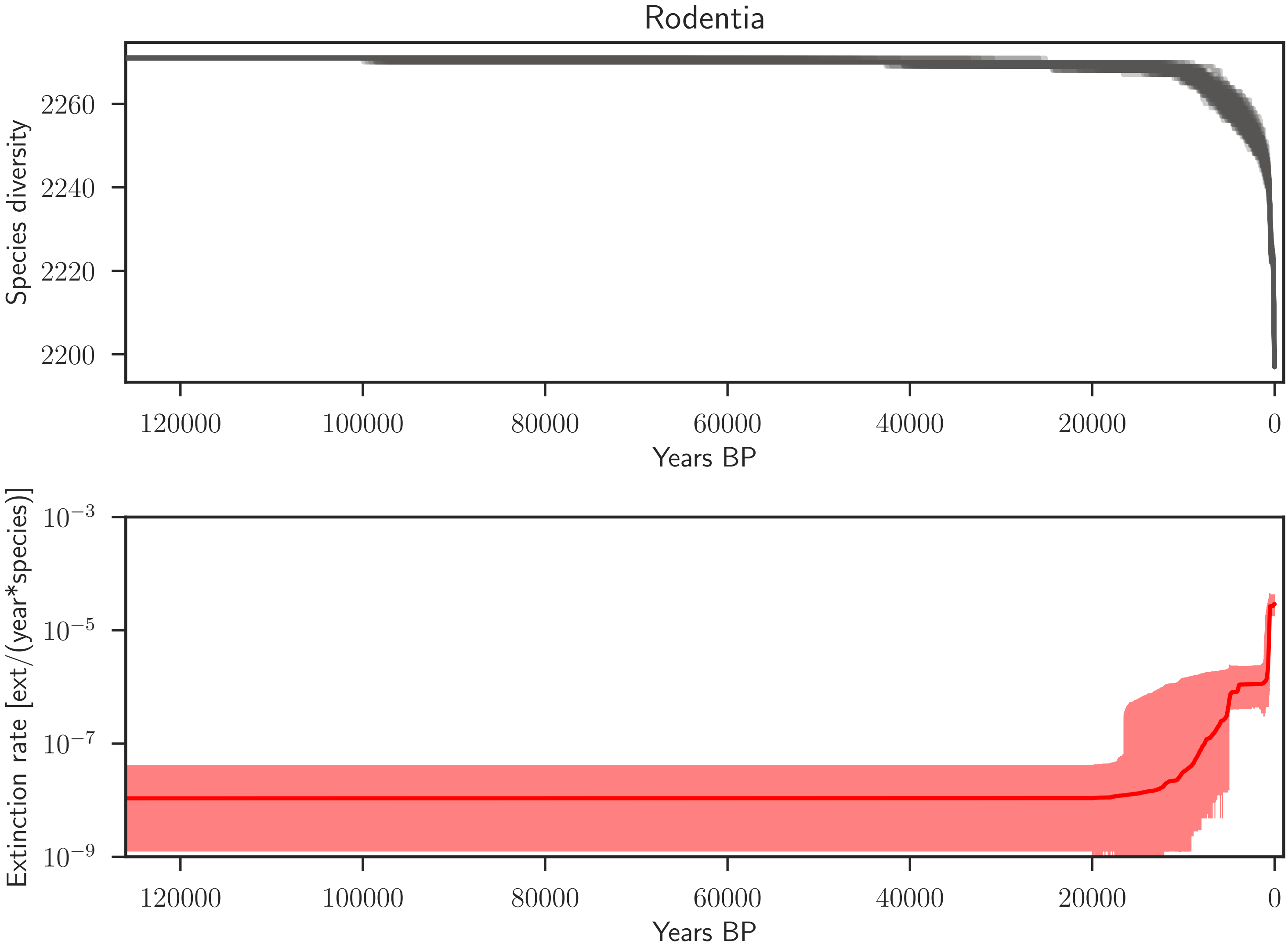
Rodentia
Species diversity
2260
2240
2220
2200
120000
100000
80000
60000
40000
20000
0
Years BP
Extinction rate [ext/(year*species)]
10^{-3}
10^{-5}
10^{-7}
10^{-9}
120000
100000
80000
60000
40000
20000
0
Years BP
