# Supplementary material for: The past and future human impact on mammalian diversity
Source: Sci Adv. 2020 Sep 4;6(36):eabb2313. doi: 10.1126/sciadv.abb2313 (PMC7473673; doi:10.1126/sciadv.abb2313)
Supplement: abb2313_Data_file_S2.pdf [file abb2313_Data_file_S2.pdf]

**Species**	$\mathbf{LO_{max}}$	$\mathbf{LO_{min}}$	**Ref.**	**N**	$\mathbf{FO_{DB}}$	$\mathbf{LO_{DB}}$	**q**
Acratocnus odontrigonus	3449	3349	63	0	NaN	NaN	*0.000044*
Acratocnus ye	8405	7973	64	0	NaN	NaN	*0.000044*
Agalmaceros blicki	10069	7069	65	0	NaN	NaN	*0.000044*
Alces scotti	9199	6661	38	0	NaN	NaN	*0.000044*
Amblyrhiza inundata	100069	100069	66	0	NaN	NaN	*0.000044*
Antidorcas australis	7069	7069	67	0	NaN	NaN	*0.000044*
Antidorcas bondi	8626	8249	63	0	NaN	NaN	*0.000044*
Antifer ultra	20069	10069	6	0	NaN	NaN	*0.000044*
Antillomys rayi	11769	519	63	0	NaN	NaN	*0.000044*
Antillothrix bernensis	4858	3941	31	0	NaN	NaN	*0.000044*
Archaeoindris fontoynonti	2431	2218	63	0	NaN	NaN	*0.000044*
Archaeolemur edwardsi	2351	1909	63	1	5000	5000	*0.000069*
Archaeolemur majori	1649	1444	63	2	5000	5000	*0.000091*
Arctodus simus	9759	9639	68	25	46700	9600	0.000647
Arctotherium tarijense	11329	11229	68	0	NaN	NaN	*0.000044*
Arctotherium wingei	12069	12069	68	0	NaN	NaN	*0.000044*
Artibeus anthonyi	9062	8522	31	0	NaN	NaN	*0.000044*
Aztlanolagus agilis	36069	25069	69	0	NaN	NaN	*0.000044*
Babakotia radofilai	5359	4909	31	0	NaN	NaN	*0.000044*
Bettongia anhydra	86	NaN	1	0	NaN	NaN	*0.000044*
Bettongia pusilla	519	NaN	63	0	NaN	NaN	*0.000044*
Bootherium bombifrons	15069	11469	38	50	40000	5250	0.001410
Boromys offella	9062	8522	31	0	NaN	NaN	*0.000044*
Boromys torrei	9062	8522	31	0	NaN	NaN	*0.000044*
Borungaboodie hatcheri	123069	101069	39	2	136800	119500	0.000058
Bos primigenius	392	392	1	1	26500	26500	*0.000069*
Brachyprotoma obtusata	13809	13809	38	3	14215	5690	0.000235
Brotomys voratus	609	379	31	0	NaN	NaN	*0.000044*
Bubalus grovesi	6069	2069	70	0	NaN	NaN	*0.000044*

Caipora bambuiorum	20419	19839	71	0	NaN	NaN	*0.000044*
Caloprymnus campestris	39	NaN	63	0	NaN	NaN	*0.000044*
Camelops hesternus	11909	10379	38	38	37900	2275	0.001039
Camelus dromedarius	2969	969	72	0	NaN	NaN	*0.000044*
Canariomys bravoi	2428	2224	63	0	NaN	NaN	*0.000044*
Canariomys tamarani	11769	519	63	0	NaN	NaN	*0.000044*
Candiacervus sp.	8069	8069	73	0	NaN	NaN	*0.000044*
Candiacervus cretensis	8069	8069	73	0	NaN	NaN	*0.000044*
Candiacervus dorothensis	8069	8069	73	0	NaN	NaN	*0.000044*
Candiacervus major	8069	8069	73	0	NaN	NaN	*0.000044*
Candiacervus rethymnensis	8069	8069	73	0	NaN	NaN	*0.000044*
Candiacervus ropalophorus	8069	8069	73	0	NaN	NaN	*0.000044*
Canis dirus	9519	3569	38	38	32500	1230	0.001183
Capreolus miyakoensis	18069	15069	74	0	NaN	NaN	*0.000044*
Caprini indet	6379	6299	75	0	NaN	NaN	*0.000044*
Capromeryx minor	12569	10069	38	5	30500	12000	0.000216
Castoroides ohioensis	11099	9639	38	17	54000	217	0.000298
Catagonus stenocephalus	40069	28069	76	0	NaN	NaN	*0.000044*
Catonyx cuvieri	9849	9449	63	0	NaN	NaN	*0.000044*
Cervus astylodon	35069	20069	74	0	NaN	NaN	*0.000044*
Chaeropus ecaudatus	69	NaN	63	4	205000	435	0.000015
Coelodonta antiquitatis	14069	14069	77	3	43000	26500	0.000121
Conilurus albipes	174	NaN	63	12	55200	1290	0.000204
Conilurus capricornensis	519	NaN	1	0	NaN	NaN	*0.000044*
Coryphomys buehleri	519	NaN	63	0	NaN	NaN	*0.000044*
Coryphomys musseri	11769	519	63	0	NaN	NaN	*0.000044*
Cryptonanus ignitus	57	NaN	63	0	NaN	NaN	*0.000044*
Cryptoprocta spelea	361	NaN	63	1	5000	5000	*0.000069*
Cuscomys oblativa	569	NaN	1	0	NaN	NaN	*0.000044*
Cuvieronius hyodon	14643	13458	78	0	NaN	NaN	*0.000044*
Cynotherium sardous	11519	11319	63	0	NaN	NaN	*0.000044*

Dactylopsila kambuayai	7960	7615	63	0	NaN	NaN	*0.000044*
Damaliscus hypsodon	46880	32544	79	0	NaN	NaN	*0.000044*
Damaliscus niro	64269	61669	75	0	NaN	NaN	*0.000044*
Dasypus bellus	11249	10889	38	19	23240	694	0.000798
Daubentonia robustus	1128	992	63	0	NaN	NaN	*0.000044*
Desmodus draculae	537	224	63	0	NaN	NaN	*0.000044*
Desmodus puntajudensis	9062	8522	31	0	NaN	NaN	*0.000044*
Desmodus stocki	10769	10769	63	0	NaN	NaN	*0.000044*
Diabolotherium nordenskioldi	29469	28949	70	0	NaN	NaN	*0.000044*
Dicroceros sp	35069	20069	74	0	NaN	NaN	*0.000044*
Diprotodon optatum	52069	40069	39	18	58000	14950	0.000395
Doedicurus clavicaudatus	6784	6464	63	0	NaN	NaN	*0.000044*
Dusicyon australis	143	NaN	63	0	NaN	NaN	*0.000044*
Dusicyon avus	500	328	80	0	NaN	NaN	*0.000044*
Elaphurus davidianus	23	NaN	1	0	NaN	NaN	*0.000044*
Elasmotherium sibiricum	50069	50069	81	0	NaN	NaN	*0.000044*
Elephas antiquus	37859	37199	82	0	NaN	NaN	*0.000044*
Elephas cypriotes	12718	9318	63	0	NaN	NaN	*0.000044*
Elephas iolensis	34069	34069	83	0	NaN	NaN	*0.000044*
Elephas mnaidriensis	36069	28069	84	0	NaN	NaN	*0.000044*
Elephas namadicus	16069	16069	85	0	NaN	NaN	*0.000044*
Elephas naumanii	16229	15469	86	0	NaN	NaN	*0.000044*
Elephas tiliensis	11769	519	63	0	NaN	NaN	*0.000044*
Eliomys morpheus	6858	6708	63	0	NaN	NaN	*0.000044*
Equus francisci	13194	13084	87	1	13290	13290	*0.000069*
Equus hydruntinus	726	NaN	63	0	NaN	NaN	*0.000044*
Equus ovodovi	26019	25549	81	0	NaN	NaN	*0.000044*
Eremotherium laurillardi	13371	13185	78	0	NaN	NaN	*0.000044*
Euceratherium collinum	11369	169	38	4	13225	10000	0.000930
Eutatus seguini	10529	10019	88	0	NaN	NaN	*0.000044*
Gazella atlantica	13569	13569	89	0	NaN	NaN	*0.000044*

Gazella bilkis	68	NaN	1	0	NaN	NaN	*0.000044*
Gazella saudiya	39	NaN	1	0	NaN	NaN	*0.000044*
Gazella tingitana	41069	33069	75	0	NaN	NaN	*0.000044*
Geocapromys sp.	580	376	63	0	NaN	NaN	*0.000044*
Geocapromys columbianus	9062	8522	31	0	NaN	NaN	*0.000044*
Geocapromys thoracatus	69	NaN	1	0	NaN	NaN	*0.000044*
Glossotherium robustum	8829	8669	63	0	NaN	NaN	*0.000044*
Glyptodon clavipes	8679	6899	63	2	26740	15500	0.000089
Glyptodon reticulatus	126069	8269	90	0	NaN	NaN	*0.000044*
Glyptotherium cylindricum	13589	12499	91	0	NaN	NaN	*0.000044*
Glyptotherium floridanum	23299	10069	38	1	23230	23230	*0.000069*
Hadropithecus stenognathus	1575	1247	31	1	5000	5000	*0.000069*
Haploidoceros mediterraneus	100069	86069	92	0	NaN	NaN	*0.000044*
Hemiauchenia macrocephala	13069	9069	38	19	620000	11000	0.000030
Hemiauchenia paradoxa	12709	11849	93	0	NaN	NaN	*0.000044*
Hemitragus cedrensis	139069	103069	94	0	NaN	NaN	*0.000044*
Heteropsomys insulans	1325	1132	63	0	NaN	NaN	*0.000044*
Hexolobodon phenax	519	NaN	63	0	NaN	NaN	*0.000044*
Hexolobodontinae sp.	4854	3710	63	0	NaN	NaN	*0.000044*
Hippidion devillei	23579	23059	95	0	NaN	NaN	*0.000044*
Hippidion principale	12709	12639	96	0	NaN	NaN	*0.000044*
Hippopotamus lemerlei	1864	1604	31	2	5000	5000	*0.000091*
Hippopotamus madagascariensis	380	380	63	0	NaN	NaN	*0.000044*
Hippotragus leucophaeus	219	NaN	63	0	NaN	NaN	*0.000044*
Holmesina occidentalis	28419	24969	97	0	NaN	NaN	*0.000044*
Holmesina paulacoutoi	27579	27179	98	0	NaN	NaN	*0.000044*
Holmesina septentrionalis	14069	10069	38	6	15000	9840	0.000969
Homo denisovans	30379	15744	99	0	NaN	NaN	*0.000044*
Homo floresiensis	18769	17469	100	0	NaN	NaN	*0.000044*
Homo neanderthalensis	28069	28069	101	0	NaN	NaN	*0.000044*
Homotherium latidens	27009	26529	102	0	NaN	NaN	*0.000044*

Homotherium serum	22449	22189	103	4	620000	13969	0.000005
Hoplophorus euphractus	8679	6899	63	0	NaN	NaN	*0.000044*
Hydrodamalis gigas	251	NaN	63	0	NaN	NaN	*0.000044*
Hypogeomys australis	1591	1401	31	1	5000	5000	*0.000069*
Hystrix kiangsenensis	30569	4739	104	0	NaN	NaN	*0.000044*
Hystrix refossa	43769	40369	105	0	NaN	NaN	*0.000044*
Insulacebus toussaintiana	9769	3784	106	0	NaN	NaN	*0.000044*
Isolobodon montanus	519	NaN	63	0	NaN	NaN	*0.000044*
Isolobodon portoricensis	739	594	63	0	NaN	NaN	*0.000044*
Juscelinomys candango	59	NaN	63	0	NaN	NaN	*0.000044*
Kolpochoerus majus	29339	28139	75	0	NaN	NaN	*0.000044*
Lagorchestes asomatus	59	NaN	63	0	NaN	NaN	*0.000044*
Lagorchestes leporides	129	NaN	63	18	35000	1000	0.000500
Lagostomus crassus	109	NaN	63	0	NaN	NaN	*0.000044*
Leopardus amnicola	9379	8069	38	0	NaN	NaN	*0.000044*
Leporillus apicalis	86	NaN	1	5	4440	390	0.000988
Lestodon armatus	20069	10069	6	0	NaN	NaN	*0.000044*
Macrauchenia patachonica	10009	9909	107	0	NaN	NaN	*0.000044*
Macropus ferragus	60069	44069	39	3	26299	14300	0.000167
Macropus greyi	80	NaN	63	8	72300	10940	0.000114
Macropus pearsoni	132069	92069	39	5	36000	19500	0.000242
Macrotis leucura	69	NaN	63	0	NaN	NaN	*0.000044*
Malpaisomys insularis	1235	903	63	0	NaN	NaN	*0.000044*
Mammut americanum	10439	10099	108	101	90000	850	0.001122
Mammuthus columbi	11569	9569	109	61	35250	4744	0.001967
Mammuthus exilis	13319	12499	110	2	12840	11750	0.000917
Mammuthus primigenius	4210	4056	63	33	48100	8280	0.000804
Manis paleojavanica	47069	42069	111	0	NaN	NaN	*0.000044*
Maokopia ronaldi	18069	14069	112	2	42740	16000	0.000037
Megaceroides algericus	20691	19870	113	0	NaN	NaN	*0.000044*
Megaladapis edwardsi	739	599	31	2	5000	5000	*0.000091*

Megaladapis grandidieri	1119	979	31	0	NaN	NaN	*0.000044*
Megaladapis madagascariensis	2389	2059	31	2	5000	5000	*0.000091*
Megalibgwilia ramsayi	44069	40469	39	8	136800	9999	0.000055
Megaloceros giganteus	7794	7654	63	1	32000	32000	*0.000069*
Megalocnus rodens	4909	4649	31	0	NaN	NaN	*0.000044*
Megalocnus zile	18374	16574	64	0	NaN	NaN	*0.000044*
Megalomys audreyae	846	634	63	0	NaN	NaN	*0.000044*
Megalomys desmarestii	122	NaN	63	0	NaN	NaN	*0.000044*
Megalomys georginae	819	619	114	0	NaN	NaN	*0.000044*
Megalomys luciae	138	NaN	63	0	NaN	NaN	*0.000044*
Megalonyx jeffersonii	10299	169	38	11	30100	9400	0.000483
Megalotragus priscus	9147	8673	63	0	NaN	NaN	*0.000044*
Megalovis guangxiensis	132069	109069	115	0	NaN	NaN	*0.000044*
Megaoryzomys sp.	504	504	63	0	NaN	NaN	*0.000044*
Megaoryzomys curioi	499	499	63	0	NaN	NaN	*0.000044*
Megatherium americanum	7439	7339	63	0	NaN	NaN	*0.000044*
Megatherium tarijense	28129	26369	116	0	NaN	NaN	*0.000044*
Melomys rubicola	10	NaN	1	0	NaN	NaN	*0.000044*
Mesocricetus rathgeberi	11769	519	63	0	NaN	NaN	*0.000044*
Mesopropithecus globiceps	1774	1590	31	0	NaN	NaN	*0.000044*
Mesopropithecus pithecoides	1449	1340	31	0	NaN	NaN	*0.000044*
Metasthenurus newtonae	60069	52069	39	5	136800	7880	0.000031
Metridiochoerus compactus	27519	23919	117	0	NaN	NaN	*0.000044*
Microgale macpheei	2808	2428	31	1	5000	5000	*0.000069*
Microtus henseli	795	560	63	0	NaN	NaN	*0.000044*
Miracinonyx trumani	12165	10069	38	3	19750	13544	0.000322
Mixotoxodon larensis	20069	10069	6	0	NaN	NaN	*0.000044*
Morenelaphus brachyceros	65769	39969	118	0	NaN	NaN	*0.000044*
Morenelaphus lujanensis	65769	39969	118	0	NaN	NaN	*0.000044*
Mormoops magna	9062	8522	31	0	NaN	NaN	*0.000044*
Muknalia minima	9208	8909	119	0	NaN	NaN	*0.000044*

Mylodon darwinii	10669	9869	120	0	NaN	NaN	*0.000044*
Mylohyus nasutus	11969	11889	93	12	15000	5900	0.001209
Myotragus balearicus	6738	6418	63	0	NaN	NaN	*0.000044*
Navahoceros fricki	11369	10969	38	8	620000	11099	0.000012
Neochoerus aesopi	20069	10069	38	0	NaN	NaN	*0.000044*
Neocnus comes	5329	4909	31	0	NaN	NaN	*0.000044*
Neocnus dousman	7245	7175	31	0	NaN	NaN	*0.000044*
Neocnus toupiti	21129	18929	64	0	NaN	NaN	*0.000044*
Neolicaphrium recens	65769	39969	118	0	NaN	NaN	*0.000044*
Neomonachus tropicalis	67	NaN	1	0	NaN	NaN	*0.000044*
Neosclerocalyptus paskoenis	9769	6769	121	0	NaN	NaN	*0.000044*
Neotoma anthonyi	93	NaN	63	0	NaN	NaN	*0.000044*
Neotoma bunkeri	88	NaN	63	0	NaN	NaN	*0.000044*
Neotoma martinensis	69	NaN	63	0	NaN	NaN	*0.000044*
Neovison macrodon	125	NaN	63	0	NaN	NaN	*0.000044*
Nesiotites hidalgo	5048	4708	63	0	NaN	NaN	*0.000044*
Nesiotites similis	9569	8809	122	0	NaN	NaN	*0.000044*
Nesophontes sp.	11769	519	63	0	NaN	NaN	*0.000044*
Nesophontes edithae	1222	1060	63	0	NaN	NaN	*0.000044*
Nesophontes hypomicrus	844	724	31	0	NaN	NaN	*0.000044*
Nesophontes major	9062	8522	63	0	NaN	NaN	*0.000044*
Nesophontes micrus	724	589	31	0	NaN	NaN	*0.000044*
Nesophontes paramicrus	754	619	31	0	NaN	NaN	*0.000044*
Nesophontes zamicrus	724	589	31	0	NaN	NaN	*0.000044*
Nesoryzomys sp.A	11769	519	63	0	NaN	NaN	*0.000044*
Nesoryzomys sp.B	11769	519	63	0	NaN	NaN	*0.000044*
Nesoryzomys sp.C	6790	6385	63	0	NaN	NaN	*0.000044*
Nesoryzomys darwini	79	NaN	63	0	NaN	NaN	*0.000044*
Nesoryzomys indefessus	85	NaN	63	0	NaN	NaN	*0.000044*
Neuryurus trabeculatus	65769	39969	118	0	NaN	NaN	*0.000044*
Noronhomys vespuccii	516	NaN	63	0	NaN	NaN	*0.000044*

Nothrotheriops shastense	10854	9354	38	0	NaN	NaN	*0.000044*
Nothrotherium maquinense	12389	12149	120	0	NaN	NaN	*0.000044*
Notiomastodon platensis	18878	18630	78	0	NaN	NaN	*0.000044*
Notomys amplus	123	NaN	63	0	NaN	NaN	*0.000044*
Notomys longicaudatus	118	NaN	63	0	NaN	NaN	*0.000044*
Notomys macrotis	176	NaN	63	0	NaN	NaN	*0.000044*
Notomys mordax	179	NaN	63	0	NaN	NaN	*0.000044*
Notomys robustus	519	NaN	1	0	NaN	NaN	*0.000044*
Ochotona whartoni	10320	9209	63	0	NaN	NaN	*0.000044*
Oligoryzomys victus	127	NaN	63	0	NaN	NaN	*0.000044*
Onychogalea lunata	63	NaN	63	10	13000	390	0.000714
Oreamnos harringtoni	11539	8379	38	27	28749	9765	0.001370
Oryx dammah	19	NaN	1	0	NaN	NaN	*0.000044*
Oryzomys antillarum	142	NaN	63	0	NaN	NaN	*0.000044*
Oryzomys nelsoni	122	NaN	63	0	NaN	NaN	*0.000044*
Pachyarmaterium brasiliense	22069	18069	123	0	NaN	NaN	*0.000044*
Pachylemur insignis	1304	1034	31	1	5000	5000	*0.000069*
Pachylemur jullyi	8529	1054	124	0	NaN	NaN	*0.000044*
Palaeolama major	8679	6899	63	0	NaN	NaN	*0.000044*
Palaeolama mirifica	11089	10829	93	3	37000	15000	*0.000091*
Palaeolama weddeli	21009	20809	116	0	NaN	NaN	*0.000044*
Palaeopropithecus ingens	719	399	63	1	5000	5000	*0.000069*
Palaeopropithecus maximus	2421	2226	125	0	NaN	NaN	*0.000044*
Palorchestes azeal	29069	25069	112	0	NaN	NaN	*0.000044*
Pampatherium humboldti	65769	39969	118	0	NaN	NaN	*0.000044*
Pampatherium typum	21069	10069	126	0	NaN	NaN	*0.000044*
Panochthus tuberculatus	65769	39969	118	0	NaN	NaN	*0.000044*
Panthera atrox	11479	11369	127	25	48100	8685	0.000609
Panthera spelaea	12064	11924	127	0	NaN	NaN	*0.000044*
Papagomys theodorverhoeveni	5519	2794	63	0	NaN	NaN	*0.000044*
Paraceros fragilis	65769	39969	118	0	NaN	NaN	*0.000044*

Paramylodon harlani	20979	20059	93	0	NaN	NaN	*0.000044*
Parocnus browni	6419	5019	31	0	NaN	NaN	*0.000044*
Parocnus serus	8405	7973	64	0	NaN	NaN	*0.000044*
Pelorovis antiquus	6249	4714	63	0	NaN	NaN	*0.000044*
Pennatomys nivalis	519	NaN	1	0	NaN	NaN	*0.000044*
Perameles eremiana	59	NaN	63	6	26900	NaN	0.000186
Peromyscus nesodytes	8069	8069	63	4	11250	5000	0.000480
Peromyscus pembertoni	88	NaN	63	0	NaN	NaN	*0.000044*
Peroryctes sp.	11124	10501	63	0	NaN	NaN	*0.000044*
Peroryctinae sp.	6259	5732	63	0	NaN	NaN	*0.000044*
Petauroides ayamaruensis	7960	7615	63	0	NaN	NaN	*0.000044*
Phanourios minutes	12718	9318	63	0	NaN	NaN	*0.000044*
Phascolonus gigas	52069	40069	39	11	53900	18800	0.000285
Phyllonycteris major	3757	3519	63	0	NaN	NaN	*0.000044*
Phyllops silvai	17636	17314	128	0	NaN	NaN	*0.000044*
Plagiodontia ipnaeum	483	NaN	63	0	NaN	NaN	*0.000044*
Plagiodontia spelaeum	11769	519	129	0	NaN	NaN	*0.000044*
Platygonus compressus	4809	3909	38	34	37000	1230	0.000923
Plesiorycteropus madagascariensis	2194	2194	106	0	NaN	NaN	*0.000044*
Pliomys lenki	12529	12529	130	0	NaN	NaN	*0.000044*
Potorous platyops	144	NaN	63	16	159199	1086	0.000095
Praemegaceros cazioti	7719	7599	63	0	NaN	NaN	*0.000044*
Procoptodon browneorum	35269	32069	39	37	159199	15687	0.000251
Procoptodon gilli	76069	64069	39	22	159199	5900	0.000137
Procoptodon goliah	52069	40069	39	1	159199	159199	*0.000069*
Procoptodon oreas	12759	11179	39	10	53900	11900	0.000214
Procoptodon rapha	16259	13779	39	5	53900	14950	0.000103
Prolagus sardus	245	NaN	63	0	NaN	NaN	*0.000044*
Propleopus oscillans	66069	51069	131	1	35000	35000	*0.000069*
Propraopus punctatus	44069	21069	116	0	NaN	NaN	*0.000044*
Propraopus sulcatus	8679	6899	63	2	26740	15500	0.000089

Protemnodon anak	40659	39439	132	44	91200	1500	0.000479
Protemnodon brehus	60069	44069	39	31	87800	4260	0.000359
Protemnodon hopei	18069	14069	112	3	42740	16000	0.000075
Protemnodon nombe	24069	14069	133	2	32100	29600	0.000400
Protemnodon roechus	52069	40069	39	20	136800	24100	0.000169
Protemnodon tumbuna	24069	14069	133	3	32100	27000	0.000392
Protocyon troglodytes	27069	10069	134	1	26740	26740	*0.000069*
Protopithecus brasiliensis	20419	19839	71	0	NaN	NaN	*0.000044*
Pseudomys glaucus	63	NaN	63	0	NaN	NaN	*0.000044*
Pseudomys gouldii	162	NaN	63	5	72300	3029	0.000058
Pteropus allenorum	163	NaN	63	0	NaN	NaN	*0.000044*
Pteropus brunneus	145	NaN	63	0	NaN	NaN	*0.000044*
Pteropus coxi	179	NaN	63	0	NaN	NaN	*0.000044*
Pteropus pilosus	145	NaN	63	0	NaN	NaN	*0.000044*
Pteropus subniger	149	NaN	63	0	NaN	NaN	*0.000044*
Pteropus tokudae	45	NaN	63	0	NaN	NaN	*0.000044*
Quemisia gravis	483	NaN	63	0	NaN	NaN	*0.000044*
Rattus macleari	116	NaN	63	0	NaN	NaN	*0.000044*
Rattus nativitatis	116	NaN	63	0	NaN	NaN	*0.000044*
Rattus sanila	1672	1484	63	0	NaN	NaN	*0.000044*
Rhagamys orthodon	2341	560	63	0	NaN	NaN	*0.000044*
Rhizoplagiodontia lemkei	4854	3710	31	0	NaN	NaN	*0.000044*
Rucervus schomburgki	81	NaN	63	0	NaN	NaN	*0.000044*
Rusingoryx atopocranion	46880	32544	79	0	NaN	NaN	*0.000044*
Sangamona fugitiva	10269	8749	135	5	35574	5780	0.000134
Scelidodon chiliensis	9179	8779	63	0	NaN	NaN	*0.000044*
Scelidotherium leptocephalum	8069	6069	136	0	NaN	NaN	*0.000044*
Simosthenurus maddocki	82069	70069	39	6	72300	33800	0.000130
Simosthenurus occidentalis	35269	32069	39	40	159199	9999	0.000261
Simosthenurus pales	46269	43669	39	10	136800	33800	0.000087
Sinomegaceros ordosianus	37309	33509	137	0	NaN	NaN	*0.000044*

Sinomegaceros yabei	17669	15909	86	0	NaN	NaN	*0.000044*
Sivacobus sankaliai	100069	50069	138	0	NaN	NaN	*0.000044*
Smilodon fatalis	12024	10374	38	19	37900	5900	0.000562
Smilodon populator	9349	9049	63	1	26740	26740	*0.000069*
Soergelia minor	26019	25549	81	0	NaN	NaN	*0.000044*
Solenodon marcanoi	519	NaN	63	0	NaN	NaN	*0.000044*
Solomys spriggsarum	9083	8524	63	0	NaN	NaN	*0.000044*
Spelaeomys florensis	5519	2794	63	0	NaN	NaN	*0.000044*
Spirocerus kiakhtensis	40069	30069	6	0	NaN	NaN	*0.000044*
Stegodon florensis	88069	62069	100	0	NaN	NaN	*0.000044*
Stegodon orientalis	8269	7499	139	0	NaN	NaN	*0.000044*
Stephanorhinus hemitoechus	28069	28069	140	1	87000	87000	*0.000069*
Stephanorhinus kirchbergensis	37069	28069	140	0	NaN	NaN	*0.000044*
Sthenurus andersoni	60069	44069	39	17	136800	19800	0.000137
Sthenurus atlas	60069	44069	39	1	41300	41300	*0.000069*
Sthenurus stirlingi	84069	66069	39	0	NaN	NaN	*0.000044*
Sthenurus tindalei	60069	44069	39	0	NaN	NaN	*0.000044*
Stockoceros conklingi	12669	7569	38	3	22560	10050	0.000160
Sus bucculentus	127	NaN	1	0	NaN	NaN	*0.000044*
Tapirus augustus	132069	109069	115	0	NaN	NaN	*0.000044*
Tapirus copei	12069	10869	38	0	NaN	NaN	*0.000044*
Tapirus rondoniensis	27579	27179	98	0	NaN	NaN	*0.000044*
Tapirus veroensis	15629	9509	38	11	27900	9840	0.000554
Tetrameryx shuleri	23789	22809	93	1	23230	23230	*0.000069*
Theriodictis tarijensis	44069	21069	116	0	NaN	NaN	*0.000044*
Thylacinus cynocephalus	83	NaN	63	84	159199	40	0.000521
Thylacoleo carnifex	52069	40069	39	49	159199	9999	0.000322
Thylogale christenseni	3757	3404	63	2	42740	3250	0.000025
Toxodon platensis	8069	3469	63	0	NaN	NaN	*0.000044*
Tremarctos floridanus	11219	10069	38	6	33660	12500	0.000236
Trigonodops lopesi	27579	27179	98	0	NaN	NaN	*0.000044*

Troposodon minor	125069	89069	141	14	87800	19500	0.000190
Ursus spelaeus	23969	23729	142	3	120000	32000	0.000023
Valgipes deformis	13129	12939	143	0	NaN	NaN	*0.000044*
Vombatus hacketti	35269	32069	39	16	136800	31500	0.000142
Wallabia kitcheneri	51069	39069	112	0	NaN	NaN	*0.000044*
Warendja wakefieldi	15589	14949	39	1	15200	15200	*0.000069*
Xaymaca fulvopulvis	11489	10159	111	0	NaN	NaN	*0.000044*
Xenorhinotherium bahiense	48069	30069	144	0	NaN	NaN	*0.000044*
Xenothrix mcgregori	250	NaN	63	0	NaN	NaN	*0.000044*
Zaglossus hacketti	57469	53069	39	3	55200	31500	0.000084
Zalophus japonicus	68	NaN	63	0	NaN	NaN	*0.000044*
Zygomaturus trilobus	36769	31969	56	45	159199	2770	0.000281
