# Supplementary material for: The past and future human impact on mammalian diversity
Source: Sci Adv. 2020 Sep 4;6(36):eabb2313. doi: 10.1126/sciadv.abb2313 (PMC7473673; doi:10.1126/sciadv.abb2313)
Supplement: abb2313_SM.pdf [file abb2313_SM.pdf]

Science Advances
AAAS

advances.sciencemag.org/cgi/content/full/6/36/eabb2313/DC1

Supplementary Materials for

The past and future human impact on mammalian diversity

Tobias Andermann*, Søren Faurby, Samuel T. Turvey, Alexandre Antonelli, Daniele Silvestro

*Corresponding author. Email: tobias.andermann@bioenv.gu.se

Published 4 September 2020, *Sci. Adv.* **6**, eabb2313 (2020)
DOI: 10.1126/sciadv.abb2313

The PDF file includes:

Tables S1 to S3
Figs. S1 to S10
Legends for data files S1 and S2
References

Other Supplementary Material for this manuscript includes the following:

(available at advances.sciencemag.org/cgi/content/full/6/36/eabb2313/DC1)

Data files S1 and S2

Region	Landmass	Area km^2	Max. arrival	Min. arrival	Source
Africa	Africa	29,202,077	NaN	NaN	*(5)*
Europe	Eurasia	9,219,614	48,000	40,000	*(5)*
Siberia	Eurasia	13,170,627	48,000	44,000	*(5)*
Central Asia	Eurasia	22,190,587	95,000	44,000	*(55)*
Indo-Malaya	Eurasia	7,725,279	73,000	44,000	*(56)*
Australia	Australia	8,449,464	65,000	44,000	*(24)*
Beringia	North America	1,497,896	24,000	24,000	*(25)*
North America	North America	19,236,408	20,000	12,000	*(5)*
South America	South America	18,076,518	16,000	8,000	*(5)*
Caribbean	Caribbean	214,288	7,000	4,000	*(28)*
Madagascar	Madagascar	590,729	10,000	4,000	*(5,27)*
Japan	Oceanic	293,500	24,000	20,000	*(5)*
Sulawesi	Oceanic	186,001	59,900	39,900	*(57)*
Greenland (no inland ice)	Oceanic	403,828	4,516	4,516	*(51)*
Iceland	Oceanic	102,950	1,142	1,142	*(52)*
New Zealand	Oceanic	267,535	2,216	736	*(53)*

Table S1. Size of regions of human occupation and range of possible arrival times.
The first column shows the regions that were used for calculating human land-occupation. The second column shows which landmass each region was assigned to in order to calculate the total area of all landmasses. These values were used for calculating past and expected future human population densities.

Region	Total species	Total species (extant)	Total extant fraction	Endemic species	Endemic species (extant)	Endemics extant fraction	Endemicity fraction
Africa	1195	1177	0.98	1027	1012	0.99	0.86
Eurasia	1479	1442	0.97	1268	1237	0.98	0.86
Australia	576	514	0.89	513	451	0.88	0.89
North America	872	819	0.94	595	555	0.93	0.68
South America	1293	1231	0.95	1055	1001	0.95	0.82
Caribbean	158	113	0.69	92	45	0.49	0.58
Madagascar	255	234	0.92	212	191	0.9	0.83

Table S2. Overview of endemicity for each region analyzed in this study.
Only endemic species were analyzed for each region in all analyses. Since endemicity of extinct and extant species was modeled in the same manner in this study, the fraction of extant species largely remains constant before and after filtering only endemics for further processing (compare columns 'Total extant fraction' and 'Endemics extant fraction'). This confirms that processing only endemics for each region will not bias the resulting extinction rates, as the ratio between extinct and extant taxa remains constant. The only exception is the Caribbean, for which the fraction of extant taxa decreases after filtering only endemic species.

Subset	Constant	1 shift	2 shifts	3 shifts	4 shifts	5 shifts
Global	0.000	0.000	0.000	**0.056**	**0.943**	0.001
Africa	**0.935**	**0.065**	0.000	0.000	0.000	0.000
Eurasia	**0.158**	**0.838**	0.004	0.000	0.000	0.000
Australia	0.000	**0.858**	**0.142**	0.000	0.000	0.000
North America	0.000	**1.000**	0.000	0.000	0.000	0.000
South America	0.000	**0.966**	0.034	0.000	0.000	0.000
Caribbean	0.000	0.016	**0.983**	0.001	0.000	0.000
Madagascar	0.000	**0.999**	0.001	0.000	0.000	0.000
Carnivora	0.000	**1.000**	0.000	0.000	0.000	0.000
Cetartiodactyla	0.000	**0.907**	**0.093**	0.000	0.000	0.000
Chiroptera	0.002	**0.997**	0.001	0.000	0.000	0.000
Cingulata	0.015	**0.985**	0.000	0.000	0.000	0.000
Diprotodontia	0.003	**0.910**	**0.086**	0.000	0.000	0.000
Eulipotyphla	0.008	**0.992**	0.000	0.000	0.000	0.000
Peramelemorphia	**0.744**	**0.256**	0.000	0.000	0.000	0.000
Perissodactyla	**0.555**	**0.445**	0.000	0.000	0.000	0.000
Pilosa	0.000	**0.997**	0.003	0.000	0.000	0.000
Primates	0.000	**0.995**	0.005	0.000	0.000	0.000
Proboscidea	0.000	**1.000**	0.000	0.000	0.000	0.000
Rodentia	0.000	0.000	**0.963**	0.037	0.000	0.000

Table S3. Posterior probability of different shift models resulting from our rate shift model. Marked in bold are all models that were supported by more than 5% posterior probability.

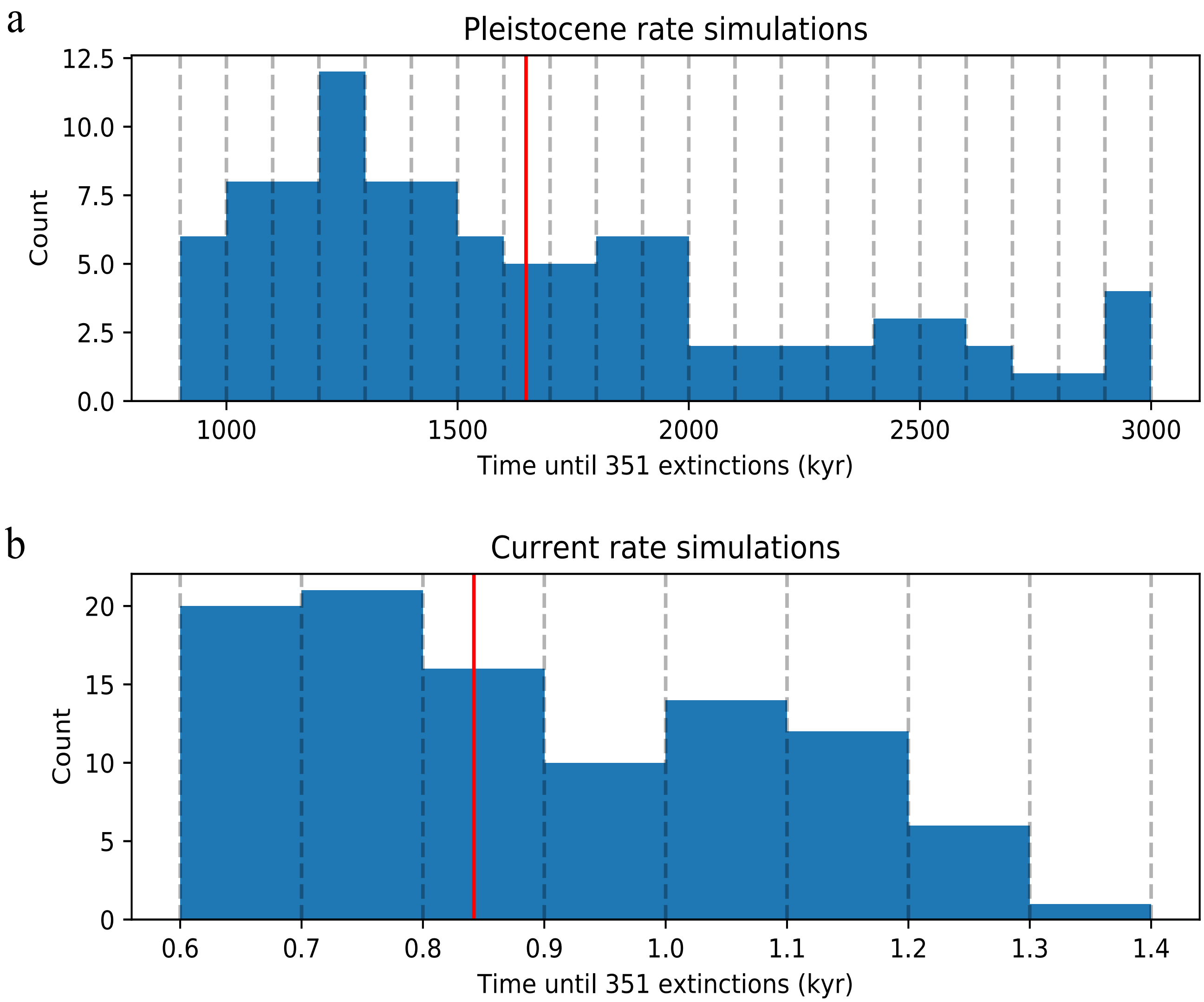

Fig. S1. Simulated time until 351 extinctions using different rates.
We simulated how long it would take for the 351 mammal species extinctions observed in the empirical data throughout the last 126 kyr to occur under different rate scenarios. Shown are histograms (blue bars) and the mean values (red line) of the estimates of 100 simulations under a) the extinction rate from the beginning of the Late Pleistocene, and b) the extinction rate estimated at present. We estimated the average time for the observed 351 mammal extinctions to occur under Late Pleistocene rates at 1.75 Myr (0.95–2.99 Myr, 95% confidence interval), whereas under the highly elevated current rates it would take only 810 yr (500–1,100 yr). The rate estimates for the simulations were sampled from the 95% HPD interval of the posterior rate distribution resulting from the shift-model analyses of the complete fossil record of the last 126 kyr.

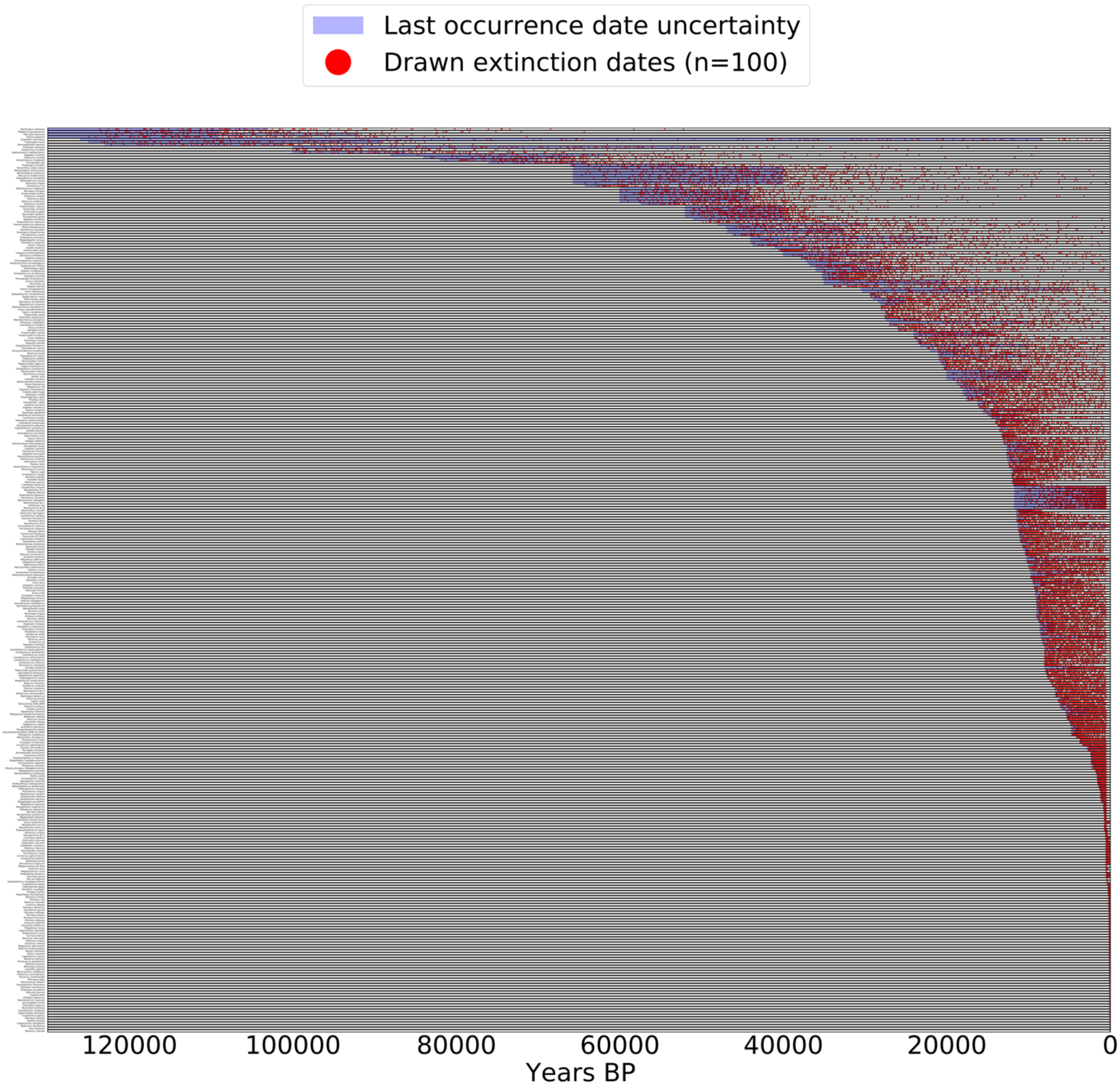

Fig. S2. Last occurrence range and drawn extinction dates for extinct mammal species.
Each row represents one of the 351 extinct mammal species included in this study. The range shaded in blue shows the uncertainty surrounding the dating of the last occurrence of a species. If no blue range is visible for a species, the age of the fossil occurrence was provided with high precision (no uncertainty interval) in the source literature. Red dots show the 100 independently drawn extinction dates (dataset replicates). For a high-resolution version of this plot, see:
https://github.com/tobiashofmann88/mammal_extinction_supporting_info

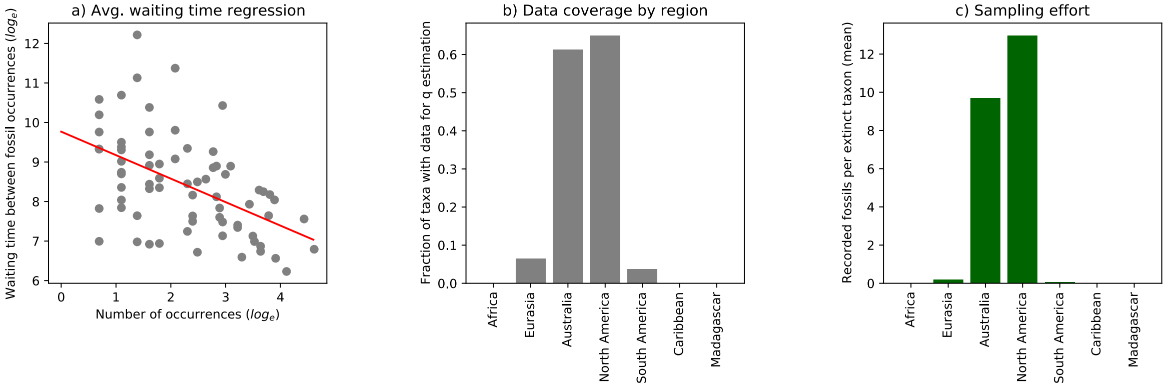

Fig. S3. Overview of the fossil database data used for calculation of preservation rates. We calculated preservation rates for individual species by dividing the number of occurrences of a species in the fossil record since the beginning of the Middle Pleistocene (781 kyr ago) by the length of the time interval between the first and last occurrence of the species in the fossil record. This rate represents the inverse of the average waiting time between two fossils of a given species and is thus a measure of the sampling frequency of individual species. We calculated the average waiting times for taxa with two or more fossil occurrences and plotted these against the number of fossil occurrences of the respective species (a). The line regression (red line) was calculated for the log-transformed (natural log: log_e) values of both axes. The purpose of this regression was to model a preservation rate for taxa with only a single or no fossil occurrence, since for those taxa the preservation rate could not be calculated in the same manner as for other taxa. We regressed the average waiting times (inverse of rates) instead of the actual rates, because the harmonic mean (i.e. the inverse of the arithmetic mean of the inverse values) better represents the average of rates. Taxa with two or more fossil occurrences for which preservation rates could be directly calculated vary strongly by region (b), representing geographic biases in availability of fossil data. To further demonstrate regional sampling biases, the last panel (c) shows the average number of fossils per extinct taxon by region. While North America has the most densely sampled fossil record over the observed time frame (Middle Pleistocene until today), South America has a much lower number of occurrences of extinct species in the fossil record, which on average leads to larger uncertainties in the modeled extinction dates, due to small preservation rates.

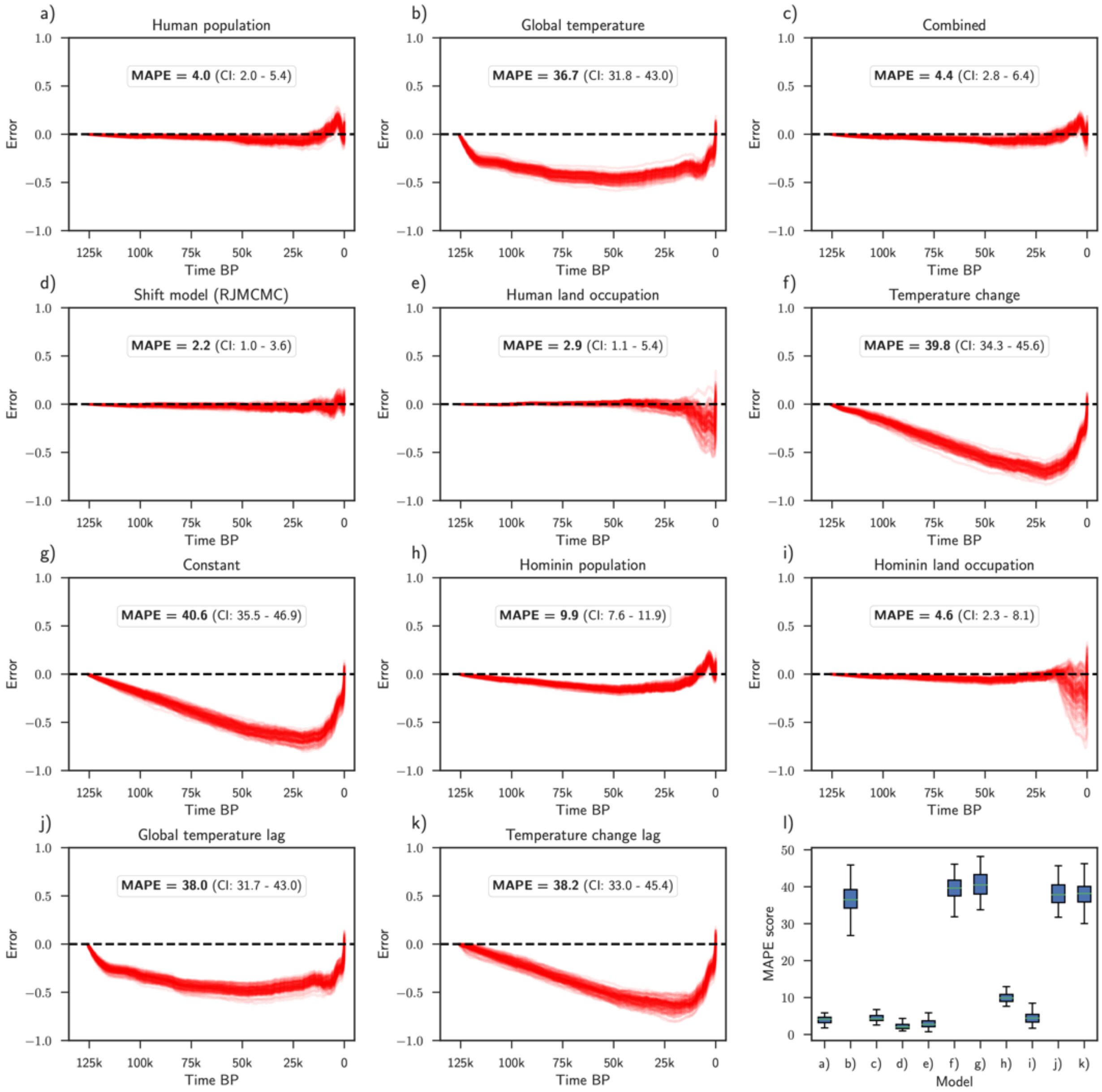

Fig. S4. Model adequacy of all tested correlation models and of the rate-shift model.
We applied the extinction rates through time that were estimated with each of the tested models to simulate past extinctions under those rates (100 simulation replicates for each model). All simulations started at the same baseline of 6065 species, which is the empirical species diversity at 126 kyr ago based on our extinction data. We then compared the predicted diversity through time with the empirical diversity through time array resulting from our extinction data. In order to do so we calculated the error between those two curves relative to the total number of extinctions: (simulated diversity - empirical diversity)/351 species. The resulting errors through time for all simulation replicates are shown as red lines for the different models: human population (a), global temperature (b), mixed model containing human pop. and temperature (c), the shift model (d), human land occupation (e), temperature change (f), the constant null model (g), hominin population (h), hominin land occupation (i), global temperature with time-lag (j), and temperature change with time-lag (k). We calculated the mean absolute percentage error (MAPE) for each simulation replicate by taking the mean of the absolute errors through time and multiplying it by 100 to transform it into a percentage error. Panel l) shows the range of the estimated MAPE scores for each model (median values marked as orange lines, blue box displays the interquartile range, black whiskers show minimum and maximum values of the range). Low MAPE scores show (< 5%) demonstrate high accuracy of the respective model, for example a MAPE score of 4.0 translates into 96.0% accuracy of the model in predicting past extinctions.

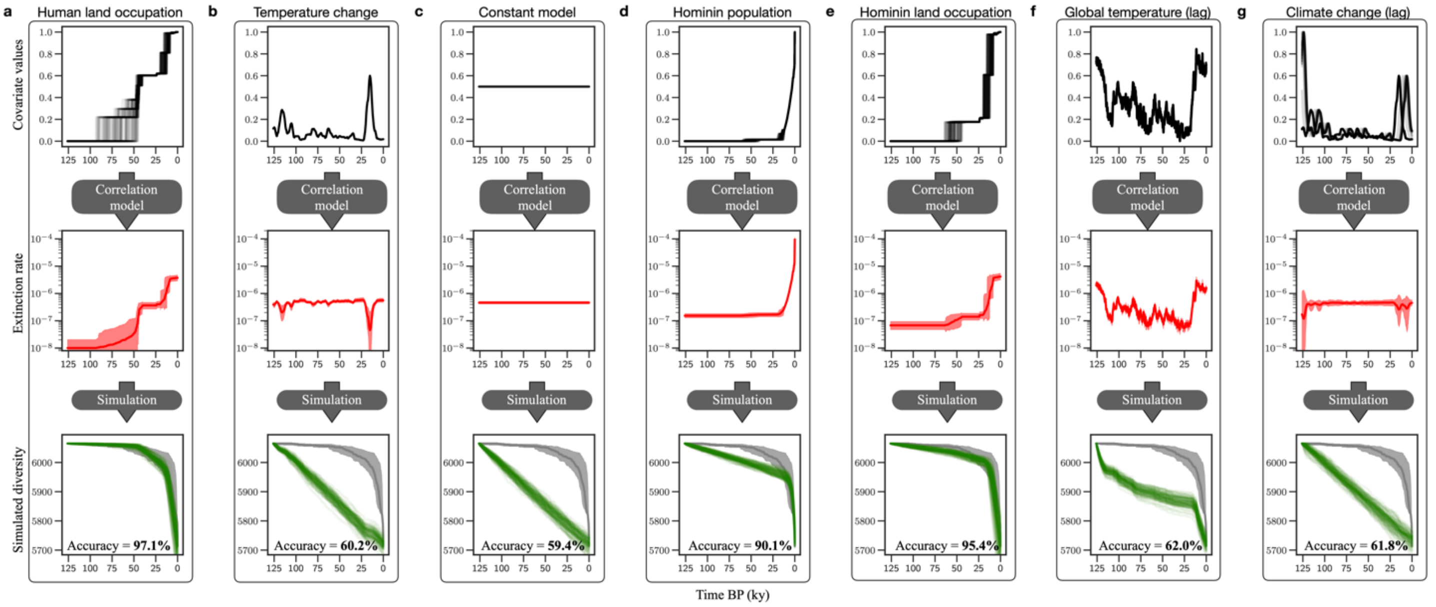

Fig. S5. Overview of additional correlation models tested in this study.
Correlation model results for the human land occupation model (a), the variance of temperature change model (b), a constant model (c), the hominin population density model (d), the hominin land occupation model (e), a global temperature model allowing for a time-lag in the response variable (extinction rates) (f), as well as a time-lag climate change model (g). The panels display the re-scaled predictor values (black, upper panels), the estimated extinction rates (red, center panels, mean and 95% HPD), and the 100 simulated past diversity trajectories based on these rates (green, lower panels) for each correlation model. For reference the bottom panels also show the empirical diversity trajectory (mean and 95% confidence interval) based on the data compiled in this study (grey). As a measure of how well each model predicted past extinction dynamics, the bottom panels show the prediction accuracy calculated from the mean absolute percentage error of all simulation replicates (see Fig. S4). For our time-lag models (f-g), the average inferred time-lag on extinctions for global temperature was estimated at 135 years (std=132), while for temperature change it was estimated at 3452 years (std= 4229). Compared to the temporally linked climate correlation models (b, Fig. 2c), allowing for a time-lag in the response variable does not lead to an increase in model accuracy. In the time-lag climate change model (g), the correlation factor switched between positive and negative values between MCMC replicates, leading to the peaks in uncertainty intervals into both directions observed in the rate plot. The large peak at the beginning of the climate change rate plot is due to a large peak in the covariate values prior to the displayed time frame, which impacted the extinction rates when the time-lag was estimated at a large value.

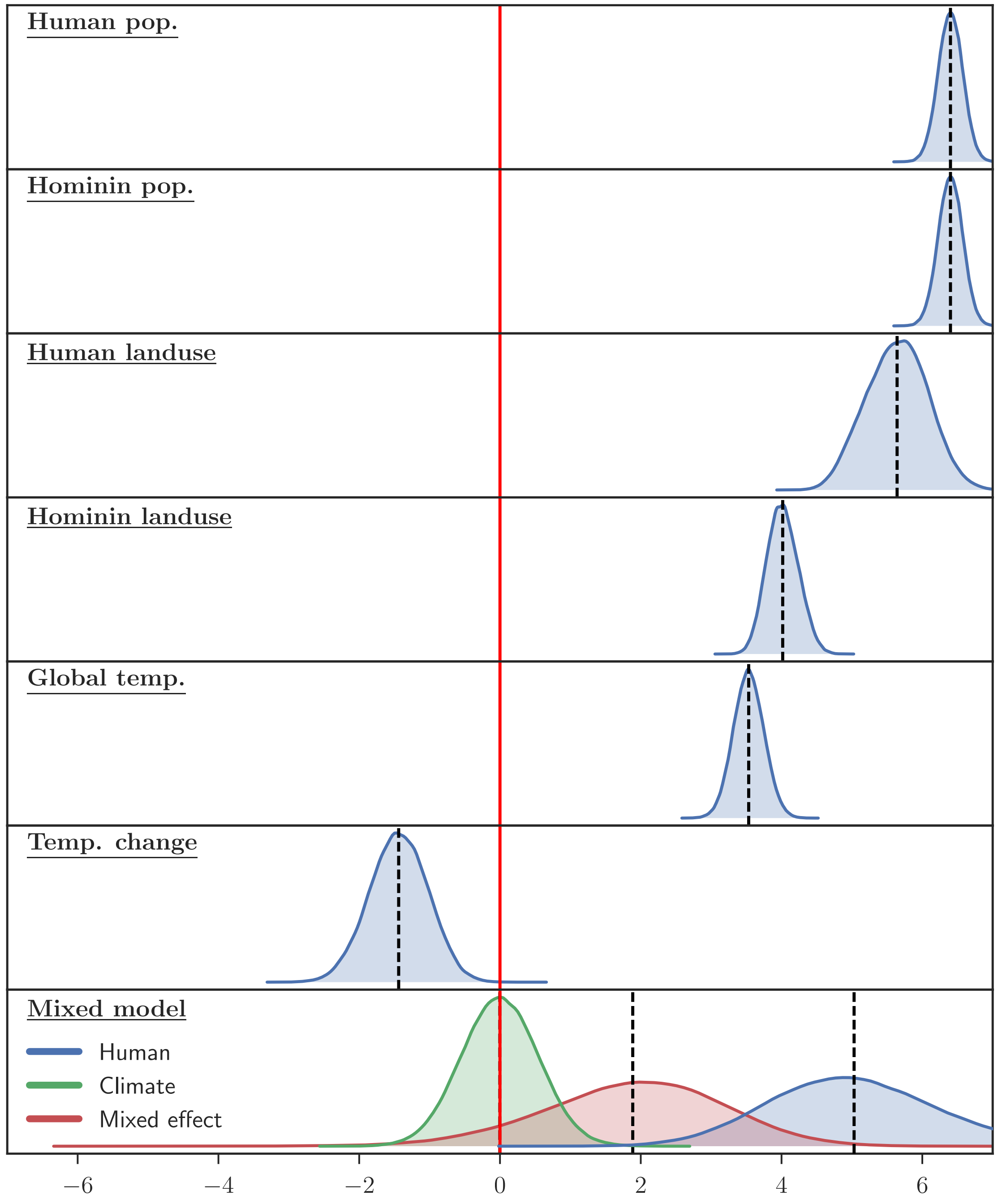

Fig. S6. Density plots of correlation factors estimated from different correlation models.
Positive correlation factors indicate a positive correlation between the correlation variable and mammalian extinction rates, while negative values indicate a negative correlation. Deviations from the zero line (red) indicate significant correlations. In the mixed model (bottom panel), three correlation factors were estimated for humans, climate and the interaction of humans and climate respectively.

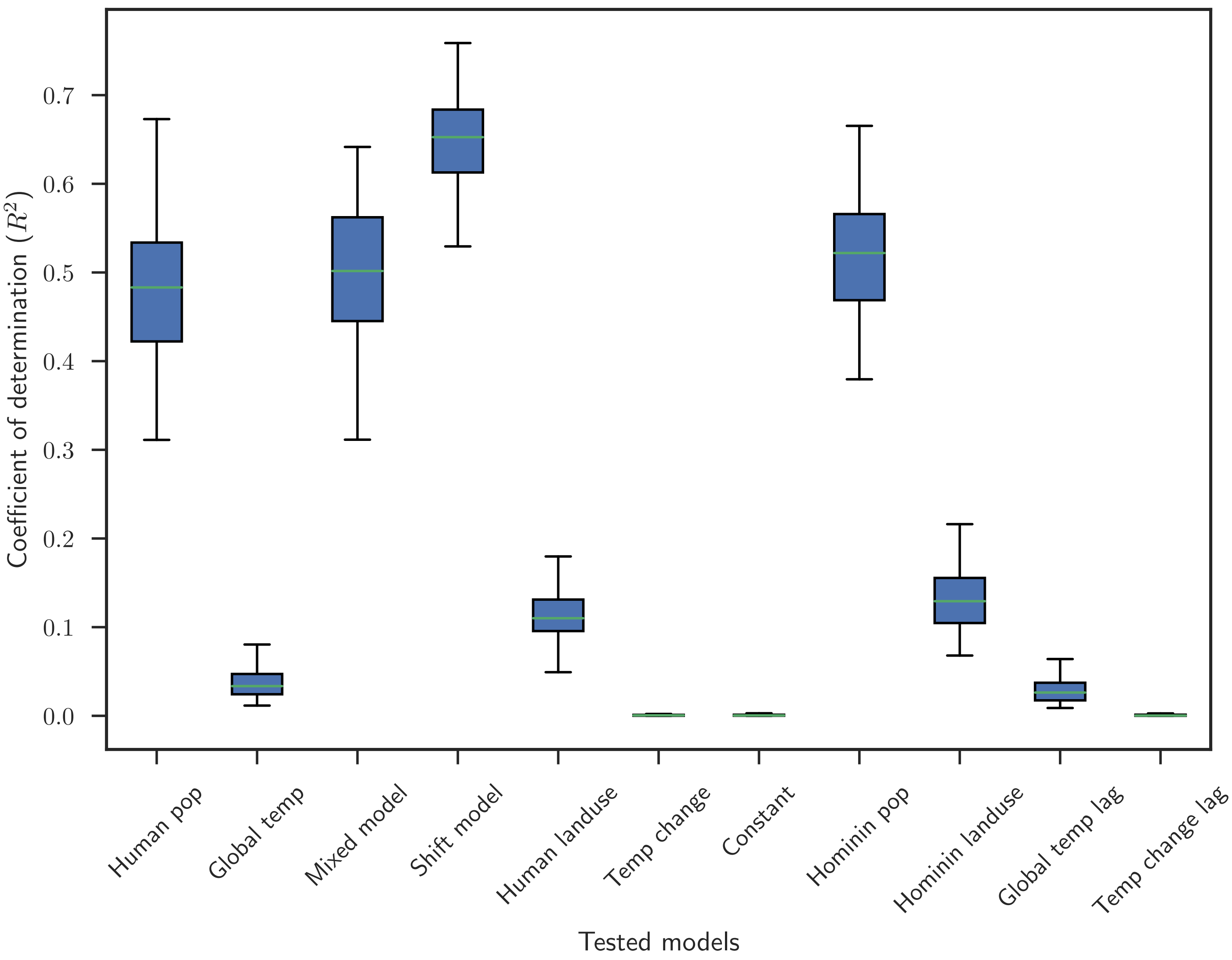

Fig. S7. Coefficients of determination (R2) for the simulated past diversity trajectories under all tested correlation models.

Since the diversity values (y) are temporally autocorrelated, which can inflate the R2 values, we first detrended the diversity data (y') in the following manner: $y'_t = y_t - y_{t-1}$.Using the detrended diversity arrays, we computed the R2 between the predicted values and the empirical diversity data as a measure of how well each model explains the past decline of mammalian diversity. The boxplot shows the mean and the standard deviation of the 100 calculated R2-values for all tested correlation models. The R2 values can be interpreted as the fraction of the past extinction dynamics that can be explained by the single predictor in each model.

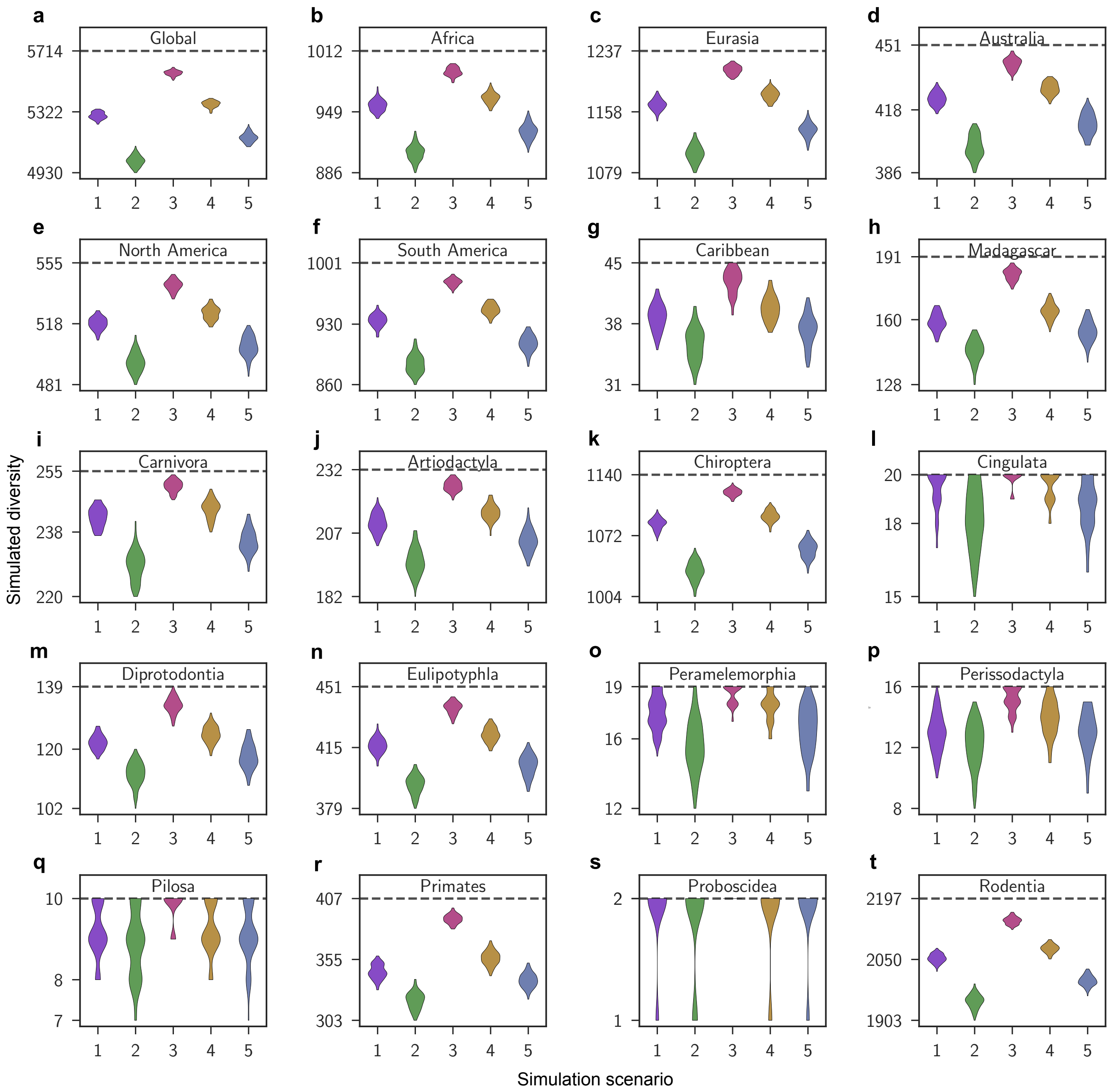

Fig. S8. Different IUCN-based future diversity scenarios for 2100 CE for all subsets.
The colored violin plots show the density of the 99% confidence interval of diversity predictions for the spatial and taxonomic mammal subsets that were analyzed in this study: global (a), Africa (b), Eurasia (c), Australia (d), North America (e), South America (f), Caribbean (g), Madagascar (h), Carnivora (only terrestrial) (i), Cetartiodactyla (only terrestrial) (j), Chiroptera (k), Cingulata (l), Diprotodontia (m), Eulipotyphla (n), Peramelemorphia (o), Perissodactyla (p), Pilosa (q), Primates (r), Proboscidea (s), and Rodentia (t). We simulated five different scenarios for each subset, using the software iucn_sim. The first scenario (1) 'Continuing trends' represents the iucn_sim default scenario, where the rates of IUCN status changes of the past 20 years are projected into the future. In the second scenario (2) 'Stable status' species are simulated to not change their threat status throughout the simulated time frame. The third scenario (3) 'No additional threats' only allows for future improvements of IUCN statuses (no worsening) and contrarily the fourth scenario (4) 'No conservation' only allows future worsening (no improvements). The fifth scenario (5) '10x conservation increase' increases the rates leading to improvements of IUCN statuses by factor 10.

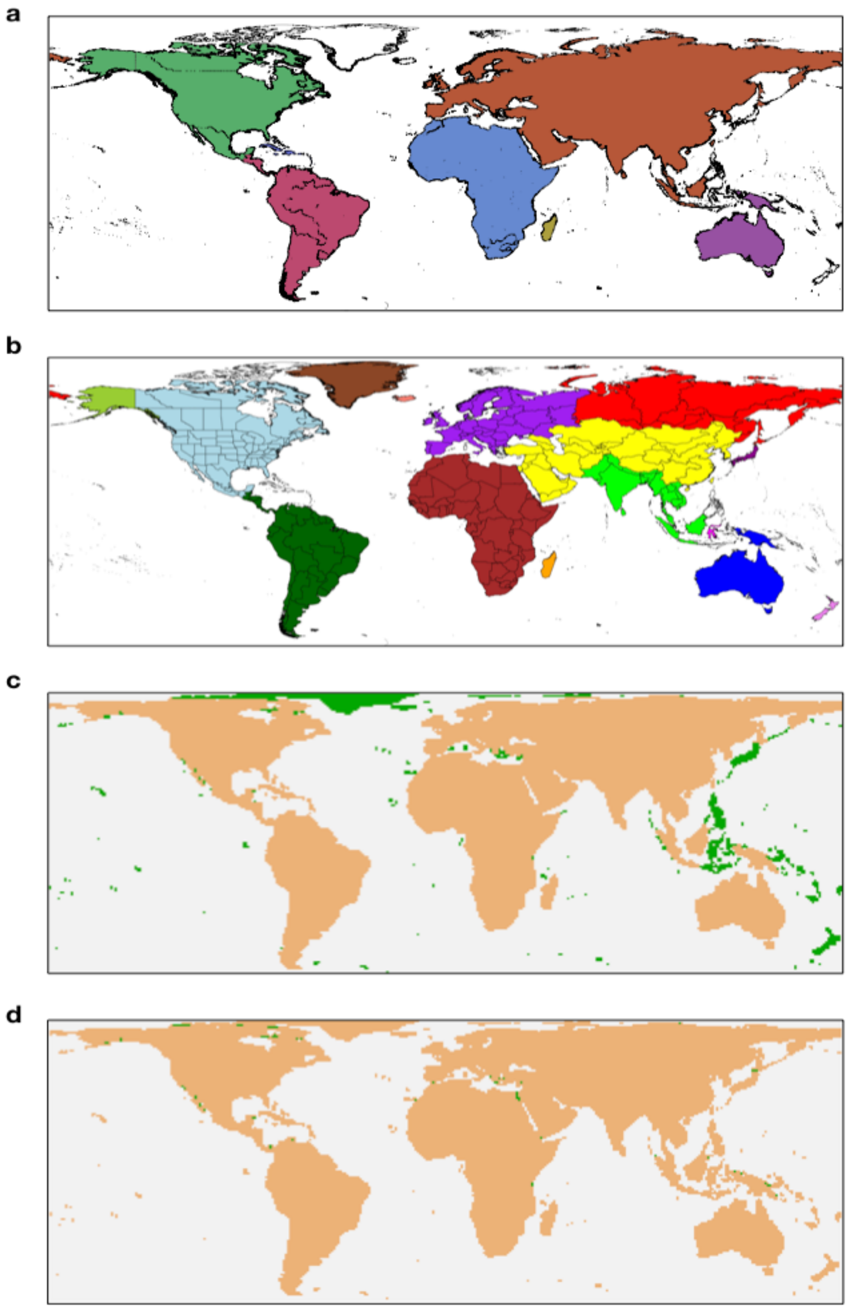

Fig. S9. Overview of spatial definitions used in this study.
Shown are the spatial shape maps of a) the seven regions of mammal endemism: Africa, Eurasia, Australia, North America, South America, Caribbean, and Madagascar and b) the bioregions of human expansion, used to model our human land occupation data. The other two panels show highlighted cells in our 100×100 km grid. In the case of c), cells colored in green show non-assigned land-cells, not belonging to any of the six regions of mammal endemism, and highlighted in d) are ambiguous land-cells that cannot be uniquely assigned to one of the seven major regions, often due to overlap with islands that are not connected to the region of question.

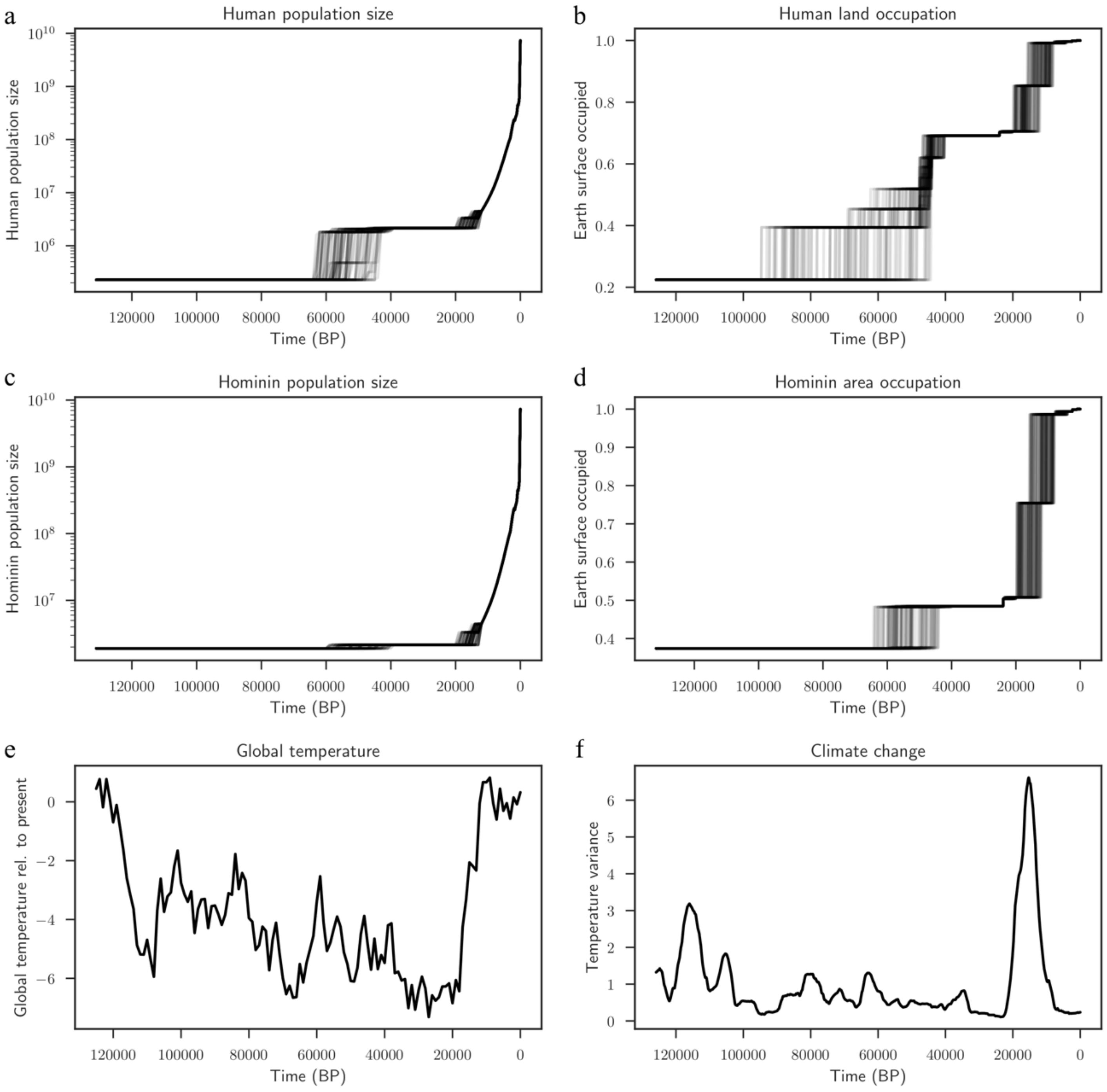

Fig. S10. Plots of time-continuous correlation variables as applied in our correlation models.
For the human and hominin correlation variables (a-d, see plot titles) we modeled 100 independent data arrays in order to account for uncertainties in human arrival times, which affect our modeling. The climate correlation variables (e and f) are based on the mean values of the AICC2012 model. Shown are the absolute values of each correlation variable. Note that for standardization purposes the values of each variable are re-scaled between 0 and 1 before input into the correlation models.

Data S1. (separate file)
Diversity and extinction rates (red, lower panels) through time for all data subsets analyzed in this study. Shown are all 100 diversity data replicates drawn from the last occurrence data compiled in this study (black, upper panels). Lower panels (red) show extinction rates, plotted in log-space, showing the mean (solid red line) and the 95% HPD interval (red shaded area) of the estimated rates. Rates were summarized across all shift models that were explored by the MCMC algorithm, as opposed to summarizing the rates across only specific rate shift models, as done in Fig. 1.

Data S2. (separate file)

Last occurrence dates and preservation rates (q) for recently extinct mammal species. The LO_{max} and LO_{min} columns show the range of the dating uncertainty of the last occurrence (LO) of each taxon (fossil or observation), which were compiled in this study from the peer-reviewed literature (*1*, *6*, *31*, *38*, *39*, *56*, *63–144*). These dates are scaled in years before the year 2019 CE to synchronize the time axis with the IUCN-2019-v3 data, rather than using the more common convention of expressing dates in years BP (years before 1950 CE). Minimum and maximum age were set to the same date in those cases where no dating uncertainty was specified in the referenced source of the LO date. For each taxon we estimated specific preservation rates (q), based on the number of fossil occurrences recorded in public fossil databases (N, only including high dating precision occurrences of the Mid-Pleistocene or younger) and the date of first and last occurrence in these databases (FO_{DB} and LO_{DB}) using the formula $q = (N - 1)/(FO_{DB} - LO_{DB})$. For taxa with N=0 or N=1 occurrences in the databases and in cases where $FO_{DB} = LO_{DB}$, we approximated preservation rates based on a linear regression between N and the average waiting times between two fossil occurrences (1/q) for all taxa (q in italics for taxa modelled in this manner). The preservation rates, which are a measure of the fossilization potential and sampling frequency of each species, were used to model extinction dates based on the manually compiled LO dates. Species with no specified LO_{min} age are known from last sightings since the year 1500 CE and were modeled with an open minimum boundary, thereby allowing a small chance of rediscovery.

REFERENCES AND NOTES

1. IUCN, IUCN Red List of Threatened Species, version 2019-3 (2019); www.iucnredlist.org/en.

2. S. Faurby, M. Davis, R. Ø. Pedersen, S. D. Schowanek, A. Antonelli, J.-C. Svenning, PHYLACINE 1.2: The Phylogenetic Atlas of Mammal Macroecology. *Ecology* **99**, 2626 (2018).

3. A. D. Barnosky, N. Matzke, S. Tomiya, G. O. U. Wogan, B. Swartz, T. B. Quental, C. Marshall, J. L. McGuire, E. L. Lindsey, K. C. Maguire, B. Mersey, E. A. Ferrer, Has the Earth's sixth mass extinction already arrived? *Nature* **471**, 51–57 (2011).

4. A. D. Barnosky, P. L. Koch, R. S. Feranec, S. L. Wing, A. B. Shabel, Assessing the causes of late Pleistocene extinctions on the continents. *Science* **306**, 70–75 (2004).

5. L. J. Bartlett, D. R. Williams, G. W. Prescott, A. Balmford, R. E. Green, A. Eriksson, P. J. Valdes, J. S. Singarayer, A. Manica, Robustness despite uncertainty: Regional climate data reveal the dominant role of humans in explaining global extinctions of Late Quaternary megafauna. *Ecography* **39**, 152–161 (2016).

6. G. W. Prescott, D. R. Williams, A. Balmford, R. E. Green, A. Manica, Quantitative global analysis of the role of climate and people in explaining late Quaternary megafaunal extinctions. *Proc. Natl. Acad. Sci. U.S.A.* **109**, 4527–4531 (2012).

7. G. Ceballos, A. García, P. R. Ehrlich, The sixth extinction crisis: Loss of animal populations and species. *J. Cosmol.* **8**, 1821–1831 (2010).

8. M. Foote, Temporal variation in extinction risk and temporal scaling of extinction metrics. *Paleobiology* **20**, 424–444 (1994).

9. J. Alroy, Speciation and extinction in the fossil record of North American mammals, in *Speciation and Patterns of Diversity*, R. Butlin, J. Bridle, D. Schluter, Eds. (Cambridge Univ. Press, Cambridge, UK, New York, 2009), pp. 301–323.

10. C. N. Johnson, Determinants of loss of mammal species during the Late Quaternary 'megafauna' extinctions: Life history and ecology, but not body size. *Proc. R. Soc. B* **269**, 2221–2227 (2002).

11. C. Sandom, S. Faurby, B. Sandel, J.-C. Svenning, Global late Quaternary megafauna extinctions linked to humans, not climate change. *Proc. R. Soc. B* **281**, 20133254 (2014).

12. F. A. Smith, R. E. E. Smith, S. K. Lyons, J. L. Payne, Body size downgrading of mammals over the late Quaternary. *Science* **360**, 310–313 (2018).

13. P. Martin, H. Wright, Pleistocene extinctions. The search for a cause, in *Proceedings of 7th Congress International Association for Quaternary Research* (Yale Univ. Press, 1967), vol. 6.

14. P. S. Martin, Prehistoric overkill: The global model, in *Quaternary Extinctions: A Prehistoric Revolution*, P. S. Martin, R. G. Klein, Eds. (University of Arizona Press, 1984), pp, 354–403.

15. J. Alroy, A multispecies overkill simulation of the end-pleistocene megafaunal mass extinction. *Science* **292**, 1893–1896 (2001).

16. M. S. Lima-Ribeiro, J. A. Felizola Diniz-Filho, American megafaunal extinctions and human arrival: Improved evaluation using a meta-analytical approach. *Quat. Int.* **299**, 38–52 (2013).

17. S. Wroe, J. Field, D. K. Grayson, Megafaunal extinction: Climate, humans and assumptions. *Trends Ecol. Evol.* **21**, 61–62 (2006).

18. S. Wroe, J. Field, A review of the evidence for a human role in the extinction of Australian megafauna and an alternative interpretation. *Quat. Sci. Rev*. **25**, 2692–2703 (2006).

19. E. D. Lorenzen, D. Nogués-Bravo, L. Orlando, J. Weinstock, J. Binladen, K. A. Marske, A. Ugan, M. K. Borregaard, M. T. P. Gilbert, R. Nielsen, S. Y. W. Ho, T. Goebel, K. E. Graf, D. Byers, J. T. Stenderup, M. Rasmussen, P. F. Campos, J. A. Leonard, K.-P. Koepfli, D. Froese, G. Zazula, T. W. Stafford Jr., K. Aaris-Sørensen, P. Batra, A. M. Haywood, J. S. Singarayer, P. J. Valdes, G. Boeskorov, J. A. Burns, S. P. Davydov, J. Haile, D. L. Jenkins, P. Kosintsev, T. Kuznetsova, X. Lai, L. D. Martin, H. G. McDonald, D. Mol, M. Meldgaard, K. Munch, E. Stephan, M. Sablin, R. S. Sommer, T. Sipko, E. Scott, M. A. Suchard, A. Tikhonov, R. Willerslev, R. K. Wayne, A. Cooper, M. Hofreiter, A. Sher, B. Shapiro, C. Rahbek, E. Willerslev, Species-specific responses of late Quaternary megafauna to climate and humans. *Nature* **479**, 359–364 (2011).

20. M. J. Monroe, S. H. M. Butchart, A. O. Mooers, F. Bokma, The dynamics underlying avian extinction trajectories forecast a wave of extinctions. *Biol. Lett.* **15**, 20190633 (2019).

21. S. Faurby, J.-C. Svenning, Historic and prehistoric human-driven extinctions have reshaped global mammal diversity patterns. *Divers. Distrib*. **21**, 1155–1166 (2015).

22. P. W. Signor III, J. H. Lipps, L. Silver, P. Schultz, Sampling bias, gradual extinction patterns and catastrophes in the fossil record. *Geological Society of America Special Paper* **190**, 291–296 (1982).

23. D. Silvestro, N. Salamin, A. Antonelli, X. Meyer, Improved estimation of macroevolutionary rates from fossil data using a Bayesian framework. *Paleobiology* **45**, 546–570 (2019).

24. C. Clarkson, Z. Jacobs, B. Marwick, R. Fullagar, L. Wallis, M. Smith, R. G. Roberts, E. Hayes, K. Lowe, X. Carah, S. A. Florin, J. M. Neil, D. Cox, L. J. Arnold, Q. Hua, J. Huntley, H. E. A. Brand, T. Manne, A. Fairbairn, J. Shulmeister, L. Lyle, M. Salinas, M. Page, K. Connell, G. Park, K. Norman, T. Murphy, C. Pardoe, Human occupation of northern Australia by 65,000 years ago. *Nature* **547**, 306–310 (2017).

25. L. Bourgeon, A. Burke, T. Higham, Earliest human presence in North America dated to the Last Glacial Maximum: New radiocarbon dates from Bluefish Caves, Canada. *PLOS ONE* **12**, e0169486 (2017).

26. S. Faurby, D. Silvestro, L. Werdelin, A. Antonelli, Brain expansion in early hominins predicts carnivore extinctions in East Africa. *Ecol. Lett.* **23**, 537–544 (2020).

27. J. Hansford, P. C. Wright, A. Rasoamiaramanana, V. R. Pérez, L. R. Godfrey, D. Errickson, T. Thompson, S. T. Turvey, Early Holocene human presence in Madagascar evidenced by exploitation of avian megafauna. *Sci. Adv.* **4**, eaat6925 (2018).

28. S. B. Cooke, L. M. Dávalos, A. M. Mychajliw, S. T. Turvey, N. S. Upham, Anthropogenic extinction dominates Holocene declines of West Indian mammals. *Annu. Rev. Ecol. Evol. Syst.* **48**, 301–327 (2017).

29. D. K. Grayson, D. J. Meltzer, A requiem for North American overkill. *J. Archaeol. Sci.* **30**, 585–593 (2003).

30. M. Cardillo, G. M. Mace, J. L. Gittleman, K. E. Jones, J. Bielby, A. Purvis, The predictability of extinction: Biological and external correlates of decline in mammals. *Proc. R. Soc. B* **275**, 1441–1448 (2008).

31. S. T. Turvey, S. A. Fritz, The ghosts of mammals past: Biological and geographical patterns of global mammalian extinction across the Holocene. *Philos. Trans. R. Soc. B* **366**, 2564–2576 (2011).

32. S. Rule, B. W. Brook, S. G. Haberle, C. S. M. Turney, A. P. Kershaw, C. N. Johnson, The aftermath of megafaunal extinction: Ecosystem transformation in Pleistocene Australia. *Science* **335**, 1483–1486 (2012).

33. F. Saltré, M. Rodríguez-Rey, B. W. Brook, C. N. Johnson, C. S. M. Turney, J. Alroy, A. Cooper, N. Beeton, M. I. Bird, D. A. Fordham, R. Gillespie, S. Herrando-Pérez, Z. Jacobs, G. H. Miller, D. Nogués-Bravo, G. J. Prideaux, R. G. Roberts, C. J. A. Bradshaw, Climate change not to blame for late Quaternary megafauna extinctions in Australia. *Nat. Commun.* **7**, 10511 (2016).

34. D. Veres, L. Bazin, A. Landais, H. T. M. Kele, B. Lemieux-Dudon, F. Parrenin, P. Martinerie, E. Blayo, T. Blunier, E. Capron, J. Chappellaz, S. O. Rasmussen, M. Severi, A. Svensson, B. Vinther, E. W. Wolff, The Antarctic ice core chronology (AICC2012): An optimized multi-parameter and multi-site dating approach for the last 120 thousand years. *Clim. Past* **9**, 1733–1748 (2013).

35. D. Tilman, R. M. May, C. L. Lehman, M. A. Nowak, Habitat destruction and the extinction debt. *Nature* **371**, 65–66 (1994).

36. C. E. Doughty, S. Faurby, A. Wolf, Y. Malhi, J.-C. Svenning, Changing NPP consumption patterns in the Holocene: From megafauna-'liberated' NPP to 'ecological bankruptcy'. *Anthropocene Rev.* **3**, 174–187 (2016).

37. S. Díaz, J. Settele, E. S. Brondízio, H. T. Ngo, J. Agard, A. Arneth, P. Balvanera, K. A. Brauman, S. H. M. Butchart, K. M. A. Chan, L. A. Garibaldi, K. Ichii, J. Liu, S. M. Subramanian, G. F. Midgley, P. Miloslavich, Z. Molnár, D. Obura, A. Pfaff, S. Polasky, A. Purvis, J. Razzaque, B. Reyers, R. R. Chowdhury, Y.-J. Shin, I. Visseren-Hamakers, K. J. Willis, C. N. Zayas, Pervasive human-driven decline of life on Earth points to the need for transformative change. *Science* **366**, eaax3100 (2019).

38. J. W. Williams, E. C. Grimm, J. L. Blois, D. F. Charles, E. B. Davis, S. J. Goring, R. W. Graham, A. J. Smith, M. Anderson, J. Arroyo-Cabrales, A. C. Ashworth, J. L. Betancourt, B. W. Bills, R. K. Booth, P. I. Buckland, B. B. Curry, T. Giesecke, S. T. Jackson, C. Latorre, J. Nichols, T. Purdum, R. E. Roth, M. Stryker, H. Takahara, The Neotoma paleoecology database, a multiproxy, international, community-curated data resource. *Quat. Res.* **89**, 156–177 (2018).

39. M. Rodríguez-Rey, S. Herrando-Pérez, B. W. Brook, F. Saltré, J. Alroy, N. Beeton, M. I. Bird, A. Cooper, R. Gillespie, Z. Jacobs, C. N. Johnson, G. H. Miller, G. J. Prideaux, R. G. Roberts, C. S. M. Turney, C. J. A. Bradshaw, A comprehensive database of quality-rated fossil ages for Sahul's Quaternary vertebrates. *Sci. Data* **3**, 160053 (2016).

40. D. Strauss, P. M. Sadler, Classical confidence intervals and Bayesian probability estimates for ends of local taxon ranges. *Math. Geol.* **21**, 411–427 (1989).

41. D. Silvestro, S. Castiglione, A. Mondanaro, C. Serio, M. Melchionna, P. Piras, M. D. Febbraro, F. Carotenuto, L. Rook, P. Raia, A 450 million years long latitudinal gradient in age-dependent extinction. *Ecol. Lett.* **23**, 439–446 (2020).

42. H. D. Loxdale, B. J. Davis, R. A. Davis, Known knowns and unknowns in biology. *Biol. J. Linn. Soc.* **117**, 386–398 (2016).

43. D. Silvestro, J. Schnitzler, L. H. Liow, A. Antonelli, N. Salamin, Bayesian estimation of speciation and extinction from incomplete fossil occurrence data. *Syst. Biol.* **63**, 349–367 (2014).

44. O. Hagen, T. Andermann, T. B. Quental, A. Antonelli, D. Silvestro, Estimating age-dependent extinction: Contrasting evidence from fossils and phylogenies. *Syst. Biol.* **67**, 458–474 (2018).

45. S. Faurby, J.-C. Svenning, Resurrection of the island rule: Human-driven extinctions have obscured a basic evolutionary pattern. *Am. Nat.* **187**, 812–820 (2016).

46. K. Klein Goldewijk, A. Beusen, G. van Drecht, M. de Vos, The HYDE 3.1 spatially explicit database of human-induced global land-use change over the past 12,000 years. *Glob. Ecol. Biogeogr.* **20**, 73–86 (2011).

47. R. N. Holdaway, M. E. Allentoft, C. Jacomb, C. L. Oskam, N. R. Beavan, M. Bunce, An extremely low-density human population exterminated New Zealand moa. *Nat. Commun.* **5**, 5436 (2014).

48. M. Ben-Dor, A. Gopher, I. Hershkovitz, R. Barkai, Man the fat hunter: The demise of *homo erectus* and the emergence of a new hominin lineage in the Middle Pleistocene (ca. 400 kyr) Levant. *PLOS ONE* **6**, e28689 (2011).

49. A. Rhodin, P. Pritchard, P. P. van Dijk, R. Saumure, K. Buhlmann, J. Iverson, R. Mittermeier, *Conservation Biology of Freshwater Turtles and Tortoises* (Chelonian Research Monographs, ed. 1, 2015), vol. 5.

50. B. Voirin, Biology and conservation of the pygmy sloth, *Bradypus pygmaeus*. *J. Mammal.* **96**, 703–707 (2015).

51. SILA–The Greenland Research Centre at the National History Museum of Denmark, Saqqaq culture chronology (2007); https://web.archive.org/web/20110419200203/http://www.natmus.dk/sw18632.asp.

52. D. K. Grayson, The archaeological record of human impacts on animal populations. *J. World Prehist.* **15**, 1–68 (2001).

53. J. M. Wilmshurst, A. J. Anderson, T. F. G. Higham, T. H. Worthy, Dating the late prehistoric dispersal of Polynesians to New Zealand using the commensal Pacific rat. *Proc. Natl. Acad. Sci. U.S.A.* **105**, 7676–7680 (2008).

54. T. Goebel, M. R. Waters, D. H. O'Rourke, The Late Pleistocene dispersal of modern humans in the Americas. *Science* **319**, 1497–1502 (2008).

55. H. S. Groucutt, R. Grün, I. A. S. Zalmout, N. A. Drake, S. J. Armitage, I. Candy, R. Clark-Wilson, J. Louys, P. S. Breeze, M. Duval, L. T. Buck, T. L. Kivell, E. Pomeroy, N. B. Stephens, J. T. Stock, M. Stewart, G. J. Price, L. Kinsley, W. W. Sung, A. Alsharekh, A. Al-Omari, M. Zahir, A. M. Memesh, A. J. Abdulshakoor, A. M. Al-Masari, A. A. Bahameem, K. M. S. Al Murayyi, B. Zahrani, E. L. M. Scerri, M. D. Petraglia, *Homo sapiens* in Arabia by 85,000 years ago. *Nat. Ecol. Evol.* **2**, 800 (2018).

56. M. C. Westaway, J. Olley, R. Grün, At least 17,000 years of coexistence: Modern humans and megafauna at the Willandra Lakes, South-Eastern Australia. *Quat. Sci. Rev*. **157**, 206–211 (2017).

57. M. Aubert, A. Brumm, M. Ramli, T. Sutikna, E. W. Saptomo, B. Hakim, M. J. Morwood, G. D. van den Bergh, L. Kinsley, A. Dosseto, Pleistocene cave art from Sulawesi, Indonesia. *Nature* **514**, 223–227 (2014).

58. C. M. Hurvich, J. S. Simonoff, C.-L. Tsai, Smoothing parameter selection in nonparametric regression using an improved Akaike information criterion. *J. R. Stat. Soc. B*. **60**, 271–293 (1998).

59. S. Lehtonen, D. Silvestro, D. N. Karger, C. Scotese, H. Tuomisto, M. Kessler, C. Peña, N. Wahlberg, A. Antonelli, Environmentally driven extinction and opportunistic origination explain fern diversification patterns. *Sci. Rep.* **7**, 4831 (2017).

60. T. Andermann, S. Faurby, R. Cooke, D. Silvestro, A. Antonelli, iucn_sim: A new program to simulate future extinctions based on IUCN threat status. *bioRxiv* 2019.12.16.878249 (2020).

61. M. Pacifici, L. Santini, M. D. Marco, D. Baisero, L. Francucci, G. G. Marasini, P. Visconti, C. Rondinini, Generation length for mammals. *Nat. Conserv*. **5**, 89–94 (2013).

62. E. W. Goolsby, J. Bruggeman, C. Ané, Rphylopars: Fast multivariate phylogenetic comparative methods for missing data and within-species variation. *Methods Ecol. Evol*. **8**, 22–27 (2017).

63. S. T. Turvey, *Holocene Extinctions* (OUP Oxford, 2009).

64. R. D. E. MacPhee, J. L. White, C. A. Woods, New megalonychid sloths (Phyllophaga, Xenarthra) from the Quaternary of Hispaniola. *Am. Mus. Novit.* **2000**, 1–32 (2000).

65. J. W. Pires-Ferreira, E. Pires-Ferreira, P. Kaulicke, Preceramic animal utilization in the central peruvian anded. *Science* **194**, 483–490 (1976).

66. D. A. McFarlane, R. D. E. MacPhee, Amblyrhiza and the Vertebrate Paleontology of Anguillean Caves. *Bol. Soc. Venezolana Espel.* **27**, 33–38 (1993).

67. J. W. Cain III, P. R. Krausman, H. L. Germaine, Antidorcas marsupialis. *Mamm. Species* **753**, 1–7 (2004).

68. L. H. Soibelzon, E. P. Tonni, M. Bond, The fossil record of South American short-faced bears (Ursidae, Tremarctinae). *J. S. Am. Earth Sci.* **20**, 105–113 (2005).

69. A. J. Winkler, Y. Tomida, New records of the small leporid Aztlanolagus agilis Russell and Harris (Leporidae: Leporinae). *Southwest. Nat.* **33**, 391–396 (1988).

70. B. J. Shockey, R. Salas-Gismondi, P. Baby, J.-L. Guyot, M. C. Baltazar, L. Huaman, A. Clack, M. Stucchi, F. Pujos, J. M. Emerson, J. J. Flynn, New Pleistocene cave faunas of the Andes of central Peru: Radiocarbon ages and the survival of low latitude, Pleistocene DNA. *Palaeontol. Electron.* **12**, 15 (2009).

71. C. Cartelle, W. C. Hartwig, A new extinct primate among the Pleistocene megafauna of Bahia, Brazil. *Proc. Natl. Acad. Sci. U.S.A.* **93**, 6405–6409 (1996).

72. F. Almathen, P. Charruau, E. Mohandesan, J. M. Mwacharo, P. Orozco-terWengel, D. Pitt, A. M. Abdussamad, M. Uerpmann, H.-P. Uerpmann, B. De Cupere, P. Magee, M. A. Alnaqeeb, B. Salim, A. Raziq, T. Dessie, O. M. Abdelhadi, M. H. Banabazi, M. Al-Eknah, C. Walzer, B. Faye, M. Hofreiter, J. Peters, O. Hanotte, P. A. Burger, Ancient and modern DNA reveal dynamics of domestication and cross-continental dispersal of the dromedary. *Proc. Natl. Acad. Sci. U.S.A.* **113**, 6707–6712 (2016).

73. J. de Vos, Pleistocene Deer Fauna in Crete: Its Adaptive Radiation and Extinction. *Tropics* **10**, 125–134 (2000).

74. P. Y. Sondaar, A. A. E. Van Der Geer, Evolution and extinction of Plio-Pleistocene Islands Ungulates, in *Quaternaire,* E. Crégut-Bonnoure, Ed., (2005), pp. 241–256.

75. J. T. Faith, Late Pleistocene and Holocene mammal extinctions on continental Africa. *Earth Sci. Rev.* **128**, 105–121 (2014).

76. L. Kerber, A. Kinoshita, F. A. José, A. M. Graciano Figueiredo, É. V. Oliveira, O. Baffa, Electron Spin Resonance dating of the southern Brazilian Pleistocene mammals from Touro Passo Formation, and remarks on the geochronology, fauna and palaeoenvironments. *Quat. Int.* **245**, 201–208 (2011).

77. A. J. Stuart, A. M. Lister, Extinction chronology of the woolly rhinoceros *Coelodonta antiquitatis* in the context of late Quaternary megafaunal extinctions in northern Eurasia. *Quat. Sci. Rev*. **51**, 1–17 (2012).

78. A. D. Barnosky, E. L. Lindsey, Timing of Quaternary megafaunal extinction in South America in relation to human arrival and climate change. *Quat. Int.* **217**, 10–29 (2010).

79. J. T. Faith, J. N. Choiniere, C. A. Tryon, D. J. Peppe, D. L. Fox, Taxonomic status and paleoecology of *Rusingoryx atopocranion* (Mammalia, Artiodactyla), an extinct Pleistocene bovid from Rusinga Island, Kenya. *Quat. Res.* **75**, 697–707 (2011).

80. F. J. Prevosti, M. A. Ramírez, M. Schiaffini, F. Martin, D. E. Udrizar Sauthier, M. Carrera, C. Sillero-Zubiri, U. F. J. Pardiñas, Extinctions in near time: New radiocarbon dates point to a very recent disappearance of the South American fox *Dusicyon avus* (Carnivora: Canidae). *Biol. J. Linn. Soc.* **116**, 704–720 (2015).

81. A. S. Tesakov, V. V. Titov, N. B. Leonova, A. A. Velichko, A. N. Simakova, A. S. Zastrozhnov, P. H. Frolov, Quaternary stratigraphy and paleontology of the Southern Russia: connections between Europe, Africa and Asia in *Programme and Guidebook of Excursions of the International INQUA-SEQS Conference* (2010), 52 p.

82. D. Mol, J. D. Vos, J. van der Plicht, The presence and extinction of *Elephas antiquus* Falconer and Cautley, 1847, in Europe. *Quat. Int.* **169–170**, 149–153 (2007).

83. N. E. Todd, Trends in proboscidean diversity in the African Cenozoic. *J. Mamm. Evol.* **13**, 1–10 (2006).

84. G. Mangano, L. Bonfiglio, First finding of a partially articulated elephant skeleton from a Late Pleistocene hyena den in Sicily (San Teodoro Cave, North Eastern Sicily, Italy). *Quat. Int.* **276–277**, 53–60 (2012).

85. Y. A. Park, H. I. Yi, Late Quaternary climatic changes and sea-level history along the Korean coasts. *J. Coast. Res.*, 163–168 (1995).

86. A. Iwase, J. Hashizume, M. Izuho, K. Takahashi, H. Sato, Timing of megafaunal extinction in the late Late Pleistocene on the Japanese Archipelago. *Quat. Int.* **255**, 114–124 (2012).

87. J. Weinstock, E. Willerslev, A. Sher, W. Tong, S. Y. W. Ho, D. Rubenstein, J. Storer, J. Burns, L. Martin, C. Bravi, A. Prieto, D. Froese, E. Scott, L. Xulong, A. Cooper, Evolution,

systematics, and phylogeography of Pleistocene horses in the new world: A molecular perspective. *PLoS Biol.* **3**, e241 (2005).

88. L. A. Borrero, M. Zárate, L. Miotti, M. Massone, The Pleistocene – Holocene Transition and Human Occupations in the Southern Cone of South America. *Quat. Int.* **49–50**, 191–199 (1998).

89. B. Bougariane, S. Zouhri, B. Ouchaou, A. Oujaa, L. Boudad, Large mammals from the Upper Pleistocene at Tamaris i 'Grotte des gazelles' (Casablanca, Morocco): Paleoecological and biochronological implications. *Hist. Biol.* **22**, 295–302 (2010).

90. K. de O. Porpino, J. C. Fernicola, L. P. Bergqvist, A new cingulate (Mammalia: Xenarthra), *Pachyarmatherium brasiliense* sp. nov., from the Late Pleistocene of Northeastern Brazil. *J. Vertebr. Paleontol.* **29**, 881–893 (2009).

91. A. A. Carlini, A. E. Zurita, O. A. Aguilera, North American Glyptodontines (Xenarthra, Mammalia) in the Upper Pleistocene of northern South America. *Palaontol. Z.* **82**, 125–138 (2008).

92. J. Daura, M. Sanz, R. Julià, D. García-Fernández, J. J. Fornós, M. Vaquero, E. Allué, J. M. López-García, H. A. Blain, J. E. Ortiz, T. Torres, R. M. Albert, À. Rodríguez-Cintas, A. Sánchez-Marco, E. Cerdeño, A. R. Skinner, Y. Asmeron, V. J. Polyak, J. Zilhão, Cova del Rinoceront (Castelldefels, Barcelona): A terrestrial record for the Last Interglacial period (MIS 5) in the Mediterranean coast of the Iberian Peninsula. *Quat. Sci. Rev*. **114**, 203–227 (2015).

93. J. T. Faith, T. A. Surovell, Synchronous extinction of North America's Pleistocene mammals. *Proc. Natl. Acad. Sci. U.S.A.* **106**, 20641–20645 (2009).

94. F. Rivals, R. Blasco, Presence of *Hemitragus aff. cedrensis* (Mammalia, Bovidae) in the Iberian Peninsula: Biochronological and biogeographical implications of its discovery at Bolomor Cave (Valencia, Spain). *Comptes Rendus - Palevol*. **7**, 391–399 (2008).

95. L. Orlando, J. L. Metcalf, M. T. Alberdi, M. Telles-Antunes, D. Bonjean, M. Otte, F. Martin, V. Eisenmann, M. Mashkour, F. Morello, J. L. Prado, R. Salas-Gismondi, B. J. Shockey, P. J. Wrinn, S. K. Vasil'ev, N. D. Ovodov, M. I. Cherry, B. Hopwood, D. Male, J. J. Austin, C. Hänni, A. Cooper, Revising the recent evolutionary history of equids using ancient DNA. *Proc. Natl. Acad. Sci. U.S.A.* **106**, 21754–21759 (2009).

96. C. Der Sarkissian, J. T. Vilstrup, M. Schubert, A. Seguin-Orlando, D. Eme, J. Weinstock, M. T. Alberdi, F. Martin, P. M. Lopez, J. L. Prado, A. Prieto, C. J. Douady, T. W. Stafford, E. Willerslev, L. Orlando, Mitochondrial genomes reveal the extinct *Hippidion* as an outgroup to all living equids. *Biol. Lett.* **11**, 20141058 (2015).

97. A. D. Rincón, R. S. White, H. G. Mcdonald, Late Pleistocene cingulates (Mammalia: Xenarthra) from Mene de Inciarte Tar Pits, Sierra de Perijá, western Venezuela. *J. Vertebr. Paleontol*. **28**, 197–207 (2008).

98. E. C. Holanda, J. Ferigolo, A. M. Ribeiro, New *Tapirus* species (Mammalia: Perissodactyla: Tapiridae) from the upper Pleistocene of Amazonia, Brazil. *J. Mammal.* **92**, 111–120 (2011).

99. D. Reich, R. E. Green, M. Kircher, J. Krause, N. Patterson, E. Y. Durand, B. Viola, A. W. Briggs, U. Stenzel, P. L. F. Johnson, T. Maricic, J. M. Good, T. Marques-Bonet, C. Alkan, Q. Fu, S. Mallick, H. Li, M. Meyer, E. E. Eichler, M. Stoneking, M. Richards, S. Talamo, M. V. Shunkov, A. P. Derevianko, J.-J. Hublin, J. Kelso, M. Slatkin, S. Pääbo, Genetic history of an archaic hominin group from Denisova Cave in Siberia. *Nature* **468**, 1053–1060 (2010).

100. P. Brown, T. Sutikna, M J Morwood, R P Soejono, Jatmiko, E. W. Saptomo, R. A. Due, A new small-bodied hominin from the Late Pleistocene of Flores, Indomesia. *Nature* **431**, 1055–1061 (2004).

101. C. Finlayson, F. G. Pacheco, J. Rodríguez-Vidal, D. A. Fa, J. M. G. López, A. S. Pérez, G. Finlayson, E. Allue, J. B. Preysler, I. Cáceres, J. S. Carrión, Y. F. Jalvo, C. P. Gleed-Owen, F. J. Jimenez Espejo, P. López, J. A. L. Sáez, J. A. R. Cantal, A. S. Marco, F. G. Guzman, K. Brown, N. Fuentes, C. A. Valarino, A. Villalpando, C. B. Stringer, F. M. Ruiz, T. Sakamoto, Late survival of Neanderthals at the southernmost extreme of Europe. *Nature* **443**, 850–853 (2006).

102. J. W. F. Reumer, L. Rook, K. Van Der Borg, K. Post, D. Mol, J. De Vos, Late Pleistocene survival of the saber-toothed cat Homotherium in Northwestern Europe. *J. Vertebr. Paleontol.* **23**, 260–262 (2003).

103. C. Widga, T. L. Fulton, L. D. Martin, B. Shapiro, Homotherium serum and cervalces from the great lakes region, USA: Geochronology, morphology and ancient DNA. *Boreas* **41**, 546–556 (2012).

104. H. W. Tong, Quaternary Hystrix (Rodentia, Mammalia) from North China: Taxonomy, stratigraphy and zoogeography, with discussions on the distribution of Hystrix in Palearctic Eurasia. *Quat. Int.* **179**, 126–134 (2008).

105. H. Monchot, P. Fernandez, J.-M. Gaillard, Paleodemographic analysis of a fossil porcupine (Hystrix refossa Gervais, 1852) population from the Upper Pleistocene site of Geula Cave (Mount Carmel, Israel). *J. Archaeol. Sci.* **39**, 3027–3038 (2012).

106. B. E. Crowley, A refined chronology of prehistoric Madagascar and the demise of the megafauna. *Quat. Sci. Rev.* **29**, 2591–2603 (2010).

107. J. L. Prado, C. Martinez-Maza, M. T. Alberdi, Megafauna extinction in South America: A new chronology for the Argentine Pampas. *Palaeogeogr. Palaeoclimatol. Palaeoecol.* **425**, 41–49 (2015).

108. H. Yang, E. M. Golenberg, J. Shoshani, Phylogenetic resolution within the Elephantidae using fossil DNA sequence from the American mastodon (Mammut americanum) as an outgroup. *Proc. Natl. Acad. Sci. U.S.A.* **93**, 1190–1194 (1996).

109. D. D. Gillette, D. B. Madsen, The Columbian Mammoth, Mammuthus columbi, from the Wasatch Mountains of Central Utah. *Paleontol. Soc.* **67**, 669–680 (1993).

110. L. D. Agenbroad, in *Contributions to the Geology of the Northern Channel Islands, So. California* (Pacific Section American Association of Petroleum Geologists, 1998).

111. R. D. E. MacPhee, C. Flemming, A Possible Heptaxodontine and Other Caviidan Rodents from the Quaternary of Jamaica. *Am. Mus. Novit.* **3422**, 1–42 (2003).

112. R. G. Roberts, T. F. Flannery, L. K. Ayliffe, H. Yoshida, J. M. Olley, G. J. Prideaux, G. M. Laslett, A. Baynes, M. A. Smith, R. Jones, B. L. Smith, New ages for the last Australian megafauna: Continent-wide extinction about 46,000 years ago. *Science* **292**, 1888–1892 (2001).

113. S. Merzoug, L. Sari, Re-examination of the Zone I material from Tamar Hat (Algeria): Zooarchaeological and technofunctional analyses. *Afr. Archaeol. Rev.* **25**, 57–73 (2008).

114. S. T. Turvey, S. Brace, M. Weksler, A new species of recently extinct rice rat (Megalomys) from Barbados. *Mamm. Biol.* **77**, 404–413 (2012).

115. G. Xing, H. Wanbo, X. Ziqiang, M. Zhibang, J. W. Olsen, 120-150 ka human tooth and ivory engravings from Xinglongdong Cave, Three Gorges Region, South China. *Chin. Sci. Bull.* **49**, 175–180 (2004).

116. M. Coltorti, L. Abbazzi, M. P. Ferretti, P. Iacumin, F. P. Rios, M. Pellegrini, P. Pieruccini, M. Rustioni, G. Tito, L. Rook, Last Glacial mammals in South America: A new scenario from the Tarija Basin (Bolivia). *Naturwissenschaften* **94**, 288–299 (2007).

117. K. Cruz-Uribe, The Mammalian fauna from Recliff Cave, Zimbabwe. *S. Afr. Archaeol. Bull.* **38**, 7–16 (1983).

118. M. Ubilla, D. Perea, C. G. Aguilar, N. Lorenzo, Late Pleistocene vertebrates from northern Uruguay: Tools for biostratigraphic, climatic and environmental reconstruction. *Quat. Int.* **114**, 129–142 (2004).

119. S. R. Stinnesbeck, E. Frey, W. Stinnesbeck, J. A. Olguín, P. Zell, A. T. Mata, M. B. Sanvicente, A. G. González, C. R. Sandoval, E. A. Nuñez, A new fossil peccary from the Pleistocene-Holocene boundary of the eastern Yucatán Peninsula, Mexico. *J. S. Am. Earth Sci.* **77**, 341–349 (2017).

120. D. W. Steadman, P. S. Martin, Ross D. E. Mac Phee, A. J. T. Jull, H. G. McDonald, C. A. Woods, M. Iturralde-Vinent, G. W. L. Hodgins, Asynchronous extinction of late Quaternary sloths on continents and islands. *Proc. Natl. Acad. Sci.* **102**, 11763–11768 (2005).

121. G. G. Politis, P. G. Messineo, The Campo Laborde site: New evidence for the Holocene survival of Pleistocene megafauna in the Argentine Pampas. *Quat. Int.* **191**, 98–114 (2008).

122. P. Y. Sondaar, M. Sanges, T. Kotsakis, P. L. de Boer, The Pleistocene deer hunter of Sardinia. *Geobios* **19**, 17–31 (1986).

123. E. Soibelzon, L. S. Avilla, M. Castro, The cingulates (Mammalia: Xenarthra) from the late Quaternary of northern Brazil: Fossil records, paleoclimates and displacements in America. *Quat. Int.* **377**, 118–125 (2015).

124. R. D. E. MacPhee, D. A. Burney, N. A. Wells, Early Holocene chronology and environment of Ampasambazimba, A Malagasy subfossil lemur site. *Int. J. Primatol.* **6**, 463–489 (1985).

125. L. R. Godfrey, W. L. Jungers, D. A. Burney, Subfossil lemurs of Madagascar. *Cenozoic mammals of Africa*, 351–367 (2010).

126. M. Ubilla, D. Perea, A. Rinderknecht, A. Corona, Pleistocene mammals from Uruguay: Biostratigraphic, biogeographic and environmental connotations. *SBP Monografias* (2005).

127. R. Barnett, B. Shapiro, I. Barnes, S. Y. W. Ho, J. Burger, N. Yamaguchi, T. F. G. Higham, H. T. Wheeler, W. Rosendahl, A. V. Sher, M. Sotnikova, T. Kuznetsova, G. F. Baryshnikov, L. D. Martin, C. Richard Harington, J. A. Burns, A. Cooper, Phylogeography of lions (Panthera leo ssp.) reveals three distinct taxa and a late Pleistocene reduction in genetic diversity. *Mol. Ecol.* **18**, 1668–1677 (2009).

128. W. Suarez, S. Diaz-Franco, A New Fossil Bat (Chiroptera : Phyllostomidae) from a Quaternary Cave Deposit in Cuba. *Caribb. J. Sci.* **39**, 371–377 (2003).

129. J. Hansford, J. M. Nuñez-Miño, R. P. Young, S. Brace, J. L. Brocca, S. T. Turvey, Taxonomy-testing and the “Goldilocks Hypothesis”: Morphometric analysis of species diversity in living and extinct Hispaniolan hutias. *Syst. Biodivers.* **10**, 491–507 (2012).

130. G. Cuenca-Bescós, L. G. Straus, M. R. González Morales, J. C. García Pimienta, The reconstruction of past environments through small mammals: From the Mousterian to the Bronze Age in El Mirón Cave (Cantabria, Spain). *J. Archaeol. Sci.* **36**, 947–955 (2009).

131. M. L. Cupper, J. Duncan, Last glacial megafaunal death assemblage and early human occupation at Lake Menindee, southeastern Australia. *Quat. Res.* **66**, 332–341 (2006).

132. C. S. M. Turney, T. F. Flannery, R. G. Roberts, C. Reid, L. K. Fifield, T. F. G. Higham, Z. Jacobs, N. Kemp, E. A. Colhoun, R. M. Kalin, N. Ogle, Late-surviving megafauna in Tasmania, Australia, implicate human involvement in their extinction. *Proc. Natl. Acad. Sci.* **105**, 12150–12153 (2008).

133. T. F. Flannery, M. J. Mountain, K. Aplin, Quaternary kangaroos (Macropodidae: Marsupialia) from Nombe rock shelter, Papua New Guinea, with comments on the nature of

megafaunal extinction in the New Guinea Highlands. *Proc. Linnean Soc. NSW* **107**, 75–97 (1983).

134. F. J. Prevosti, E. P. Tonni, J. C. Bidegain, Stratigraphic range of the large canids (Carnivora, Canidae) in South America, and its relevance to quaternary biostratigraphy. *Quat. Int.* **210**, 76–81 (2009).

135. B. Kurtén, The stilt-legged deer Sangamona of the North-American Pleistocene. *Boreas* **8**, 313–321 (1979).

136. G. Ficcarelli, M. Coltorti, M. Moreno-Espinosa, P. L. Pieruccini, L. Rook, D. Torre, A model for the Holocene extinction of the mammal megafauna in Ecuador. *J. S. Am. Earth Sci.* **15**, 835–845 (2003).

137. I. A. Vislobokova, Historical development and geographical distribution of giant deer (Cervidae, Megacerini). *Paleontol. J.* **45**, 674–688 (2011).

138. E. S. Vrba, F. Bibi, A. G. Costa, First Asian record of a late Pleistocene reduncine (Artiodactyla, Bovidae, Reduncini), Sivacobus sankaliai, sp. Nov., from Gopnath (Miliolite Formation) Gujarat, India, and a revision of the Asian genus Sivacobus Pilgrim, 1939. *J. Vertebr. Paleontol.* **35**, e943399 (2015).

139. S. T. Turvey, H. Tong, A. J. Stuart, A. M. Lister, Holocene survival of Late Pleistocene megafauna in China: A critical review of the evidence. *Quat. Sci. Rev.* **76**, 156–166 (2013).

140. J. R. Stewart, Neanderthal extinction as part of the faunal change in Europe during Oxygen Isotope Stage 3. *Acta Zool. Cracov.* **50A**, 93–124 (2007).

141. G. J. Price, G. E. Webb, J.-x. Zhao, Y.-x. Feng, A. S. Murray, B. N. Cooke, S. A. Hocknull, I. H. Sobbe, Dating megafaunal extinction on the Pleistocene Darling Downs, eastern Australia: The promise and pitfalls of dating as a test of extinction hypotheses. *Quat. Sci. Rev.* **30**, 899–914 (2011).

142. M. Pacher, A. J. Stuart, Extinction chronology and palaeobiology of the cave bear (Ursus spelaeus). *Boreas* **38**, 189–206 (2009).

143. A. Hubbe, P. M. Haddad-Martim, M. Hubbe, E. L. Mayer, A. Strauss, A. S. Auler, L. B. Piló, W. A. Neves, Identification and importance of critical depositional gaps in pitfall cave

environments: The fossiliferous deposit of Cuvieri Cave, eastern Brazil. *Palaeogeogr. Palaeoclimatol. Palaeoecol.* **312**, 66–78 (2011).

144. R. C. Ribeiro, A. Kinoshita, A. M. G. Figueiredo, I. S. Carvalho, O. Baffa, Electron Spin Resonance dating of the Late Quaternary megafauna fossils from Baixa Grande, Bahia, Brazil. *Quat. Int.* **305**, 91–96 (2012).
